# Two Alleles of NF-κB in the Sea Anemone *Nematostella vectensis* Are Widely Dispersed in Nature and Encode Proteins with Distinct Activities

**DOI:** 10.1371/journal.pone.0007311

**Published:** 2009-10-06

**Authors:** James C. Sullivan, Francis S. Wolenski, Adam M. Reitzel, Courtney E. French, Nikki Traylor-Knowles, Thomas D. Gilmore, John R. Finnerty

**Affiliations:** Department of Biology, Boston University, Boston, Massachusetts, United States of America; Max Planck Institute for Evolutionary Anthropology, Germany

## Abstract

**Background:**

NF-κB is an evolutionarily conserved transcription factor that controls the expression of genes involved in many key organismal processes, including innate immunity, development, and stress responses. NF-κB proteins contain a highly conserved DNA-binding/dimerization domain called the Rel homology domain.

**Methods/Principal Findings:**

We characterized two NF-κB alleles in the sea anemone *Nematostella vectensis* that differ at nineteen single-nucleotide polymorphisms (SNPs). Ten of these SNPs result in amino acid substitutions, including six within the Rel homology domain. Both alleles are found in natural populations of *Nematostella*. The relative abundance of the two NF-κB alleles differs between populations, and departures from Hardy-Weinberg equilibrium within populations indicate that the locus may be under selection. The proteins encoded by the two Nv-NF-κB alleles have different molecular properties, in part due to a Cys/Ser polymorphism at residue 67, which resides within the DNA recognition loop. In nearly all previously characterized NF-κB proteins, the analogous residue is fixed for Cys, and conversion of human RHD proteins from Cys to Ser at this site has been shown to increase DNA-binding ability and increase resistance to inhibition by thiol-reactive compounds. However, the naturally-occurring *Nematostella* variant with Cys at position 67 binds DNA with a higher affinity than the Ser variant. On the other hand, the Ser variant activates transcription in reporter gene assays more effectively, and it is more resistant to inhibition by a thiol-reactive compound. Reciprocal Cys<->Ser mutations at residue 67 of the native Nv-NF-κB proteins affect DNA binding as in human NF-κB proteins, e.g., a Cys->Ser mutation increases DNA binding of the native Cys variant.

**Conclusions/Significance:**

These results are the first demonstration of a naturally occurring and functionally significant polymorphism in NF-κB in any species. The functional differences between these alleles and their uneven distribution in the wild suggest that different genotypes could be favored in different environments, perhaps environments that vary in their levels of peroxides or thiol-reactive compounds.

## Introduction

The transcription factor NF-κB regulates a broad range of biological processes including innate and adaptive immunity, cell growth, differentiation, apoptosis, and tumorigenesis [1]. In addition, NF-κB is involved in mediating cellular responses to numerous environmental stressors. Stressors that can activate the NF-κB pathway in insects and vertebrates include pathogens, ultraviolet light, oxidative stress, and shear stress. Given that the same stressors can activate the NF-κB pathway in insects and vertebrates, the role of NF-κB in combating stress must predate the radiation of triploblastic animals, a process that was already well underway during the Cambrian explosion (542–525 million years ago).

The NF-κB signaling pathway is primarily controlled by subcellular location. In response to an appropriate stressor or stimulus, NF-κB is released from a latent cytoplasmic state and enters the nucleus to activate the transcription of a diverse set of effector genes including ones encoding antimicrobial peptides, mucin, heat-shock factors, and anti-oxidant proteins [1], [2]. Target genes of NF-κB contain DNA-binding sites (‘κB sites’) in their promoters/enhancers.

NF-κB binds to a κB site as a dimer via sequences in the conserved DNA-binding/dimerization domain called the Rel homology domain (RHD) [3]. The presence of an RHD characterizes a superfamily of proteins that includes the Rel/NF-κB family and the NFAT family. Multiple RHD proteins have been identified in the genomes of individual protostomes and deuterostomes. The human genome encodes ten RHD proteins (NF-κB1, NF-κB2, Rel, RelA, RelB and NFAT1-5), and the *Drosophila* genome encodes four RHD proteins (Dorsal, Dif, Relish, and NFAT). No RHD proteins are encoded in the sequenced genomes of fungi or the choanoflagellate *Monosiga brevicolis*, suggesting that the RHD domain originated early in metazoan evolution [4], [5]. A single RHD-containing NF-κB-like protein was identified in the demosponge *Amphimedon queenslandica* [4]; however, no NFAT-like protein has yet been found in *Amphimedon* or any other sponge. The presence of distinct NF-κB and NFAT proteins in two cnidarians—the sea anemone *Nematostella vectensis* and the coral *Acropora millepora—*indicates that the RHD proteins had begun to diversify prior to the cnidarian-triploblast divergence, which occurred ∼700 million years ago [6], [7].

**Figure 1:**
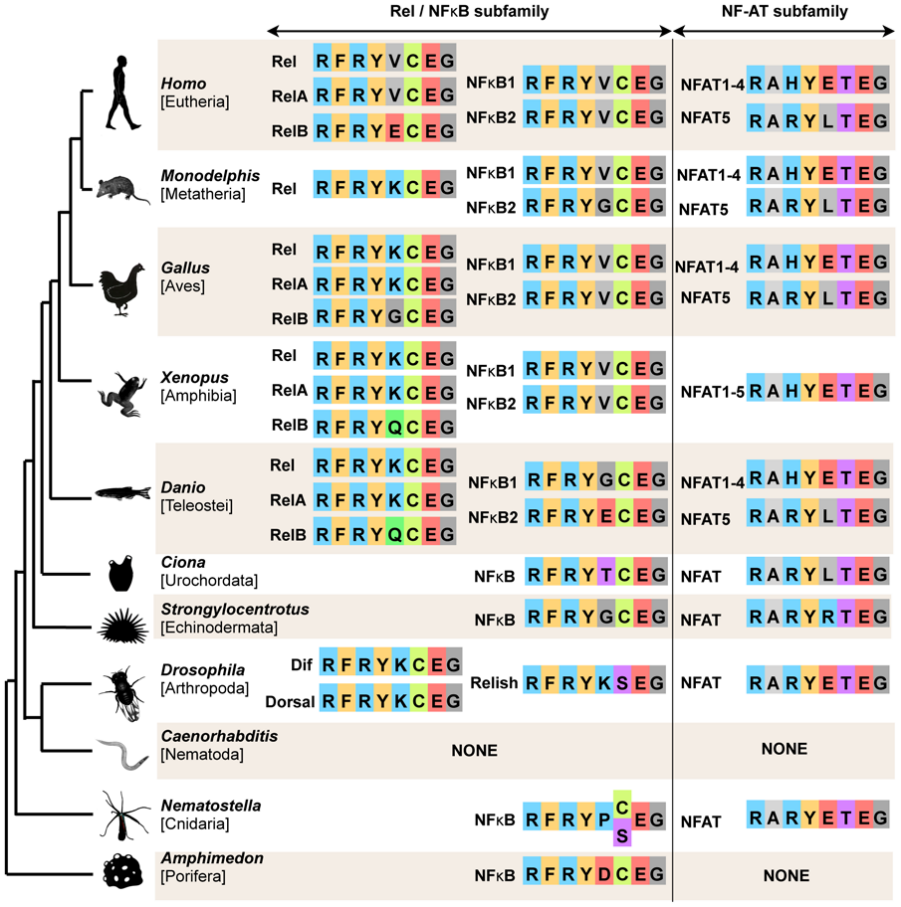
Phylogenetic relationships among major metazoan lineages and sequence of the DNA recognition loop in Rel Homology Domain proteins. The phylogeny depicts the evolutionary relationships among taxa whose genomes have been sequenced. Multiple RHD proteins are encoded in the genomes of all of these animals except the nematode *Caenorhabditis elegans*, which appears to lack RHD proteins entirely, and the sponge, *Amphimedon queenslandica*, which appears to possess only a single RHD protein with similarity to NF-κB. The amino acid sequence of the DNA recognition loop is shown for every known RHD protein in these animals.

NF-κB dimers bind to DNA with high affinity and make multiple contacts with the κB site [8]. One highly conserved element required for DNA binding by all NF-κB proteins is a DNA recognition loop, which has the consensus sequence RFRYXCEG. Almost all known members of the Rel/NF-κB subfamily, including the NF-κBs of *Amphimedon* and *Acropora*, have Cys at position 6 of this sequence (Figure 1; Figure S1). Only the Relish protein in several insects and the previously reported *Nematostella* NF-κB protein (Nv-NF-κB) have Ser at position 6 [6], [9], [10]. All known NFAT proteins have Thr at this position (Figure 1).

In some human RHD-containing proteins, a Cys-to-Ser mutation at position 6 of the DNA recognition loop has functional consequences. A Cys-to-Ser mutation at this position in human RelA or c-Rel greatly increases the DNA-binding activity of each protein [11]–[13]. In addition, certain thiol-reactive compounds can inhibit DNA binding by wild-type Cys-containing RelA or c-Rel, but the same compounds do not affect the corresponding Cys-to-Ser mutants [12]–[16]. Furthermore, conversion of the Cys to Ser can render NF-κB proteins resistant to redox regulation. The thiol group in the Cys of the DNA recognition loop has been shown to be important for DNA binding [11], [17], [18], with this residue (Cys62 in human p50) being a known site of redox control [14], [18]–[23].

In this paper, we show that two Nv-NF-κB alleles encoding proteins with different DNA-binding and transactivation properties are present in wild *Nematostella* populations. Both alleles are widely distributed along both the Atlantic and Pacific coasts of the US. This is the first demonstration of a highly prevalent functional polymorphism within an NF-κB protein in any species. The functional differences between these variants suggest that departures from Hardy-Weinberg equilibrium observed in natural populations are due, at least partially, to selection acting on this locus.

## Results

### The presence of multiple NF-κB alleles in *Nematostella*

**Figure 2:**
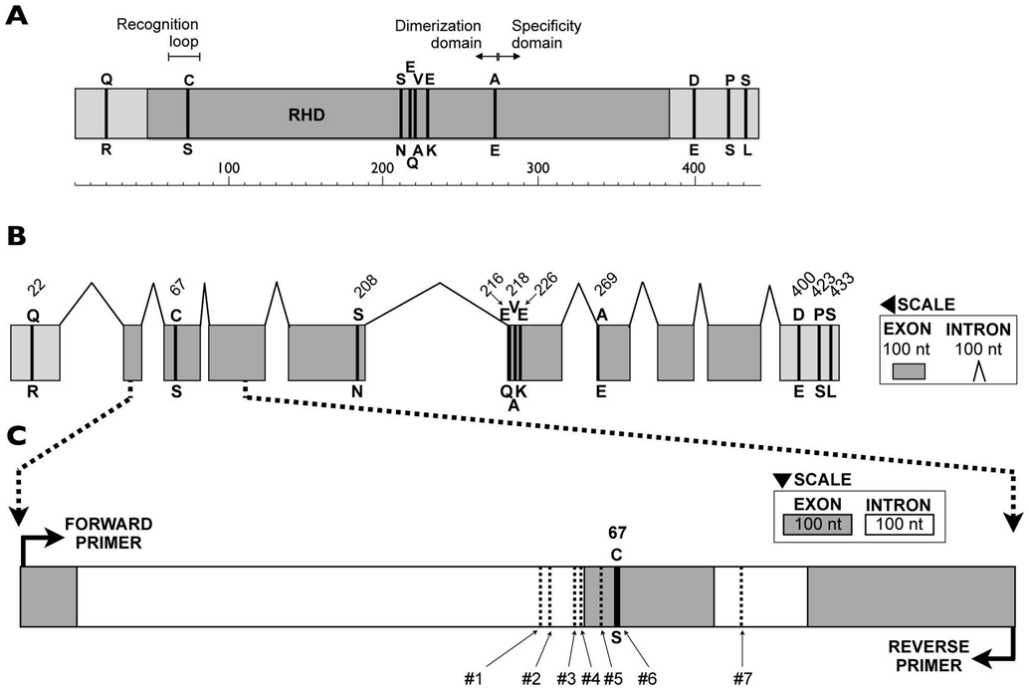
Location of polymorphic positions in the Nv-NF-κB protein and gene. (A) As indicated, ten protein-coding differences were identified in a comparison of laboratory strain-derived Ser67 and Cys67 alleles. Six of the variable residues are in the highly conserved Rel Homology Domain (RHD). At each variable position, the amino acid encoded by the Cys67 allele is depicted above the diagram, and the amino acid encoded by the Ser67 allele is depicted below the diagram. The full-length Nv-NF-κB protein is 440 amino acids long (scale bar at bottom). (B) The locations of polymorphic amino acids are shown relative to the structure of the Nv-NF-κB gene. (C) The region of the Nv-NF-κB gene amplified in the population genetic survey extends from exon two to exon four, including two introns. The variable nucleotide positions identified in this survey are numbered from 1–7.

When we initially identified NF-κB transcripts among *Nematostella* expressed sequence tags (ESTs) that were generated as part of the genome-sequencing project [24], we noted a Cys/Ser polymorphism at residue 67 (position 6 of the DNA recognition loop). Three of the eight NF-κB ESTs encode a Cys at this position (RFRYPCEG), while the other five ESTs encode a Ser (RFRYPSEG). This polymorphism is of interest because the presence of either Cys or Ser in this position of the DNA recognition loop has been previously shown to impact DNA-binding activity [11]–[13], redox regulation, and the effect of thiol-reactive compounds [12]–[16] in certain vertebrate RHD proteins. To determine whether there are additional differences between these two alleles, we cloned and sequenced cDNAs for the entire protein-coding region for each allele from laboratory animals. This analysis revealed 19 nucleotide differences between the two cDNAs within the coding region, ten of which result in amino acid substitutions (Figure 2A; Figure S2). For simplicity, we will refer to these two alleles as the Cys and Ser variants.

As the degree of evolutionary conservation can predict which sequence variants are likely to have the most pronounced phenotypic consequences [25], we compared all of the Nv-NF-κB protein-coding polymorphisms against the homologous positions in other taxa [6]. Five of the amino acid polymorphisms in *Nematostella* reside in relatively rapidly evolving portions of the protein that cannot be unambiguously aligned with homologous positions in distantly related animals (22:Q/R, 208:S/N, 400:D/E, 423:P/S, 433:S/L; Figure 2). Four of the polymorphisms reside at positions that vary among human NF-κB/Rel proteins (216:E/Q, 218:V/A, 226:E/K, 269:A/E). By contrast, the Cys/Ser polymorphism at position 67, which resides within the DNA recognition loop, occurs at one of the most evolutionary conserved positions in the RHD superfamily of proteins. The homologous position is occupied by a Cys in every other reported member of the NF-κB/Rel family, with the exception of the Relish protein of several insect species (Figure 1; Figure S1).

**Figure 3:**
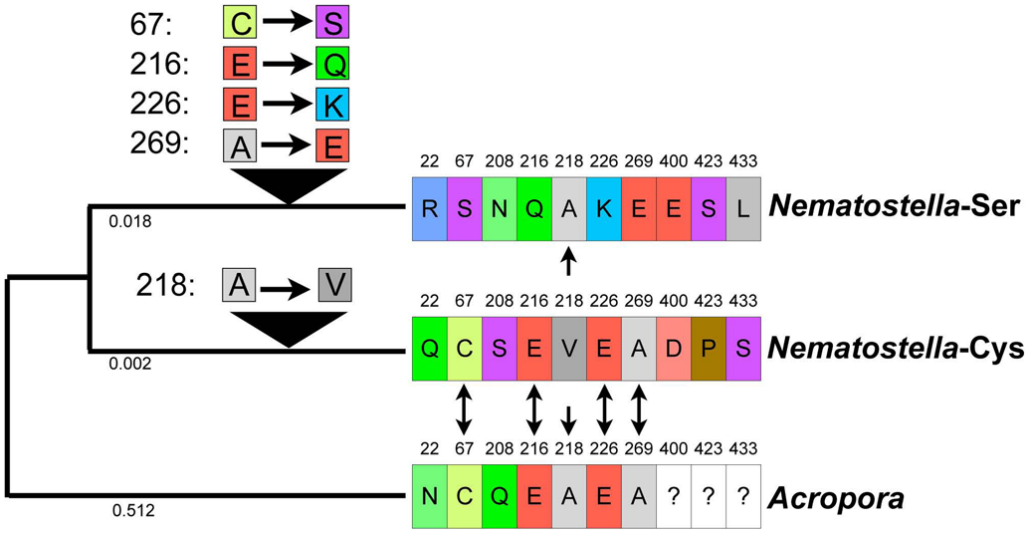
Inferred evolution of polymorphic positions. A partial coding sequence of the coral *Acropora millepora* was used to root a phylogenetic tree relating the two *Nematostella* alleles. A neighbor-joining tree was constructed using the computer program Phylip (version 3.6). Distances between sequences were computed using the first 330 amino acids of the alignment (shown in Figure S3) and the JTT distance matrix. Numbers below branches indicate phylogenetic distance (in units of expected number of substitutions per residue). Positions that are polymorphic in *Nematostella* are shown to the right. The *Acropora* sequence was identical to one of the two *Nematostella* variants at five positions (double-headed arrows). For each of these five positions, substitutions were mapped to either the branch leading to the Ser allele or the branch leading to the Cys allele, assuming that the condition found in *Acropora* is the ancestral state.

We were also able to align seven of the ten polymorphic residues in Nv-NF-κB with a partial protein sequence from the coral *Acropora millepora*; this is the most closely related species to *Nematostella* for which a published NF-κB sequence is available (Figure S3). At four of these seven variable positions, including the Cys/Ser polymorphism in the DNA recognition loop, the coral sequence is identical to the Cys allele of *Nematostella*. At one position, the coral protein is identical to the Ser allele, and at the final two positions, the coral protein differs from both *Nematostella* alleles. If we root the evolution of the *Nematostella* alleles using the coral sequence, we can infer that the Ser allele of *Nematostella* is derived from an ancestral Cys allele, and that the Ser allele experienced non-conservative substitutions at positions 216, 226, and 229 (Figure 3).

### Different Nv-NF-κB alleles are present in wild populations of *Nematostella*

Most laboratory strains of *Nematostella* and all of the ESTs generated by the genome sequencing project were derived from a long-standing laboratory culture initiated with animals collected from Rhode River, Maryland, ∼20 years ago [26]. To determine whether Cys and Ser alleles are both represented in natural populations and if so, to characterize the natural distribution of these alleles, we compared a 159-nucleotide fragment of the Nv-NF-κB gene from 403 animals collected from 23 populations throughout *Nematostella's* global range (Table 1; Figure S4).

**Table 1:** Populations Sampled and Distribution of Cys/Ser Alleles and Genotypes.

SITE	N	C1	C2	C3	C4	C (tot)	Freq.Cys.	S	Freq Ser.	CC	CS	SS	H_obs_	H_exp_	(H_exp_ H_obs_)/H_exp_	^1^Depar HWE?
1.Chezzetcook, NS	26	21	0	0	3	24	0.46	28	0.54	1	22	3	0.85	0.50	−0.70	p<0.005
2. Peggy's Cove, NS	9	2	0	0	0	2	0.11	16	0.89	1	0	8	0.00	0.20	1.00	p<0.005
3. Mahone Bay, NS	19	37	0	0	1	38	1.00	0	0.00	19	0	0	0.00	0.00	n/a	
4. Crescent Beach, NS	16	17	0	0	1	18	0.56	14	0.44	2	14	0	0.88	0.49	−0.78	p<0.005
5. Noel, NS	10	18	0	0	0	18	0.90	2	0.10	9	0	1	0.00	0.18	1.00	p<0.005
6. Kingsport, NS	35	55	14	0	1	70	1.00	0	0.00	35	0	0	0.00	0.00	n/a	
7. Odiorne Point, NH	12	24	0	0	0	24	1.00	0	0.00	12	0	0	0.00	0.00	n/a	
8. Rye Harbor, NH	6	12	0	0	0	12	1.00	0	0.00	6	0	0	0.00	0.00	n/a	
9. Wallis Sands, NH	21	42	0	0	0	42	1.00	0	0.00	21	0	0	0.00	0.00	n/a	
10. Crane, MA	12	24	0	0	0	24	1.00	0	0.00	12	0	0	0.00	0.00	n/a	
11. Neponset, MA	23	46	0	0	0	46	1.00	0	0.00	23	0	0	0.00	0.00	n/a	
12. Pocasset, MA	15	29	1	0	0	30	1.00	0	0.00	15	0	0	0.00	0.00	n/a	
13. Sippewisset, MA	49	95	0	0	0	95	0.97	3	0.03	46	3	0	0.06	0.06	−0.03	ns
14. Meadowlands, NJ	8	16	0	0	0	16	1.00	0	0.00	8	0	0	0.00	0.00	n/a	
15. Rhode River, MD	61	73	0	0	0	73	0.60	49	0.40	23	27	11	0.44	0.48	0.08	ns
16. Baruch, SC	23	28	0	18	0	46	1.00	0	0.00	23	0	0	0.00	0.00	n/a	
17. San Juan, WA	12	24	0	0	0	24	1.00	0	0.00	12	0	0	0.00	0.00	n/a	
18. Coos Bay, OR	1	2	0	0	0	2	1.00	0	0.00	1	0	0	0.00	0.00	n/a	
19. Humboldt Bay, CA	8	13	0	0	0	13	0.81	3	0.19	5	3	0	0.38	0.30	−0.23	ns
20. San Francisco, CA	5	8	0	0	0	8	0.80	2	0.20	3	2	0	0.40	0.32	−0.25	ns
21. Gilkicker, UK	9	9	9	0	0	18	1.00	0	0.00	9	0	0	0.00	0.00	n/a	
22. Near Salterns, UK	14	14	14	0	0	28	1.00	0	0.00	14	0	0	0.00	0.00	n/a	
23. Salterns, UK	9	9	9	0	0	18	1.00	0	0.00	9	0	0	0.00	0.00	n/a	
**TOTAL**	**403**	**618**	**47**	**18**	**6**	**689**	**0.85**	**117**	**0.15**	**309**	**71**	**23**	**0.18**	**0.25**	**0.29**	
**NOVA SCOTIA**	115	150	14	0	6	170	0.74	60	0.26	67	36	12	0.31	0.39	0.19	
**NEW ENGLAND**	138	272	1	0	0	273	0.99	3	0.01	135	3	0	0.02	0.02	−0.01	
**MID-ATLANTIC**	31	44	0	18	0	62	1.00	0	0.00	31	0	0	n/a	n/a	n/a	
**PACIFIC**	26	47	0	0	0	47	0.90	5	0.10	21	5	0	0.19	0.17	−0.11	
**UNITED KINGDOM**	32	32	32	0	0	64	1.00	0	0.00	32	0	0	n/a	n/a	n/a	

H_obs_ = observed heterozygosity; H_exp_ = expected heterozygosity assuming HWE; H_I_  = mean observed heterozygosity within subpopulations; H_S_  = mean expected heterozygosity within subpopulations; F_IS_ = (H_S_−H_I_)/H_S_; F_ST_ = (H_T_−H_S_)/H_T_; F_IT_ = (H_T_−H_I_)/H_T_; 1 = Significant departure from Hardy Weinberg equilibrium.

The 403 animals sequenced in this study harbored polymorphisms at seven different positions within a 159 bp stretch of NF-κB (Table 2; Figure S4). Five of these polymorphic positions reside in intronic regions (#'s 1–4, 7; Figure 2C). One of the two exonic polymorphisms is silent (#5), and the other is the Cys/Ser coding polymorphism.

**Table 2:** Different Nv-*nfkb* alleles and their allele frequencies.

SNP#1234567		
Position in alignment	6	15	33	36	51	64	158	n	Freq.
Allele C1	G	G	A	G	G	T	C	618	0.77
Allele C2	A	G	A	G	G	T	C	47	0.06
Allele C3	A	G	A	G	G	T	G	18	0.02
Allele C4	G	G	A	G	G	T	G	6	0.01
Allele S1	G	T	T	T	A	A	G	117	0.15

**Figure 4:**
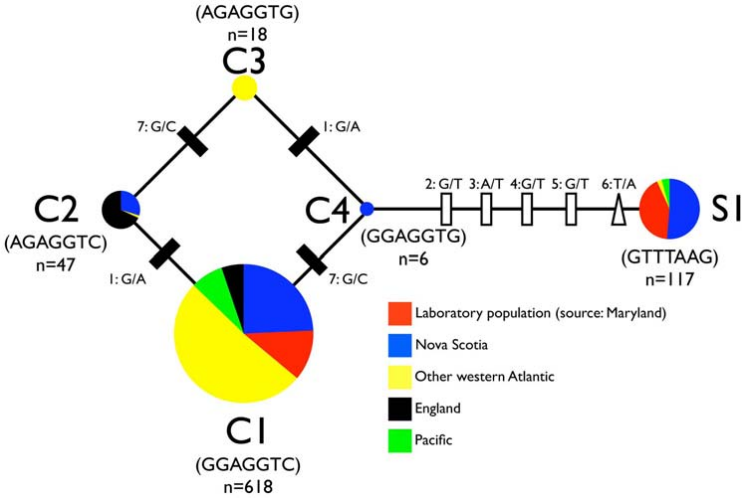
Haplotype tree depicting the relative abundances and mutational distances between the five *nfkb* variants. The evolution of the 5 *Nematostella nfkb* variants can be explained by two mutation events at SNP1, two events at SNP7, and five separate events at SNPs 2–6. Circles indicate individual alleles. The overall abundance of each allele is proportional to circle area. Colored wedges depict the fraction of each allele that comes from a given geographic region. Silent mutations are indicated by rectangles. The missense mutation underlying the Cys/Ser polymorphism in the DNA recognition loop is indicated by a triangle.

Together, the seven polymorphic positions define five distinct alleles, four alleles that encode Cys at position 67 (C1-C4) and one allele that encodes Ser (Table 2; Figure S5). Two of the five alleles were geographically widespread. The most common allele (C1) was recovered from every estuary sampled, and exhibited a global frequency of 77% (Tables 1, 2). The second most abundant allele (S1) was recovered from eight different estuaries in Nova Scotia, Massachusetts and California. The global frequency of the S1 allele was 15%. The remaining three alleles (C2-C4) are slight variants of C1 that differ at one or both of the flanking SNP positions (positions 1 and 7; Table 2; Figure 4), both of which reside in introns. These alleles have a more limited distribution, with allele C3 occurring only in Baruch, South Carolina, allele C4 occurring only in Nova Scotia, and allele C2 occurring only in Nova Scotia and England (Table 1; Figure 4).

### The two major Nv-NF-κB alleles show variable geographic distribution

**Figure 5:**
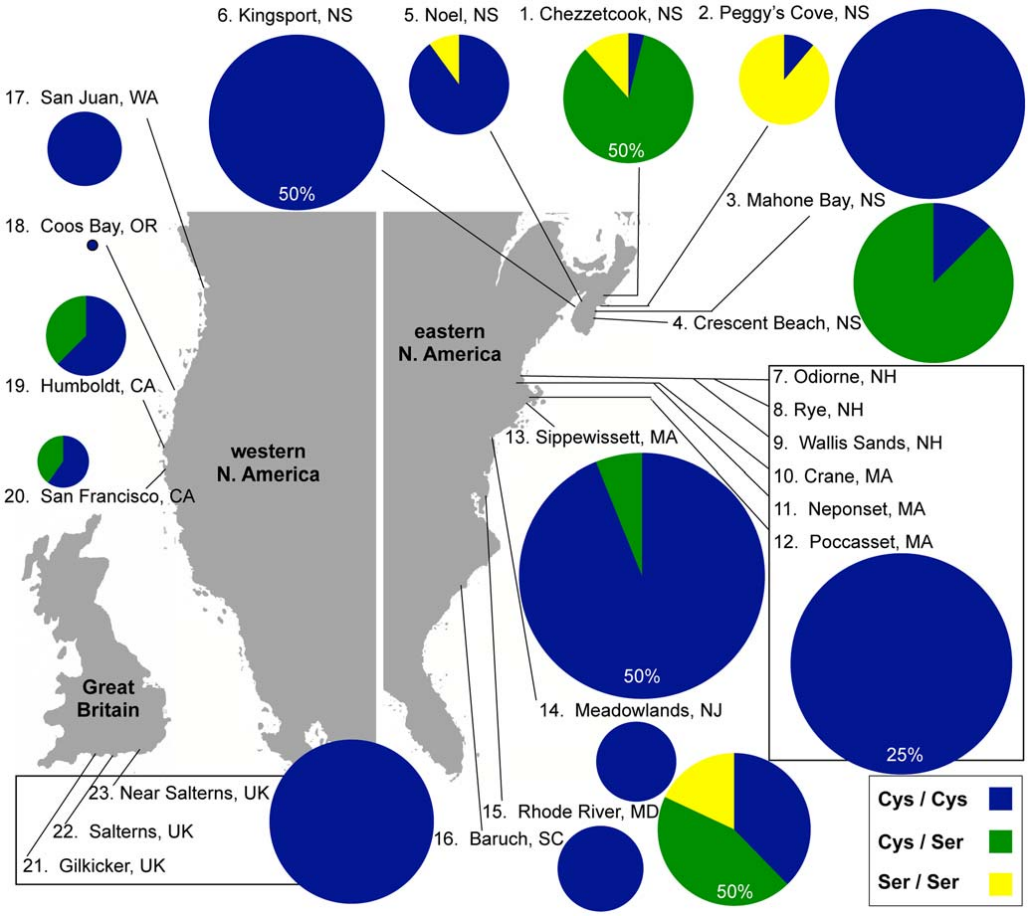
Geographical distribution of Cys (C1-C4) and Ser NF-κB genotypes. Pie graphs display the frequency of each genotype within the indicated populations (see Table 1 for details). The area of each graph reflects the relative sample size from that locale; for the large samples obtained at Chezzetcook (population 1), Kingsport (6), Sippewisset (13), and Rhode River (15), and the samples pooled from northern New England (7–12), the pie graphs are depicted at 50% or 25% of their relative size (as indicated on each graph). Populations 7–12 were grouped and populations 21–23 were grouped because the individual sites within each group are in close geographical proximity, and the groups exhibit no genotypic variability. The most abundant genotype, Cys/Cys, was found in every estuary sampled. With the exception of a few Ser/Ser individuals found in the lab population derived from Rhode River, Maryland, Ser/Ser homozygotes are restricted to Nova Scotia.

Because the differences among the four Cys alleles do not affect the protein sequence and are therefore unlikely to have fitness consequences, we pooled all of the Cys alleles and then evaluated the geographic distribution of Cys versus Ser alleles and genotypes (Table 1; Table 3; Figure 5). Of the 403 animals we genotyped, we identified 309 Cys/Cys homozygotes, 23 Ser/Ser homozygotes, and 71 heterozygotes (Table 1). The global F_ST_ value (0.56) indicates a high degree of genetic differentiation among subpopulations for this polymorphism (far fewer heterozygotes were found than expected if there were random mating among all individuals), which is consistent with previous population genetic studies on this species [27], [28]. However, the F_IT_ value (−0.19) is negative (there were more heterozygotes than expected within individual subpopulations). The excess of heterozygotes is particularly pronounced at Crescent Beach and Mahone Bay in Nova Scotia, and in the two California sites. The only wild populations that exhibited a deficit of heterozygotes were in Nova Scotia (Noel and Peggy's Cove). In Noel, the only Ser alleles identified were recovered from a single homozygous individual. In Peggy's Cove, eight of nine individuals were Ser/Ser homozygotes, and the remaining individual was Cys/Cys. The Peggy's Cove site is noteworthy for having by far the greatest excess of Ser/Ser homozygotes. In the laboratory population, which was originally collected from Rhode River, Maryland but has been reproducing under laboratory conditions for several years, all three genotypes were present at their expected frequencies under Hardy-Weinberg equilibrium.

**Table 3:** Distinct Nv-*nfkb* genotypes identified and their frequency of occurrence.

Genotype at SNP #6	Alleles	N	Frequency
**Cys/Cys**	C1/C1	244	0.605
	C1/C2	47	0.117
	C1/C3	14	0.035
	C1/C4	2	0.005
	C3/C3	2	0.005
**Cys/Ser**	C1/S1	67	0.166
	C4/S1	4	0.010
**Ser/Ser**	S1/S1	23	0.057

### Proteins encoded by the two alleles differ in DNA-binding and transactivation abilities

**Figure 6:**
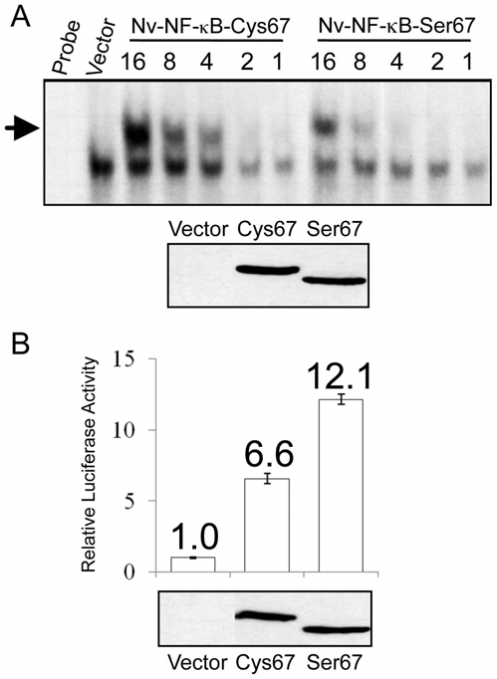
Comparison of the DNA-binding and transactivation properties of the proteins encoded by the two Nv-NF-κB alleles. (A) An EMSA was performed using a radioactive κB-site probe and A293 cell extracts containing approximately equal amounts of each indicated FLAG-Nv-NF-κB protein (top panel). Two-fold dilutions of the equalized extracts spanning a 16-fold concentration range were used in the EMSA. The position of the NF-κB-DNA complex is indicated by the arrow. Anti-FLAG Western blotting (bottom panel) was performed to verify that the amounts of the two proteins were roughly equal. Probe, probe alone; Vector, an extract from pcDNA empty vector-transfected cells containing an amount of protein equal to the “16” lane of Nv-NF-κB-S67. (B) A reporter gene assay using a multimerized κB-site luciferase reporter gene was performed in A293 cells as described in Materials and Methods. Cells were co-transfected with expression plasmids either containing no insert (Vector) or the indicated Nv-NF-κB proteins. Values are relative to the normalized luciferase activity seen with the Vector (1.0). Values are the averages of three experiments, each performed with triplicate samples. An anti-FLAG Western blot of normalized extracts used in the reporter gene assay, to confirm approximately equal expression of the two Nv-NF-κB proteins (bottom panel).

To determine whether there are differences in the activities of the proteins encoded by the two Nv-NF-κB alleles, we first subcloned their complete coding sequences into a mammalian cell expression vector (pcDNA) such that each protein would have an epitope tag (FLAG) at its N terminus. Transfection of A293 human cells with these vectors resulted in expression of the appropriately sized proteins, as determined by anti-FLAG Western blotting. Of note, the protein encoded by the Cys67 allele migrates slightly slower than the Ser67 protein on SDS-polyacrylamide gels (Figure 6A). We next used extracts of A293-transfected cells to compare the DNA-binding activities of the two Nv-NF-κB proteins in an electrophoretic mobility shift assay. Nv-NF-κB-Cys bound the κB-site probe approximately four times better than Nv-NF-κB-Ser (Figure 6A). On the other hand, in a κB-site reporter gene assay, Nv-NF-κB-Ser activated transcription approximately two times more strongly than Nv-NF-κB-Cys (Figure 6B). These results demonstrate that the proteins encoded by the two Nv-NF-κB alleles have distinct activities.

**Figure 7:**
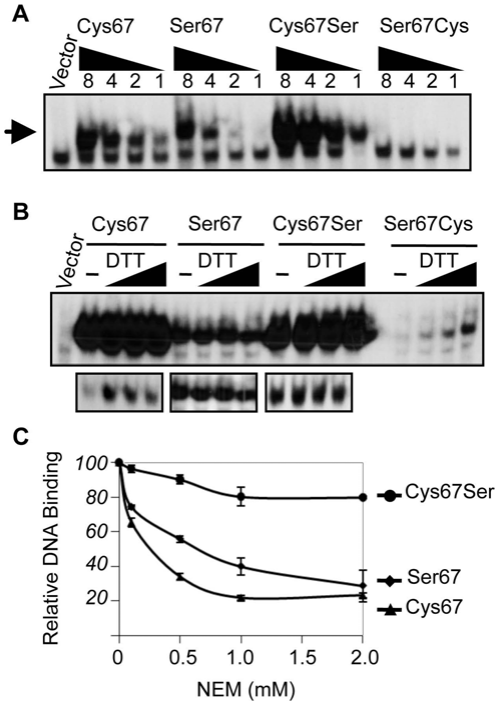
Effect of mutations at residue 67 on the DNA-binding activity of the two Nv-NF-κB proteins. (A) An EMSA was performed using a radioactive κB-site probe and A293 cell extracts containing equal amounts of each indicated FLAG-Nv-NF-κB protein (as determined by anti-FLAG Western blotting). As in Figure 6A, two-fold dilutions of the equalized extracts were used in the EMSA. The position of the NF-κB-DNA complex is indicated by the arrow. Probe, probe alone. (B) An EMSA was performed using extracts from A293 cells as in (A), except that the extracts were supplemented with increasing concentrations (0, 1, 5, 25 mM) of dithiothreitol (DTT) for 18 h prior to adding the radioactive probe to the samples. The lower panels show lighter exposures of the top panel, so that the relative intensities of DNA binding at the different concentrations of DTT can be visualized. (C) An EMSA was performed using extracts from A293 cells transfected with plasmids for expression of the indicated Nv-NF-κB proteins. However, the extracts were incubated with the indicated concentrations of N-ethylmaleimide (NEM) for 30 min prior to adding the radioactive κB-site probe. Values for each protein designate the relative the amount of DNA-binding activity at a given concentration of NEM as compared to the activity in the absence of NEM. Values (with standard error) are the averages of three separate experiments.

To determine whether the Cys/Ser polymorphism at residue 67 is entirely responsible for the different DNA-binding activities of the two Nv-NF-κB proteins, we created cDNAs encoding the relevant single amino acid changes at position 67 (i.e., Nv-NF-κBC67S and Nv-NF-κBS67C). As shown in Figure 7A, the Nv-NF-κBC67S mutant bound DNA more strongly than the parental Nv-NF-κBCys protein, whereas the Nv-NF-κBS67C mutant bound DNA more weakly than the full Nv-NF-κBSer protein. Thus, Ser at residue 67 enhances DNA binding by Nv-NF-κB, regardless of the other amino acid differences between the two NF-κB variants. Nevertheless, the native Nv-NF-κB-Ser protein binds DNA more weakly than the native Nv-NF-κB-Cys protein. These results indicate that even though the amino acid (Cys vs. Ser) at residue 67 influences the ability of Nv-NF-κB to bind DNA, other amino acid changes also contribute to the differences in DNA binding between the two Nv-NF-κB proteins.

Because the homologous Cys residue has been shown to influence the susceptibility of vertebrate NF-κB proteins to both redox regulation and thiol-reactive compounds [12]–[16], [18]–[23], we next compared the effects of the reducing agent dithiothreitol (DTT) and the thiol-reactive compound N-ethylmaleimide (NEM) on the two native Nv-NF-κB proteins, as well as the relevant site-directed mutants. The addition of DTT to protein extracts increased the DNA-binding ability of the NF-κB-Cys and NF-κBS67C, but did not significantly affect the DNA-binding ability of either protein with Ser at residue 67 (Figure 7B). Conversely, the addition of NEM dramatically decreased DNA binding by NF-κB-Cys but had little effect on the NF-κBC67S mutant (Figure 7C). NEM also inhibited DNA binding by the full NF-κB-Ser protein to a lesser extent than the NF-κB-Cys protein, but the NF-κB-Ser protein was more susceptible to inhibition by NEM than the NF-κBC67S mutant (Figure 7C). These results indicate that the two NF-κB proteins are affected differently by thiol-reactive compounds, but that the effects of such compounds are not entirely determined by the presence of Cys or Ser at position 6 in the DNA recognition loop.

## Discussion

### Evolution and distribution of the NF-κB allele containing Ser at residue 67

In nearly all NF-κB/Rel proteins, representing many billions of years of cumulative evolution, Cys is conserved at position 6 of the DNA recognition loop. However, in the Relish proteins of several insects, Ser is conserved at this same position. Therefore, on a macroevolutionary scale, we have evidence that either Cys or Ser can be adaptive in a particular genic/taxonomic context. Presumably, at some time in the past, Ser replaced Cys in the DNA recognition loop of an ancestral Relish protein, and this change was adaptive. *Nematostella* presents an opportunity to investigate the conditions under which such a swap might be adaptive, as it is currently the only species known to harbor a Cys/Ser polymorphism at this critical position within the DNA recognition loop of an RHD protein.

Our population genetic evidence suggests that in *Nematostella*, at least in some sites, the Ser variant of NF-κB confers a fitness advantage. First, the Ser allele has spread widely throughout the species' global range, and in some populations it is the predominant allele. The Ser allele was recovered in six western Atlantic estuaries that are thought to be part of *Nematostella's* native range [28]—four in Nova Scotia, one in Massachusetts, and one in Maryland. It was also recovered in two eastern Pacific coast estuaries where the animal is believed to have been recently introduced [28].

In four out of eight populations that possess both Cys and Ser alleles, there is a significant departure from HWE (Table 1), a result that might be attributable to selection. In some natural populations, Ser/Ser homozygotes are markedly underrepresented, while heterozygotes are markedly over-represented (e.g., Crescent Beach and Chezzetcook, Nova Scotia). At Peggy's Cove, Ser/Ser homozygotes are over-represented. In the laboratory population, both alleles are moderately abundant, and genotypic frequencies accord with expectations under Hardy-Weinberg equilibrium. This laboratory population was originally derived from Rhode River, Maryland and has been maintained for ∼20 years, probably in a state of relaxed selective pressures.

One cannot necessarily interpret departures from Hardy-Weinberg equilibrium as evidence of selection, given that demographic factors can influence population genetic structure. For example, previous studies have revealed pronounced population genetic structuring in *Nematostella* at local, regional, and global scales [27], [28]. These genetic breaks could reflect local adaptation, but they could also result from genetic drift and the animal's limited dispersal ability or from pervasive asexual reproduction. Indeed, evidence of clonal reproduction has been detected in several estuaries, and some natural populations are almost entirely clonal [27]. In fact, the results of this study on NF-κB alleles provide evidence that the *Nematostella* population of England is solely or predominantly clonal. All English animals sampled in our study (n = 32) are heterozygous at the NF-κB locus (C1/C2; Table 3). Our inability to recover any C1/C1 or C2/C2 homozygotes implies a lack of sexual recombination and supports the hypothesis that these populations are comprised of a single clonal lineage [28], [29]. The previous studies that investigated genetic diversity within English populations [28], [29] could not have detected this paucity of recombination because they used dominant markers (RAPDs and AFLPs).

### Possible Selective Advantages of the Derived Ser Allele

Based on the near universal presence of Cys at position 6 of the DNA recognition loop of NF-κB proteins and the character state reconstruction shown in Figure 3, we conclude that the presence of Cys is the primitive condition for NF-κB in *Nematostella*. However, the Ser variant is widespread and reasonably abundant in wild populations, particularly in Nova Scotia where the Ser allele constitutes 26% of all alleles sampled from six different estuaries (Table 1). The Ser allele could be favored in certain estuarine environments where the anemones are exposed to high levels of peroxides and other reactive oxygen species or to alkylating agents. The sulfhydryl group of Cys can react readily with such compounds—such as NEM (Figure 7), epoxyquinoids [13], and sesquiterpene lactones [11]—which would then inhibit the Cys variant from binding to DNA targets, whereas the hydroxyl group of the Ser variant would make Nv-NF-κB less susceptible to the effects of such compounds. Indeed, *Nematostella* inhabits estuarine environments including tidal creeks and isolated high marsh pools where the concentration of peroxides and thiol-reactive compounds can vary considerably over fine temporal and spatial scales [30]. For example, differences in H_2_O_2_ concentration spanning three orders of magnitude have been reported within a single marsh, and the concentration of H_2_O_2_ that has been measured in salt marsh habitats (up to ∼4.5 nM) is within the range that has been shown to reduce invertebrate metabolic rates [31], [32].

At the molecular level, there are functional differences between the Nv-NF-κB proteins encoded by the two alleles. Namely, the Cys variant of *Nematostella* NF-κB has a more avid binding affinity for a κB site from the promoter of the Nv-IκB gene (Figure 6A); however, the Cys variant has a lesser ability to activate a multimeric κB-site promoter in transient transfection assays in human A293 cells (Figure 6B). Although increased transactivation ability coupled with decreased DNA binding by the Ser variant would seem counterintuitive, rapid turnover of transcription factors on DNA has been coupled with increased transactivation [33].

In other NF-κB proteins, such as c-Rel and RelA, site-directed mutations that convert the natural Cys residue (at the position homologous to Cys67 of Nv-NF-κB) to Ser increase the affinity of the protein for a κB site [12]–[15]. Indeed, we also find that a Cys-to-Ser mutation at residue 67 increases the DNA-binding activity of the full Cys allele protein, while the corresponding Ser-to-Cys mutation at residue 67 decreases the DNA-binding activity of the full Ser allele protein (Figure 7A). Thus, the low DNA-binding activity of the full Ser allele NF-κB protein as compared to the full Cys allele protein must be due to other differences between the two proteins, i.e., at one or more of the other five differences between the two proteins in the RHD. The simplest model to explain the differences between the two Nv-NF-κB proteins is that one or more of these other amino acid differences confers relatively stronger DNA-binding activity on the Cys variant. Using the coral sequence to root the branch connecting the two *Nematostella* NF-κB variants (Figure 3), we have identified at least one additional substitution that appears to have occurred along the line leading to Nv-NF-κB-Cys (residue 218). Four additional substitutions appear to have occurred along the line leading to Nv-NF-κB-Ser (residues 67, 216, 226, 269). Given that the Ser allele derives from an ancestral Cys allele, one or more of these four substitutions that occur along the Ser branch are likely to cause reduced DNA binding. These residues might directly contact DNA, they could alter the ability of the DNA recognition loop to bind DNA, or they might affect dimerization (which is required for DNA binding). That the two Nv-NF-κB proteins show a difference in migration on SDS-polyacrylamide gels (Figure 5A) suggests that amino acid differences between the two proteins cause them to have local structural or charge differences as well.

Several previous studies have investigated the functional consequences of a Cys-to-Ser mutation at position 6 of the DNA recognition loop of NF-κB. This experimentally generated mutation, in human RHD proteins [11]–[14] or sea anemone NF-κB-Cys (this study), has pronounced effects on activity. This may explain why strong stabilizing selection has preserved Cys at this position in numerous animal lineages over many billions of years of cumulative evolution (Figure 1; Figure S1). However, the NF-κB polymorphism of *Nematostella* provides an opportunity to investigate how and why the Cys-to-Ser mutation evolved in nature. For example, because we were able to directly compare the native alleles to the site-directed mutants, we were able to infer that changes at other sites must be altering the functional impact and possibly the selective advantage of the Cys-to-Ser mutation. Importantly, the coding mutations that occur along the branch leading to Nv-NF-κB-Ser (Figure 3) appear to counteract some of the effects of the Cys-to-Ser substitution. In the EMSAs with and without the addition of NEM, the effects of the Cys-to-Ser mutation were more pronounced than the difference we observed between the native Cys variant and the native Ser variant (Figure 7A, C). The effects of these co-evolving sites in the protein may help to explain how *Nematostella* overcame the selective disadvantage of having Ser at position 6 of the DNA recognition loop in NF-κB.

Given the data presented here, we propose the following testable hypotheses: (1) that the Ser allele is favored in estuarine environments that frequently expose the animal to high levels of peroxides and/or thiol-reactive compounds, (2) that the Cys allele is favored in estuarine environments that rarely expose the animal to high levels of peroxides and thiol-reactive compounds, and (3) that the presence of Ser at position 6 of the DNA recognition loop is tolerated only in combination with other co-evolved changes.

### Conclusions

*Nematostella vectensis* is currently the only species known to harbor a Cys/Ser polymorphism at position six in the DNA recognition loop of NF-κB. The Ser variant of the protein is derived from an ancestral Cys variant, it appears to have arisen only once, and it has become broadly established throughout the animal's extensive geographic range. As shown by site-directed mutagenesis studies, the presence of Ser at this position of *Nematostella* NF-κB confers higher DNA-binding affinity and greater resistance to thiol-reactive compounds. However, the full Nv-NF-κB proteins encoded by the Cys and Ser alleles differ at nine other amino acid positions, and paradoxically, the native Cys variant binds DNA with greater affinity than the native Ser variant. Despite its lower DNA-binding affinity, the native Ser variant is a more potent activator of transcription. These studies demonstrate functional differences between the proteins encoded by the two alleles, and given that the derived Ser allele has become geographically widespread, it suggests that the Ser allele can be favored by natural selection, perhaps under conditions of oxidative or alkylating stress. Thus, this polymorphism may represent an adaptation of an environmental conformer living in a hypervariable environment.

## Materials and Methods

### Animal collection and maintenance and DNA extraction

Animals were collected from salt marsh habitats by sieving sediment obtained from isolated pools and tidal creeks through a ∼1 mm^2^ nylon screen. The anemones were transported to Boston University in water obtained at the collection sites, and subsequently, they were acclimated to ∼11 ppt artificial salt water (Instant Ocean^®^). Live, field-collected specimens were also kindly provided by local researchers for some populations (Coos Bay, Oregon by S. Arellano; San Juan Island, Washington by T. McGovern; and all English populations by K. Kaltas). In the lab, the anemones were fed freshly hatched *Artemia* nauplii until they attained a size of at least 10 mm. At this point, the animals were starved for 7 to 10 days to minimize the possibility of contaminating *Artemia* tissue in the DNA extraction. DNA was extracted using DNeasy kits (Qiagen, Valencia, CA) from either whole anemones or from the aboral section of individuals that had been bisected with a razor blade or scalpel.

### Characterization of the Nv-NF-κB polymorphism and reconstruction of allele evolution

A fragment of the *Nematostella* NF-κB gene (gi|156079903|gb|EU092640.1) was amplified from genomic DNA using species-specific primers (forward primer: 5′-CACMGAGCCCTACCTAGAAA-3′ where M = A/C; reverse primer: 5′-TCGCTGTCATGTGTTGATCC-3′). These primers were based on available ESTs and were designed to flank the nucleotide position responsible for the Cys/Ser polymorphism. Forty cycles of PCR were performed using the following thermal cycling profile: 94°C for 30 s, 55°C for 30 s, and 72°C for 60 s. The amplified 753-bp product comprises 293 base pairs of exonic sequence (from exons 2, 3, and 4) and 460 base pairs of intronic sequence (from introns 2 and 3; Figure 2B). PCR samples were electrophoresed on a 1% agarose gel. Amplified DNA was gel-purified using the QIAquick Gel Extraction kit (Qiagen, Valencia, CA) and sequenced at Macrogen (Seoul, South Korea) using the reverse primer. As a control, five DNA samples were analyzed twice, i.e., the NF-κB fragment was independently amplified and sequenced twice from the same DNA sample; in all five cases, the replicate samples yielded identical sequences.

Polymorphic nucleotide positions were identified from sequence chromatograms. The sequencing chromatograms were visualized using FinchTV [34]. The accuracy of nucleotide assignments was verified by eye for every position. Any nucleotide position in the sequencing chromatogram exhibiting a minor peak equal to or greater than a quarter of the height of the major peak was considered potentially polymorphic and was labeled using degenerate IUPAC codes.

Haplotypes were deduced from the sequenced PCR products (which combine maternal and paternal alleles from individual animals) using Bayesian inference (Phase, v.2.1; [35]). Haplotype reconstruction was based on a 159-bp stretch of sequence that includes numerous variable loci. This region was aligned for 403 animals from 23 geographically distinct estuaries (Figure S4). The alignment was then culled to seven loci harboring suspected polymorphisms (positions 6, 15 33, 36, 51, 64 and 158). Each of these positions was found to occur in three states (as either of two different nucleotides or as the corresponding degenerate nucleotide; 6: A, G, or R; 15: G, T, or K; 33: A, T, or W; 36: G, T, or K; 51: A, G, or R; 64: A, T, or W; 159: C, G, or S). At position 104, the large majority of individuals harbored a cytosine, but some individuals were scored as being ambiguous (either cytosine or thymidine). As no individual was scored unambiguously as a thymidine, we could not rule out the possibility that the ambiguity was due to a sequencing artifact, and this position was excluded from the haplotype reconstruction.

Five distinct alleles were identified by Phase, each achieving a probability score of 96.9% or greater (Figure S5). In 96.5% of individuals (389/403), the allele pairs were identified with no ambiguity. Among the remaining 3.5% of individuals, the best allele pairs were assigned a probability of 96.9–97.0%. As four of the alleles encode a Cys at position 6 of the DNA recognition loop, these alleles were labeled C1-C4. The remaining allele, which encodes a Ser at the corresponding site, was labeled S1.

### Statistical tests for departures from Hardy-Weinberg equilibrium

For all 403 animals included in the haplotype reconstruction, the geographic distribution of the three possible genotypes at SNP #6 (Cys/Cys, Cys/Ser, or Ser/Ser) was used to calculate F-statistics (F_IS_, F_ST_, and F_ST_; see Table 1). Individual estuaries were treated as subpopulations. Expected and observed heterozygosities were compared for each subpopulation [36], [37].

The observed genotypic proportions were compared to expected genotypic proportions within each population and across all populations that harbored both the Cys and Ser alleles. The expected proportion of each genotype (Cys/Cys, Cys/Ser, and Ser/Ser) was calculated under the assumption of Hardy-Weinberg equilibrium using observed allele frequencies. Chi-square tests were then used to identify populations that departed significantly from Hardy-Weinberg equilibrium and genotypes that were significantly overrepresented or underrepresented (α = 0.05). The individuals representing Rhode River, Maryland were not genotyped immediately after being collected from the field. Rather, the animals evaluated from this locale were derived from a laboratory stock that had been allowed to freely reproduce (sexually and asexually) under laboratory conditions since being collected in the field approximately 15 years ago. As the laboratory setting does not closely mimic field conditions, this population was excluded from the across-population analyses of genotype frequencies.

### Cloning and expression of Nv-NF-κB cDNAs

Total mRNA was isolated from starved adult animals, and cDNA was prepared by reverse transcription. To amplify the full-length coding region of the Nv-NF-κB cDNAs, forward (5′CGGAATTCCGTCCTAGTGGTGTATCAAGTGCAG3; EcoRI site underlined') and reverse (5′TCGGTCGACCGCGAAAACCCAATTGGAG3′; SalI site) primers were used in the PCR, and the cDNAs were subcloned into the corresponding sites of pcDNA3.1 (+). The full coding region was sequenced. To subclone codons 3–440 of each cDNA in-frame into pcDNA-FLAG, the Nv-NF-κB cDNAs were re-amplified using primers containing restriction sites (5′CGGAATTCGACACAGTCTGAACAGCAAGTG3′ and 5′CGGAATTCCGAAAACCCAATTGAATGGAAG3′; EcoRI site) and were then subcloned into pcDNA-FLAG digested with EcoRI. Additional details about primers and subcloning methods used to create mutants S67C and C67S can be obtained at www.nf-kb.org.

### Electrophoretic mobility shift and reporter gene assays

A293 cells were transfected with 5 µg of pcDNA or the indicated pcDNA-FLAG-Nv-NF-κB expression vector using polyethylenimine (Polysciences, Inc., Washington, PA) as described previously [38]. Two days later, extracts were prepared using AT lysis buffer, and they were subjected to anti-FLAG Western blotting, or an EMSA was performed as described previously [38]. When DTT or NEM was included (Figure 6), extracts were prepared in AT lysis buffer that lacked DTT. The κB-site probe (5′-TCGAGAGGTCGGGGAATCCCCCCCCG-3′; κB site underlined) used for the EMSAs is from the upstream region of the Nv-IκB gene [6]. Dried EMSA gels were subjected to autoradiography at −80°C.

Reporter gene assays were performed in A293 cells essentially as described [38]. Cells were transfected with 1 µg of pcDNA-FLAG expression plasmid, 0.5 µg of a 3X κB-site luciferase reporter plasmid, and 0.5 µg of plasmid pGK-βgal for transfection normalization.

## Supporting Information

Figure S1 Partial alignment of the Rel Homology Domain of representative RHD proteins. The DNA recognition loop is shaded in gray. The sixth position in the DNA recognition loop is highlighted in blue (if Cys), red (if Thr), or purple (if Ser). Nematostella sequences are shown in bold type.
(0.09 MB PDF)

Figure S2 Alignment of alleles identified in this study. The complete coding sequences of Nv-NF-κB were determined for both Cys and Ser alleles derived from animals in our laboratory population. In addition, a 753-nucleotide region of the Nv-NF-κB gene spanning all or part of three exons and two introns (Figure 2) was amplified and sequenced for 403 individuals from 23 populations (Table 1). Seven variable positions were identified within a stretch of 153 nucleotides (1–7), allowing us to define five distinct alleles (C1-C4, S1). Protein-coding polymorphisms are highlighted in green. Silent polymorphisms are highlighted in red. The predicted amino acid sequence is presented beneath the nucleotide sequences of the complete coding sequences.
(0.06 MB PDF)

Figure S3 Alignment of Nematostella proteins against partial coral NF-κB protein. A partial coding sequence for the NF-κB gene of Acropora millepora was recently recovered in a transcriptome sequencing project [7]. The predicted coral protein (AmNFκB) was aligned against the Nematostella proteins using a web implementation of ClustalW2 [39]. The positions that are polymorphic in Nematostella are indicated by bold red type. Invariant positions are indicated by asterisks.
(0.06 MB PDF)

Figure S4 Alignment of 159-nt region of Nv-NF-κB from 403 individual animals. Where a position in the sequence chromatogram harbored peaks for two different nucleotides, the appropriate IUPAC code is used (K = G or T; R = A or G; S = C or G; W = A or T).
(0.81 MB PDF)

Figure S5 Individual animals sequenced in the population genetic study. For each individual, the table lists the collection site, the sequences of the individual's two NF-κB alleles at the seven variable positions in the alignment (Allele 1 and Allele 2 SNPs), the posterior probability that these “best alleles” are the true alleles (Prob.), the name of each allele (Allele 1, Allele 2), and the genotype.
(0.14 MB PDF)
