# Supplementary material for: Two Alleles of NF-κB in the Sea Anemone Nematostella vectensis Are Widely Dispersed in Nature and Encode Proteins with Distinct Activities
Source: PLoS One. 2009 Oct 6;4(10):e7311. doi: 10.1371/journal.pone.0007311 (PMC2751831; doi:10.1371/journal.pone.0007311)
Supplement: Figure S1 — Partial alignment of the Rel Homology Domain of representative RHD proteins. The DNA recognition loop is shaded in gray. The sixth position in the DNA recognition loop is highlighted in blue (if Cys), red (if Thr), or purple (if Ser). Nematostella sequences are shown in bold type. (0.09 MB PDF) [file pone.0007311.s001.pdf]

Supplemental Figure 1. Partial alignment of the DNA binding region in select members of the RHD superfamily.

**NFkB (*Nematostella*)**	**[ABU48530]**	**50**	**LEILEQPKPRGFRFRYPSEG**	**69**
		**50**	**LEILEQPKPRGFRFRYPCEG**	**69**
**NFκB**				
NFκB (*Amphimedon*)	[ABW76682.1]	48	LEIVEQPKSRGFRFRYDCEG	67
NFκB (*Ciona*)	[NP_001071772.1]	26	LEIIENPKSRGFRFRYTCEG	45
NFκB (*Strongylocentrotus*)	[NP_999819.1]	62	LKILEQPRQRGFRFRYGCEG	81
NFκB (*Pinctada*)	[ABL63469.1]	102	TEIMEQPKQRGLRFRYECEG	121
NFκB (*Euprymna*)	[AAY27981.1]	48	VVITEQPKSRGFRFRYQCEG	65
NFκB1 (*Homo*)	[NM_003998.2]	45	LQILEQPKQRGFRFRYVCEG	64
NFκB1 (*Macaca*)	[XP_001109277.1]	45	LQILEQPKQRGFRFRYVCEG	64
NFκB1 (*Mus*)	[EDL12142.1]	42	LQILEQPKQRGFRFRYVCEG	61
NFκB1 (*Rattus*)	[EDL82271.1]	42	LQILEQPKQRGFRFRYVCEG	61
NFκB1 (*Equus*)	[XM_001915438.1]	43	LQILEQPKQRGFRFRYVCEG	62
NFκB1 (*Bos*)	[AAI53233]	44	LQILEQPKQRGFRFRYVCEG	63
NFκB1 (*Sus*)	[ABU63584.1]	44	LQILEQPKQRGFRFRYVCEG	63
NFκB1 (*Canis*)	[NP_001003344.1]	44	LQILEQPKQRGFRFRYVCEG	63
NFκB1 (*Monodelphis*)	[XP_001368747.1]	28	LQILEQPKQRGFRFRYVCEG	47
NFκB1 (*Gallus*)	[AAB58343.1]	70	LQIIEQPKQRGFRFRYVCEG	89
NFκB1 (*Danio*)	[AAY67903.1]	49	LQITEQPKQRGFRFRYGCEG	68
NFκB2 (*Homo*)	[EAW49703.1]	40	LVIVEQPKQRGFRFRYGCEG	59
NFκB2 (*Macaca*)	[XP_001104566.1]	40	LVIVEQPKQRGFRFRYGCEG	59
NFκB2 (*Mus*)	[AAY44753.1]	40	LVIVEQPKQRGFRFRYGCEG	59
NFκB2 (*Rattus*)	[NP_001008350.1]	40	LVIVEQPKQRGFRFRYGCEG	59
NFκB2 (*Equus*)	[XP_001916453.1]	40	LVIVEQPKQRGFRFRYGCEG	59
NFκB2 (*Bos*)	[NP_001095571.1]	40	LVIVEQPKQRGFRFRYGCEG	59
NFκB2 (*Sus*)	[XP_001925739.1]	40	LVIVEQPKQRGFRFRYGCEG	59
NFκB2 (*Canis*)	[XP_543988.2]	40	LVIVEQPKQRGFRFRYGCEG	59
NFκB2 (*Monodelphis*)	[XP_001379024.1]	44	LVIVEQPKQRGFRFRYGCEG	63
NFκB2 (*Gallus*)	[P98150.1]	39	LVIIEQPKQRGFRFRYVCEG	58
NFκB2 (*Xenopus*)	[AAH76882.1]	45	LSIIEQPKQRGFRFRYVCEG	64
NFκB2 (*Danio*)	[NP_001001840.2]	55	IQIIEEPKQRGFRFRYECEG	74
**REL**				
Rel2 (*Crassostrea*)	[AAK72691.1]	85	VEIVEQPKQRGLRFRYECEG	104
RelA (*Ciona*)	[AAV80379.1]	7	LEIVEQPKQRGMRFRYECEG	26
Rel (*Halocynthia*)	[BAB47173.1]	15	LVITEQPKQRGMRFRYECEG	34
Rel (*Homo*)	[CAA52954.1]	10	IEIIEQPRQRGMRFRYKCEG	29
Rel (*Pan*)	[ABM54265.1]	8	IEIIEQPRQRGMRFRYKCEG	27
Rel (*Mus*)	[CAA33843.1]	10	VEIIEQPRQRGMRFRYKCEG	29
Rel (*Gallus*)	[XP_419277.2]	9	IEIFEQPRQRGMRFRYKCEG	28
Rel (*Danio*)	[AAO26402.1]	20	VQIFEQPKQRGMRFRYKCEG	39
RelA (*Homo*)	[BAG64344.1]	21	VEIIEQPKQRGMRFRYKCEG	40
RelA (*Pan*)	[ABM91918.1]	21	VEIIEQPKQRGMRFRYKCEG	40
RelA (*Mus*)	[AAY44745.1]	21	VEIIEQPKQRGMRFRYKCEG	40
RelA (*Equus*)	[XP_001490867.2]	21	VEIIEQPKQRGMRFRYKCEG	40
Rel? (*Monodelphis*)	[XP_001379658.1]	15	VEIIEQPKQRGMRFRYKCEG	34
RelA-a (*Xenopus*)	[AAH70711.1]	19	VEIIEQPKQRGMRFRYKCEG	38
RelA (*Danio*)	[AAI62910.1]	20	VEIIEQPKSRGMRFRYKCEG	39

Supplemental Figure 1. (continued)

RelB (*Homo*)	[BAF83283.1]	127	LVITEQPKQRGMRFRYECEG	146
RelB (*Pan*)	[XP_512742.2]	127	LVITEQPKQRGMRFRYECEG	146
RelB (*Mus*)	[EDL23169.1]	143	LVITEQPKQRGMRFRYECEG	162
RelB (*Equus*)	[XP_001917013.1]	145	LVISEQPKQRGMRFRYECEG	164
RelB-a (*Xenopus*)	[NP_001079335.1]	80	LNITEQPKQRGMRFRYQCEG	99
RelB (*Danio*)				
**DIF /DORSAL**				
Dif (*Drosophila*)	[AAM48404.1]	80	LRIVEEPTSNIIRFRYKCEG	99
Dorsal (*Drosophila*)	[AAT94434.1]	49	VKITEQPAGKALRFRYECEG	68
Dorsal (*Tribolium*)	[AAG22858.1]	65	VKIIEQPASKALRFRYECEG	83
Gambif1 (*Anopheles*)	[XP_310177.3]	50	VEITEQPHPKALRFRYECEG	69
**RELISH**				
Relish (*Drosophila*)	[AAF20134.1]	87	LRIVEQPVEK-FRFRYKSEM	105
Relish (Tribolium)	[XP_970894.1]	1	LRIIEQPIDR-FRFRYKSEM	79
Relish (*Aedes*)	[XP_001658517.1]	135	LTIVEQPVDK-FRFRYQSEM	153
**NFAT**				
**NFAT (*Nematostella*)**	**[XP_001632550.1]**	**519**	**LVLLEEPEEN-YRARYESEG**	**537**
NFAT (*Drosophila*)	[NP_001096974.1]	504	LEILSQPEQQ-HRARYQTEG	522
NFAT (*Ciona*)	[XP_002120942.1]	348	LSIVNQPEPQ-HRARYLTEG	366
NFAT (*Strongylocentrotus*)	[XP_001177692.1]	230	IHITVQPEKH-HRARYRTEG	248
NFAT1 (*Homo*)	[ACG55586.1]	415	LRIEVQPKSH-HRAHYETEG	433
NFAT1 (*Xenopus*)	[NP_001072740.1]	417	LRIEVQPKSH-HRAHYETEG	435
NFAT1 (*Danio*)	[NP_001038624.1]	369	LQIDVQPKSH-HRAHYETEG	387
NFAT1 (*Gallus*)	[XP_418906.2]	413	LRIEVQPKSH-HRAHYETEG	431
NFAT2 (*Homo*)	[ACG55599.1]	191	LRIEVQPKPH-HRAHYETEG	209
NFAT2 (*Gallus*)	[XP_417509.2]	410	LRIEVQPKPH-HRAHYETEG	428
NFAT2 (*Danio*)	[XP_700591.3]	337	LRIEAQPRQH-HRAHYETEG	355
NFAT3 (*Homo*)	[BAF83059.1]	438	LKIEVQPKTH-HRAHYETEG	451
NFAT3 (*Gallus*)	[XP_414078.2]	555	LKIEVQPKTH-HRAHYETEG	573
NFAT3 (*Danio*)	[XP_690273.3]	443	LKIEVQPKAH-HRAHYETEG	461
NFAT4 (*Homo*)	[ACG55669.1]	349	LRIEVQPRAH-HRAHYETEG	367
NFAT4 (*Danio*)	[XP_690183.2]	270	LRIEVQPRPH-HRAHYETEG	288
NFAT5 (*Homo*)	[NP_619727.2]	300	LKIVVQPETQ-HRARYLTEG	318
NFAT5 (*Monodelphis*)	[XP_001378219.1]	510	LKIVVQPETQ-HRARYLTEG	528
NFAT5 (*Gallus*)	[BAG70407.2]	206	LKIVVQPETQ-HRARYLTEG	224
NFAT5 (*Danio*)	[NP_956189.1]	268	LKILVQPETQ-HRARYLTEG	286
