# Supplementary material for: Two Alleles of NF-κB in the Sea Anemone Nematostella vectensis Are Widely Dispersed in Nature and Encode Proteins with Distinct Activities
Source: PLoS One. 2009 Oct 6;4(10):e7311. doi: 10.1371/journal.pone.0007311 (PMC2751831; doi:10.1371/journal.pone.0007311)
Supplement: Figure S2 — Alignment of alleles identified in this study. The complete coding sequences of Nv-NF-κB were determined for both Cys and Ser alleles derived from animals in our laboratory population. In addition, a 753-nucleotide region of the Nv-NF-κB gene spanning all or part of three exons and two introns (Figure 2) was amplified and sequenced for 403 individuals from 23 populations (Table 1). Seven variable positions were identified within a stretch of 153 nucleotides (1–7), allowing us to define five distinct alleles (C1-C4, S1). Protein-coding polymorphisms are highlighted in green. Silent polymorphisms are highlighted in red. The predicted amino acid sequence is presented beneath the nucleotide sequences of the complete coding sequences. (0.06 MB PDF) [file pone.0007311.s002.pdf]

Supplemental Figure 2. Alignment of NF-κB alleles.

```
                    10        20        30        40        50        60
NvNFkB_C67   ATGGCACAGTCTGAACAGCAAGTGGGTAGTGCTCTCACAGAAAGCATGTTAAATGAGATC
NvNFkB_S67   ATGGCACAGTCTGAACAGCAAGTGGGTAGTGCTCTCACAGAAAGCATGTTAAATGAGATC
              M  A  Q  S  E  Q  Q  V  G  S  A  L  T  E  S  M  L  N  E  I
                    70        80        90       100       110       120
NvNFkB_C67   ATTCAACCTGGTTATCTACCAGATATCAGTGCTCTGCATGTCCCATTAGGCACAAATGCA
NvNFkB_S67   ATTCGACCTGGTTATCTACCTGATATCAGTGCTCTGCATGTCCCATTAGGCACAAATGCA
              I Q/R P  G  Y  L  P  D  I  S  A  L  H  V  P  L  G  T  N  A
                                                                1
                   130       140       150       160       170       180
NvNFkB_C67   GAGGAGCCTTCTTACACCGAGCCCTACCTAGAAATTCTTGAACAGCCTAAACCGGCATGG
NvNFkB_S67   GAGGAGCCTTCTTACACCGAGCCCTACCTAGAAATTCTTGAACAGCCTAAACCGGCATGG
              E  E  P  S  Y  T  E  P  Y  L  E  I  L  E  Q  P  K  P [.....
Allele C1                                                         GCATGG
Allele C2                                                         GCATGG
Allele C3                                                         ACATGG
Allele C4                                                         ACATGG
Allele S1                                                         GCATGG
                 2                  3  4              5            6
                   190       200       210       220       230       240
NvNFkB_C67   AAAGAATGATTAATTGAATTAATTGTAGCGTGGTTTCCGGTTCCGATACCCATGCGAAGG
NvNFkB_S67   AAATAATGATTAATTGAATTATTTTTAGCGTGGTTTCCGATTCCGATACCCAAGTGAAGG
             .........intronic...........]R  G  F  R  F  R  Y  P C/S E  G
Allele C1    AAAGAATGATTAATTGAATTAATTGTAGCGTGGTTTCCGGTTCCGATACCCATGTGAAGG
Allele C2    AAAGAATGATTAATTGAATTAATTGTAGCGTGGTTTCCGGTTCCGATACCCATGTGAAGG
Allele C3    AAAGAATGATTAATTGAATTAATTGTAGCGTGGTTTCCGGTTCCGATACCCATGTGAAGG
Allele C4    AAAGAATGATTAATTGAATTAATTGTAGCGTGGTTTCCGGTTCCGATACCCATGTGAAGG
Allele S1    AAATAATGATTAATTGATTTATTTTTAGCGTGGTTTCCGATTCCGATACCCAAGTGAAGG
                   250       260       270       280       290       300
NvNFkB_C67   GCCTTCCCATGGGGGACTCCCTGGGCAGTTTTCGACTTCTAAAAGCAAGTCATACCCGTC
NvNFkB_S67   GCCTTCCCATGGGGGACTCCCTGGGCAGTTTTCGACTTCTAAAAGCAAGTCATACCCGTC
               P  S  H  G  G  L  P  G  Q  F  S  T  S  K  S  K  S  Y  P  S
Allele C1    GCCTTCCCATGGGGGACTCCCTGGGCAGTTTTCGACTTCTAAAAGCAAGTCATACCCGTC
Allele C2    GCCTTCCCATGGGGGACTCCCTGGGCAGTTTTCGACTTCTAAAAGCAAGTCATACCCGTC
Allele C3    GCCTTCCCATGGGGGACTCCCTGGGCAGTTTTCGACTTCTAAAAGCAAGTCATACCCGTC
Allele C4    GCCTTCCCATGGGGGACTCCCTGGGCAGTTTTCGACTTCTAAAAGCAAGTCATACCCGTC
Allele S1    GCCTTCCCATGGGGGACTCCCTGGGCAGTTTTCGACTTCTAAAAGCAAGTCATACCCGTC
                                         7
                   310       320       330       340       350       360
NvNFkB_C67   TGTCCAGGTAATTCTATTGTTTACCTCGTGAACAACTACCAAGGACCATGTAGAATAGTT
NvNFkB_S67   TGTCCAGGTAATTCTATTGTTTACCTGGTGAACAACTACCAAGGACCATGTAGAATAGTT
               V  Q [....intronic......] V  N  N  Y  Q  G  P  C  R  I  V
Allele C1    TGTCCAGGTAATTCTATTGTTTACCTC
Allele C2    TGTCCAGGTAATTCTATTGTTTACCTG
Allele C3    TGTCCAGGTAATTCTATTGTTTACCTG
Allele C4    TGTCCAGGTAATTCTATTGTTTACCTC
Allele S1    TGTCCAGGTAATTCTATTGTTTACCTG
                   370       380       390       400       410       420
NvNFkB_C67   GTAACCTTGGTTACAAAAGATGAACCTTATATGCTACATGCACATAGTTTAACTGGGAAG
NvNFkB_S67   GTAACCTTGGTCACAAAAGATGAACCTTATATGCTACATGCACATAGTTTAACTGGGAAG
              V  T  L  V  T  K  D  E  P  Y  M  L  H  A  H  S  L  T  G  K
                   430       440       450       460       470       480
NvNFkB_C67   AATGCTAATGAAGAAGGAGTAGTCACCGTGCAAGTGGGGCCGGATCAACACATGACAGCG
NvNFkB_S67   AATGCTAATGAAGAAGGAGTAGTCACCGTACAAGTGGGGCCGGATCAACACATGACAGCG
              N  A  N  E  E  G  V  V  T  V  Q  V  G  P  D  Q  H  M  T  A
                   490       500       510       520       530       540
NvNFkB_C67   AGCTTCCCAAACTTGGGAATCCAGCATGTCACTAAAAAGAATGTTGTTAAAGTACTAATG
```

```
NvNFkB_S67  AGCTTCCCAAACTTGGGAATCCAGCATGTCACTAAAAAGAATGTTGTTAAAGTACTAATG
             S  F  P  N  L  G  I  Q  H  V  T  K  K  N  V  V  K  V  L  M
                     550       560       570       580       590       600
NvNFkB_C67  GATAGATTTATAAAGTGGCAAACTCTGCAAAATGCAACTTTCGCCAAATTGTCAGAAGGT
NvNFkB_S67  GATAGATTTATAAAGTGGCAAACTCTGCAAAATGCAACTTTCGCCAAATTGTCAGAAGGT
             D  R  F  I  K  W  Q  T  L  Q  N  A  T  F  A  K  L  S  E  G
                     610       620       630       640       650       660
NvNFkB_C67  ATCAAGGATGGTGTAGACCTATCACTCTTTGGTGTCAATACAGCCATAAACTCAAACAAG
NvNFkB_S67  ATCAAGGATGGTGTAGACCTATCACTCTTTGGTGTCAATACAGCCATAAACTCAAACAAG
             I  K  D  G  V  D  L  S  L  F  G  V  N  T  A  I  N  S  N  K
                     670       680       690       700       710       720
NvNFkB_C67  CTTGGGTTTGACAAAAGTGTGGCGCTCTCTGTCGCAAACGAAGAAGTTGCCAAAAGTAGA
NvNFkB_S67  CTTGGGTTTGACAAAAATGTGGCGCTCTCTGTCGCAAACCAAGAAGCTGCCAAAAGTAGA
             L  G  F  D  K S/N V  A  L  S  V  A  N E/Q E V/A A  K  S  R
                     730       740       750       760       770       780
NvNFkB_C67  GAGTACGCTGAGCAACAGGCCGCTGCCATGGATCTAAGTGCTGTGAGACTCTGCTTCCAG
NvNFkB_S67  GAGTACGCTAAGCAACAGGCCGCTGCCATGGATCTAAGTGCTGTGAGACTCTGCTTCCAG
             E  Y  A E/K Q  Q  A  A  A  M  D  L  S  A  V  R  L  C  F  Q
                     790       800       810       820       830       840
NvNFkB_C67  GCATACCTACCAGACCAAGACGGCAACTTTACCAGACCTCTGAAGCCAGTGTACTCAGAT
NvNFkB_S67  GCATACCTACCAGACCAAGATGGCAACTTTACCAGACCTCTGAAGCCAGTGTACTCAGAT
             A  Y  L  P  D  Q  D  G  N  F  T  R  P  L  K  P  V  Y  S  D
                     850       860       870       880       890       900
NvNFkB_C67  GCTGTCTTGGACTCAAAAGCACCATCAGCATCTCAATTGAAAATCTGTCGCATGGACAAG
NvNFkB_S67  GCTGTCTTGGACTCAAAAGAACCATCAGCATCTCAATTGAAAATCTGTCGCATGGACAAG
             A  V  L  D  S  K A/E P  S  A  S  Q  L  K  I  C  R  M  D  K
                     910       920       930       940       950       960
NvNFkB_C67  AACTCAGGATGTGTAACTGGTGGTGATGAAATATACCTGCTTTGTGACAAAGTTCAGAAA
NvNFkB_S67  AACTCGGGATGTGTAACTGGTGGTGATGAAATATACCTGCTTTGTGACAAAGTTCAGAAG
             N  S  G  C  V  T  G  G  D  E  I  Y  L  L  C  D  K  V  Q  K
                     970       980       990      1000      1010      1020
NvNFkB_C67  GATGACATTGAGATACACTTTTATGAAATGGATGATATCACTGGCAAGTACACATGGGAA
NvNFkB_S67  GATGACATTGAGATACACTTTTATGAAATGGATGATATCACTGGCAAGTACACATGGGAA
             D  D  I  E  I  H  F  Y  E  M  D  D  I  T  G  K  Y  T  W  E
                    1030      1040      1050      1060      1070      1080
NvNFkB_C67  GATTTAGGGAAGTTCTCTCCATGCGATGTGCACCGACAGTTTGCCATAGTGTTCAAAACT
NvNFkb_S67  GATTTAGGGAAGTTCTCTCCATGCGATGTGCACCGACAGTTTGCCATAGTGTTCAAAACT
             D  L  G  K  F  S  P  C  D  V  H  R  Q  F  A  I  V  F  K  T
                    1090      1100      1110      1120      1130      1140
NvNFkB_C67  CCGCCTTATTGGAACATTGCTATCGAGAGACCAGCAAATGTCCTTGTAGAGCTTCGACGT
NvNFkB_S67  CCGCCTTATTGGAACATTGCTATCGAGAGACCAGCAAATGTCCTTGTAGAGCTTCGACGT
             P  P  Y  W  N  I  A  I  E  R  P  A  N  V  L  V  E  L  R  R
                    1150      1160      1170      1180      1190      1200
NvNFkB_C67  AAGAAGAATGGTGGGGAAACAAGTGAACCTGTGCAATTCACTTACCAGCCACAACTTTTT
NvNFkB_S67  AAGAAGAATGGTGGGGAAACAAGTGAACCTGTGCAATTCACTTACCAGCCACAACTTTTT
             K  K  N  G  G  E  T  S  E  P  V  Q  F  T  Y  Q  P  Q  L  F
                    1210      1220      1230      1240      1250      1260
NvNFkB_C67  GACAAGGAGGCGATTGGTGCCAAACGCCGCAAGACAGTGCCACACTTTACCGACTTTCTT
NvNKkB_S67  GACAAGGAGGCGATTGGTGCCAAACGCCGCAAGACAGTGCCACACTTTACCGAATTTCTT
             D  K  E  A  I  G  A  K  R  R  K  T  V  P  H  F  T D/E F  L
                    1270      1280      1290      1300      1310      1320
NvNFkB_C67  TCCGGCGGCTCATCTGGAGCTACAGGTGGAGGGGGAAGTTCAGTGTCCGGATTCAACTTC
NvNFkB_S67  TCCGGCGGCTCATCTGGAGCTACAGGTGGAGGGGGAAGTTCAGTGTCCGGATTCAACTTC
             S  G  G  S  S  G  A  T  G  G  G  G  S  S  V  S  G  F  N  F
                    1330      1340      1350      1360      1370
NvNFkB_C67  CCAGCTGATTTTCTTCAGCAGGGGGTGTTCTCCACCCAAAACCCTTCAAACATGTGA
NvNFkB_S67  TCAGCTGATTTTCTTCAGCAGGGGGTGTTCCTCACCCAAAACCCTTCAAACATGTGA
            P/S A  D  F  L  Q  Q  G  V  F S/L T  Q  N  P  S  N  M  *
```
