# Supplementary material for: Two Alleles of NF-κB in the Sea Anemone Nematostella vectensis Are Widely Dispersed in Nature and Encode Proteins with Distinct Activities
Source: PLoS One. 2009 Oct 6;4(10):e7311. doi: 10.1371/journal.pone.0007311 (PMC2751831; doi:10.1371/journal.pone.0007311)
Supplement: Figure S3 — Alignment of Nematostella proteins against partial coral NF-κB protein. A partial coding sequence for the NF-κB gene of Acropora millepora was recently recovered in a transcriptome sequencing project [7]. The predicted coral protein (AmNFκB) was aligned against the Nematostella proteins using a web implementation of ClustalW2 [39]. The positions that are polymorphic in Nematostella are indicated by bold red type. Invariant positions are indicated by asterisks. (0.06 MB PDF) [file pone.0007311.s003.pdf]

Supplemental Figure 3 Alignment of *Nematostella* and *Acropora* NF-κB proteins

```
NvNFkB_C  MAQ-SEQQVGSALTESMLNEIIQPGYLPDISALHVPLGTNAEEPSYTEPYLEILEQPKPR 59
NvNFkB_S  MAQ-SEQQVGSALTESMLNEIIRPGYLPDISALHVPLGTNAEEPSYTEPYLEILEQPKPR 59
AmNFkB    MATNSEQQVNATLTDSLLMDVLNPGYLPDISALQVPTG------SYQGPYLEILEQPKQR 54
          **  *****   ** * *     ********** ** *      **  ********** *

NvNFkB_C  GFRFRYPCEGPSHGGLPGQFSTSKSKSYPSVQVNNYQGPCRIVVTLVTKDEPYMLHAHSL 119
NvNFkB_S  GFRFRYPSEGPSHGGLPGQFSTSKSKSYPSVQVNNYQGPCRIVVTLVTKDEPYMLHAHSL 119
AmNFkB    GFRFRYPCEGPSHGGLPGEYSE-KGKSYPSVQLCNYKGPARIVVSLVTSDENPMPHAHSL 113
          ******* **********  *  * *******  ** ** **** *** **  * *****

NvNFkB_C TGKNANEEGVVTVQVGPDQHMTASFPNLGIQHVTKKNVVKVLMDRFIKWQTLQNATFAKL 179
NvNFkB_S TGKNANEEGVVTVQVGPDQHMTASFPNLGIQHVTKKNVVKVLMDRFIKWQTLQNATFAKL 179
AmNFkB   IGKNASN-GVVTVQIGPEQGMTASFPNLGIQHVTKKNVGLVLMDRYLKMQMLHTATLNAL 172
          ****   ****** ** * ******************  *****  * * *  **   *

NvNFkB_C SEGIKDGVDLSLFGVNTAINSNKLGFDKSVALSVANEEVAKSREYAEQQAAAMDLSAVRL 239
NvNFkB_S SEGIKDGVDLSLFGVNTAINSNKLGFDKNVALSVANQEAAKSREYAKQQAAAMDLSAVRL 239
AmNFkB   TIDPR-VFDIGAMVDEATADGDRGEFDKQIADTIAEEEASKVRSLVEKQKNSMNLSVVRL 231
                 *                ***  *   *  *  * *     *   * ** ***

NvNFkB_C CFQAYLPDQDGNFTRPLKPVYSDAVLDSKAPSASQLKICRMDKNSGCVTGGDEIYLLCDK 299
NvNFkB_S CFQAYLPDQDGNFTRPLKPVYSDAVLDSKEPSASQLKICRMDKNSGCVTGGDEIYLLCDK 299
AmNFkB   CFQAYLPDDNGCFTKALPPCFSRSVYDSKAPSAANLKICRMDRNSGCVTGNDEVYLLCDK 291
         ********  * **  * *  *  * *** ***  ******* ******* ** ******

NvNFkB_C VQKDDIEIHFYEMDDITGKYTWEDLGKFSPCDVHRQFAIVFKTPPYWNIAIERPANVLVE 359
NvNFkB_S VQKDDIEIHFYEMDDITGKYTWEDLGKFSPCDVHRQFAIVFKTPPYWNIAIERPANVLVE 359
AmNFkB   VQKDDIAVVFYEIDEN-GKRSWEGQGLLRP?????????????????????????????? 320
         ******   *** *   **  **  *   *

NvNFkB_C LRRKKNGGETSEPVQFTYQPQLFDKEAIGAKRRKTVPHFTDFLSGGSSGATGGGGSSVSG 419
NvNFkB_S LRRKKNGGETSEPVQFTYQPQLFDKEAIGAKRRKTVPHFTEFLSGGSSGATGGGGSSVSG 419
AmNFkB   ????????????????????????????????????????????????????????????

NvNFkB_C FNFPADFLQQGVFSTQNPSNM 440
NvNFkB_S FNFSADFLQQGVFLTQNPSNM 440
AmNFkB   ?????????????????????
```
