# Supplementary material for: Two Alleles of NF-κB in the Sea Anemone Nematostella vectensis Are Widely Dispersed in Nature and Encode Proteins with Distinct Activities
Source: PLoS One. 2009 Oct 6;4(10):e7311. doi: 10.1371/journal.pone.0007311 (PMC2751831; doi:10.1371/journal.pone.0007311)
Supplement: Figure S4 — Alignment of 159-nt region of Nv-NF-κB from 403 individual animals. Where a position in the sequence chromatogram harbored peaks for two different nucleotides, the appropriate IUPAC code is used (K = G or T; R = A or G; S = C or G; W = A or T). (0.81 MB PDF) [file pone.0007311.s004.pdf]

Sample	1 – 80
NS D88 02 CS	AAAAAGCATGGAAAKAATGATTAATTGAATTAWTTKTAGCGTGGTTTCCGRTTCCGATACCCAWGTGAAGGGCCTTCCCA
NS D88 03 CC	AAAAAGCATGGAAAGAATGATTAATTGAATTAATTGTAGCGTGGTTTCCGGTTCCGATACCCATGTGAAGGGCCTTCCCA
NS D88 04 CS	AAAAAGCATGGAAAKAATGATTAATTGAATTAWTTKTAGCGTGGTTTCCGRTTCCGATACCCAWGTGAAGGGCCTTCCCA
NS D296 03 SS	AAAAAGCATGGAAATAATGATTAATTGAATTATTTTTAGCGTGGTTTCCGATTCCGATACCCAAGTGAAGGGCCTTCCCA
NS D296 07 CS	AAAAAGCATGGAAAKAATGATTAATTGAATTAWTTKTAGCGTGGTTTCCGRTTCCGATACCCANGTGAAGGGCCTTCCCA
NS D296 08 CS	AAAAAGCATGGAAAKAATGATTAATTGAATTAWTTKTAGCGTGGTTTCCGRTTCCGATACCCAWGTGAAGGGCCTTCCCA
NS D296 09 CS	AAAAAGCATGGAAAKAATGATTAATTGAATTAWTTKTAGCGTGGTTTCCGRTTCCGATACCCAWGTGAAGGGCCTTCCCA
NS D296 11 CS	AAAAAGCATGGAAAKAATGATTAATTGAATTAWTTKTAGCGTGGTTTCCGRTTCCGATACCCAWGTGAAGGGCCTTCCCA
NS D296 12 CS	AAAAAGCATGGAAAKAATGATTAATTGAATTAWTTKTAGCGTGGTTTCCGRTTCCGATACCCAWGTGAAGGGCCTTCCCA
Hal1 4 CS	AAAAAGCATGGAAAKAATGATTAATTGAATTAWTTKTAGCGTGGTTTCCGRTTCCGATACCCAWGTGAAGGGCCTTCCCA
Hal1 5 CS	AAAAAGCATGGAAAKAATGATTAATTGAATTAWTTKTAGCGTGGTTTCCGRTTCCGATACCCAWGTGAAGGGCCTTCCCA
Hal1 6 CS	AAAAAGCATGGAAAKAATGATTAATTGAATTAWTTKTAGCGTGGTTTCCGRTTCCGATACCCAWGTGAAGGGCCTTCCCA
Hal1 7 CS	AAAAAGCATGGAAAKAATGATTAATTGAATTAWTTKTAGCGTGGTTTCCGRTTCCGATACCCAWGTGAAGGGCCTTCCCA
Hal1 8 CS	AAAAAGCATGGAAAKAATGATTAATTGAATTAWTTKTAGCGTGGTTTCCGRTTCCGATACCCAWGTGAAGGGCCTTCCCA
Hal1 9 SS	AAAAAGCATGGAAATAATGATTAATTGAATTATTTTTAGCGTGGTTTCCGATTCCGATACCCAAGTGAAGGGCCTTCCCA
Hal1 10 CS	AAAAAGCATGGAAAKAATGATTAATTGAATTAWTTKTAGCGTGGTTTCCGRTTCCGATACCCAWGTGAAGGGCCTTCCCA
Hal1 11 CS	AAAAAGCATGGAAAKAATGATTAATTGAATTAWTTKTAGCGTGGTTTCCGRTTCCGATACCCAWGTGAAGGGCCTTCCCA
Hal1 12 CS	AAAAAGCATGGAAAKAATGATTAATTGAATTAWTTKTAGCGTGGTTTCCGRTTCCGATACCCAWGTGAAGGGCCTTCCCA
Hal1 14 CS	AAAAAGCATGGAAAKAATGATTAATTGAATTAWTTKTAGCGTGGTTTCCGRTTCCGATACCCAWGTGAAGGGCCTTCCCA
Hal1 15 CS	AAAAAGCATGGAAAKAATGATTAATTGAATTAWTTKTAGCGTGGTTTCCGRTTCCGATACCCAWGTGAAGGGCCTTCCCA
Hal1 16 CS	AAAAAGCATGGAAAKAATGATTAATTGAATTAWTTKTAGCGTGGTTTCCGRTTCCGATACCCAWGTGAAGGGCCTTCCCA
Hal1 17 CS	AAAAAGCATGGAAAKAATGATTAATTGAATTAWTTKTAGCGTGGTTTCCGRTTCCGATACCCAWGTGAAGGGCCTTCCCA
Hal1 19 CS	AAAAAGCATGGAAAKAATGATTAATTGAATTAWTTKTAGCGTGGTTTCCGRTTCCGATACCCAWGTGAAGGGCCTTCCCA
Hal1 20 CS	AAAAAGCATGGAAAKAATGATTAATTGAATTAWTTKTAGCGTGGTTTCCGRTTCCGATACCCAWGTGAAGGGCCTTCCCA
Hal1 21 SS	AAAAAGCATGGAAATAATGATTAATTGAATTATTTTTAGCGTGGTTTCCGATTCCGATACCCAAGTGAAGGGCCTTCCCA
Hal1 23 CS	AAAAAGCATGGAAAKAATGATTAATTGAATTAWTTKTAGCGTGGTTTCCGRTTCCGATACCCAWGTGAAGGGCCTTCCCA
NS F299 1 SS	AAAAAGCATGGAAATAATGATTAATTGAATTATTTTTAGCGTGGTTTCCGATTCCGATACCCAAGTGAAGGGCCTTCCCA
NS F299 3 SS	AAAAAGCATGGAAATAATGATTAATTGAATTATTTTTAGCGTGGTTTCCGATTCCGATACCCAAGTGAAGGGCCTTCCCA
NS F299 4 CC	AAAAAGCATGGAAAGAATGATTAATTGAATTAATTGTAGCGTGGTTTCCGGTTCCGATACCCATGTGAAGGGCCTTCCCA
NS F299 5 SS	AAAAAGCATGGAAATAATGATTAATTGAATTATTTTTAGCGTGGTTTCCGATTCCGATACCCAAGTGAAGGGCCTTCCCA
NS F299 6 SS	AAAAAGCATGGAAATAATGATTAATTGAATTATTTTTAGCGTGGTTTCCGATTCCGATACCCAAGTGAAGGGCCTTCCCA
NS F 299 8 SS	AAAAAGCATGGAAATAATGATTAATTGAATTATTTTTAGCGTGGTTTCCGATTCCGATACCCAAGTGAAGGGCCTTCCCA
NS F299 9 SS	AAAAAGCATGGAAATAATGATTAATTGAATTATTTTTAGCGTGGTTTCCGATTCCGATACCCAAGTGAAGGGCCTTCCCA
NS F299 10 SS	AAAAAGCATGGAAATAATGATTAATTGAATTATTTTTAGCGTGGTTTCCGATTCCGATACCCAAGTGAAGGGCCTTCCCA
NS F299 11 SS	AAAAAGCATGGAAATAATGATTAATTGAATTATTTTTAGCGTGGTTTCCGATTCCGATACCCAAGTGAAGGGCCTTCCCA
NS B293 1 CC	AAAAAGCATGGAAAGAATGATTAATTGAATTAATTGTAGCGTGGTTTCCGGTTCCGATACCCATGTGAAGGGCCTTCCCA
NS B293 2 CC	AAAAAGCATGGAAAGAATGATTAATTGAATTAATTGTAGCGTGGTTTCCGGTTCCGATACCCATGTGAAGGGCCTTCCCA
NS B293 3 CC	AAAAAGCATGGAAAGAATGATTAATTGAATTAATTGTAGCGTGGTTTCCGGTTCCGATACCCATGTGAAGGGCCTTCCCA
NS B293 4 CC	AAAAAGCATGGAAAGAATGATTAATTGAATTAATTGTAGCGTGGTTTCCGGTTCCGATACCCATGTGAAGGGCCTTCCCA
NS B293 5 CC	AAAAAGCATGGAAAGAATGATTAATTGAATTAATTGTAGCGTGGTTTCCGGTTCCGATACCCATGTGAAGGGCCTTCCCA
NS B293 6 Cy	AAAAAGCATGGAAAGAATGATTAATTGAATTAATTGTAGCGTGGTTTCCGGTTCCGATACCCATGTGAAGGGCCTTCCCA
NS B293 7 CC	AAAAAGCATGGAAAGAATGATTAATTGAATTAATTGTAGCGTGGTTTCCGGTTCCGATACCCATGTGAAGGGCCTTCCCA
NS B293 8 CC	AAAAAGCATGGAAAGAATGATTAATTGAATTAATTGTAGCGTGGTTTCCGGTTCCGATACCCATGTGAAGGGCCTTCCCA
NS B293 9 CC	AAAAAGCATGGAAAGAATGATTAATTGAATTAATTGTAGCGTGGTTTCCGGTTCCGATACCCATGTGAAGGGCCTTCCCA
NS B293 10 CC	AAAAAGCATGGAAAGAATGATTAATTGAATTAATTGTAGCGTGGTTTCCGGTTCCGATACCCATGTGAAGGGCCTTCCCA
NS B293 11 CC	AAAAAGCATGGAAAGAATGATTAATTGAATTAATTGTAGCGTGGTTTCCGGTTCCGATACCCATGTGAAGGGCCTTCCCA
NS B294 1 CC	AAAAAGCATGGAAAGAATGATTAATTGAATTAATTGTAGCGTGGTTTCCGGTTCCGATACCCATGTGAAGGGCCTTCCCA
NS B294 2 CC	AAAAAGCATGGAAAGAATGATTAATTGAATTAATTGTAGCGTGGTTTCCGGTTCCGATACCCATGTGAAGGGCCTTCCCA
NS B294 4 CC	AAAAAGCATGGAAAGAATGATTAATTGAATTAATTGTAGCGTGGTTTCCGGTTCCGATACCCATGTGAAGGGCCTTCCCA
NS B294 5 CC	AAAAAGCATGGAAAGAATGATTAATTGAATTAATTGTAGCGTGGTTTCCGGTTCCGATACCCATGTGAAGGGCCTTCCCA
NS B294 6 CC	AAAAAGCATGGAAAGAATGATTAATTGAATTAATTGTAGCGTGGTTTCCGGTTCCGATACCCATGTGAAGGGCCTTCCCA

```
             1        10        20        30        40        50        60        70       80
NS B294 7 CC  AAAAAGCATGGAAAGAATGATTAATTGAATTAATTGTAGCGTGGTTTCCGGTTCCGATACCCATGTGAAGGGCCTTCCCA
NS B294 9 CC  AAAAAGCATGGAAAGAATGATTAATTGAATTAATTGTAGCGTGGTTTCCGGTTCCGATACCCATGTGAAGGGCCTTCCCA
NS B294 10 CC AAAAAGCATGGAAAGAATGATTAATTGAATTAATTGTAGCGTGGTTTCCGGTTCCGATACCCATGTGAAGGGCCTTCCCA
NS A291 3 CS  AAAAAGCATGGAAAKAATGATTAATTGAATTAWTTKTAGCGTGGTTTCCGRTTCCGATACCCAWGTGAAGGGCCTTCCCA
NS A291 4 CC  AAAAAGCATGGAAAGAATGATTAATTGAATTAATTGTAGCGTGGTTTCCGGTTCCGATACCCATGTGAAGGGCCTTCCCA
NS A291 5 CS  AAAAAGCATGGAAAKAATGATTAATTGAATTAWTTKTAGCGTGGTTTCCGRTTCCGATACCCAWGTGAAGGGCCTTCCCA
NS A291 6 CS  AAAAAGCATGGAAAKAATGATTAATTGAATTAWTTKTAGCGTGGTTTCCGRTTCCGATACCCAWGTGAAGGGCCTTCCCA
NS A291 7 CS  AAAAAGCATGGAAAKAATGATTAATTGAATTAWTTKTAGCGTGGTTTCCGRTTCCGATACCCAWGTGAAGGGCCTTCCCA
NS A291 8 CS  AAAAAGCATGGAAAKAATGATTAATTGAATTAWTTKTAGCGTGGTTTCCGRTTCCGATACCCAWGTGAAGGGCCTTCCCA
NS A291 10 CS AAAAAGCATGGAAAKAATGATTAATTGAATTAWTTKTAGCGTGGTTTCCGRTTCCGATACCCAWGTGAAGGGCCTTCCCA
NS A291 11 CS AAAAAGCATGGAAAKAATGATTAATTGAATTAWTTKTAGCGTGGTTTCCGRTTCCGATACCCAWGTGAAGGGCCTTCCCA
NS A291 12 CS AAAAAGCATGGAAAKAATGATTAATTGAATTAWTTKTAGCGTGGTTTCCGRTTCCGATACCCAWGTGAAGGGCCTTCCCA
NS A291 13 CS AAAAAGCATGGAAAKAATGATTAATTGAATTAWTTKTAGCGTGGTTTCCGRTTCCGATACCCAWGTGAAGGGCCTTCCCA
NS A291 15 CS AAAAAGCATGGAAAKAATGATTAATTGAATTAWTTKTAGCGTGGTTTCCGRTTCCGATACCCAWGTGAAGGGCCTTCCCA
NS A291 17 CS AAAAAGCATGGAAAKAATGATTAATTGAATTAWTTKTAGCGTGGTTTCCGRTTCCGATACCCAWGTGAAGGGCCTTCCCA
NS A291 18 CS AAAAAGCATGGAAAKAATGATTAATTGAATTAWTTKTAGCGTGGTTTCCGRTTCCGATACCCAWGTGAAGGGCCTTCCCA
NS A291 19 CC AAAAAGCATGGAAAGAATGATTAATTGAATTAATTGTAGCGTGGTTTCCGGTTCCGATACCCATGTGAAGGGCCTTCCCA
NS A291 20 CS AAAAAGCATGGAAAKAATGATTAATTGAATTAWTTKTAGCGTGGTTTCCGRTTCCGATACCCAWGTGAAGGGCCTTCCCA
NS A291 21 CS AAAAAGCATGGAAAKAATGATTAATTGAATTAWTTKTAGCGTGGTTTCCGRTTCCGATACCCAWGTGAAGGGCCTTCCCA
NS E298 1 CC  AAAAAGCATGGAAAGAATGATTAATTGAATTAATTGTAGCGTGGTTTCCGGTTCCGATACCCATGTGAAGGGCCTTCCCA
NS E298 2 CC  AAAAAGCATGGAAAGAATGATTAATTGAATTAATTGTAGCGTGGTTTCCGGTTCCGATACCCATGTGAAGGGCCTTCCCA
NS E298 3 CC  AAAAAGCATGGAAAGAATGATTAATTGAATTAATTGTAGCGTGGTTTCCGGTTCCGATACCCATGTGAAGGGCCTTCCCA
NS E298 4 SS  AAAAAGCATGGAAATAATGATTAATTGAATTATTTTTAGCGTGGTTTCCGATTCCGATACCCAAGTGAAGGGCCTTCCCA
NS E298 5 CC  AAAAAGCATGGAAAGAATGATTAATTGAATTAATTGTAGCGTGGTTTCCGGTTCCGATACCCATGTGAAGGGCCTTCCCA
NS E298 6 CC  AAAAAGCATGGAAAGAATGATTAATTGAATTAATTGTAGCGTGGTTTCCGGTTCCGATACCCATGTGAAGGGCCTTCCCA
NS E298 7 CC  AAAAAGCATGGAAAGAATGATTAATTGAATTAATTGTAGCGTGGTTTCCGGTTCCGATACCCATGTGAAGGGCCTTCCCA
NS E298 8 CC  AAAAAGCATGGAAAGAATGATTAATTGAATTAATTGTAGCGTGGTTTCCGGTTCCGATACCCATGTGAAGGGCCTTCCCA
NS E298 9 CC  AAAAAGCATGGAAAGAATGATTAATTGAATTAATTGTAGCGTGGTTTCCGGTTCCGATACCCATGTGAAGGGCCTTCCCA
NS E298 10 CC AAAAAGCATGGAAAGAATGATTAATTGAATTAATTGTAGCGTGGTTTCCGGTTCCGATACCCATGTGAAGGGCCTTCCCA
KNG 1 1 CC     AAAAAGCATGGAAAGAATGATTAATTGAATTAATTGTAGCGTGGTTTCCGGTTCCGATACCCATGTGAAGGGCCTTCCCA
KNG 1 2 CC     AAAAARCATGGAAAGAATGATTAATTGAATTAATTGTAGCGTGGTTTCCGGTTCCGATACCCATGTGAAGGGCCTTCCCA
KNG 1 3 CC     AAAAARCATGGAAAGAATGATTAATTGAATTAATTGTAGCGTGGTTTCCGGTTCCGATACCCATGTGAAGGGCCTTCCCA
KNG 1 4 CC     AAAAARCATGGAAAGAATGATTAATTGAATTAATTGTAGCGTGGTTTCCGGTTCCGATACCCATGTGAAGGGCCTTCCCA
KNG 1 5 CC     AAAAAGCATGGAAAGAATGATTAATTGAATTAATTGTAGCGTGGTTTCCGGTTCCGATACCCATGTGAAGGGCCTTCCCA
KNG 1 6 CC     AAAAARCATGGAAAGAATGATTAATTGAATTAATTGTAGCGTGGTTTCCGGTTCCGATACCCATGTGAAGGGCCTTCCCA
KNG 1 7 CC     AAAAARCATGGAAAGAATGATTAATTGAATTAATTGTAGCGTGGTTTCCGGTTCCGATACCCATGTGAAGGGCCTTCCCA
KNG 1 8 CC     AAAAARCATGGAAAGAATGATTAATTGAATTAATTGTAGCGTGGTTTCCGGTTCCGATACCCATGTGAAGGGCCTTCCCA
KNG 1 9 CC     AAAAARCATGGAAAGAATGATTAATTGAATTAATTGTAGCGTGGTTTCCGGTTCCGATACCCATGTGAAGGGCCTTCCCA
KNG 1 11 CC    AAAAARCATGGAAAGAATGATTAATTGAATTAATTGTAGCGTGGTTTCCGGTTCCGATACCCATGTGAAGGGCCTTCCCA
KNG 1 12 CC    AAAAARCATGGAAAGAATGATTAATTGAATTAATTGTAGCGTGGTTTCCGGTTCCGATACCCATGTGAAGGGCCTTCCCA
KNG 1 13 CC    AAAAARCATGGAAAGAATGATTAATTGAATTAATTGTAGCGTGGTTTCCGGTTCCGATACCCATGTGAAGGGCCTTCCCA
KNG 2 1 CC     AAAAARCATGGAAAGAATGATTAATTGAATTAATTGTAGCGTGGTTTCCGGTTCCGATACCCATGTGAAGGGCCTTCCCA
KNG 2 2 CC     AAAAAGCATGGAAAGAATGATTAATTGAATTAATTGTAGCGTGGTTTCCGGTTCCGATACCCATGTGAAGGGCCTTCCCA
KNG 2 5 CC     AAAAARCATGGAAAGAATGATTAATTGAATTAATTGTAGCGTGGTTTCCGGTTCCGATACCCATGTGAAGGGCCTTCCCA
KNG 2 6 CC     AAAAAGCATGGAAAGAATGATTAATTGAATTAATTGTAGCGTGGTTTCCGGTTCCGATACCCATGTGAAGGGCCTTCCCA
KNG 2 7 CC     AAAAAGCATGGAAAGAATGATTAATTGAATTAATTGTAGCGTGGTTTCCGGTTCCGATACCCATGTGAAGGGCCTTCCCA
KNG 2 8 CC     AAAAAGCATGGAAAGAATGATTAATTGAATTAATTGTAGCGTGGTTTCCGGTTCCGATACCCATGTGAAGGGCCTTCCCA
KNG 2 9 CC     AAAAAGCATGGAAAGAATGATTAATTGAATTAATTGTAGCGTGGTTTCCGGTTCCGATACCCATGTGAAGGGCCTTCCCA
KNG 3 2 CC     AAAAAGCATGGAAAGAATGATTAATTGAATTAATTGTAGCGTGGTTTCCGGTTCCGATACCCATGTGAAGGGCCTTCCCA
KNG 3 3 CC     AAAAARCATGGAAAGAATGATTAATTGAATTAATTGTAGCGTGGTTTCCGGTTCCGATACCCATGTGAAGGGCCTTCCCA
KNG 3 4 CC     NNAAARCATGGAAAGAATGATTAATTGAATTAATTGTAGCGTGGTTTCCGGTTCCGATACCCATGTGAAGGGCCTTCCCA
```

```
             1        10        20        30        40        50        60        70        80
KNG 3 5 CC   AAAAAGCATGGAAAGAATGATTAATTGAATTAATTGTAGCGTGGTTTCCGGTTCCGATACCCATGTGAAGGGCCTTCCCA
KNG 3 6 CC   AAAAAGCATGGAAAGAATGATTAATTGAATTAATTGTAGCGTGGTTTCCGGTTCCGATACCCATGTGAAGGGCCTTCCCA
KNG 3 7 CC   AAAAAGCATGGAAAGAATGATTAATTGAATTAATTGTAGCGTGGTTTCCGGTTCCGATACCCATGTGAAGGGCCTTCCCA
KNG 3 8 CC   AAAAAGCATGGAAAGAATGATTAATTGAATTAATTGTAGCGTGGTTTCCGGTTCCGATACCCATGTGAAGGGCCTTCCCA
KNG 3 9 CC   AAAAAGCATGGAAAGAATGATTAATTGAATTAATTGTAGCGTGGTTTCCGGTTCCGATACCCATGTGAAGGGCCTTCCCA
KNG 3 10 CC  AAAAAGCATGGAAAGAATGATTAATTGAATTAATTGTAGCGTGGTTTCCGGTTCCGATACCCATGTGAAGGGCCTTCCCA
KNG 3 14-CC  AAAAAGCATGGAAAGAATGATTAATTGAATTAATTGTAGCGTGGTTTCCGGTTCCGATACCCATGTGAAGGGCCTTCCCA
NS C1 CC     AAAAAGCATGGAAAGAATGATTAATTGAATTAATTGTAGCGTGGTTTCCGGTTCCGATACCCATGTGAAGGGCCTTCCCA
NS C2 CC     AAAAAGCATGGAAAGAATGATTAATTGAATTAATTGTAGCGTGGTTTCCGGTTCCGATACCCATGTGAAGGGCCTTCCCA
NS C3 CC     AAAAAGCATGGAAAGAATGATTAATTGAATTAATTGTAGCGTGGTTTCCGGTTCCGATACCCATGTGAAGGGCCTTCCCA
NS C4 CC     AAAAAGCATGGAAAGAATGATTAATTGAATTAATTGTAGCGTGGTTTCCGGTTCCGATACCCATGTGAAGGGCCTTCCCA
NS C6 CC     AAAAAGCATGGAAAGAATGATTAATTGAATTAATTGTAGCGTGGTTTCCGGTTCCGATACCCATGTGAAGGGCCTTCCCA
NS C9 CC     AAAAAGCATGGAAAGAATGATTAATTGAATTAATTGTAGCGTGGTTTCCGGTTCCGATACCCATGTGAAGGGCCTTCCCA
OD 1 CC      AAAAAGCATGGAAAGAATGATTAATTGAATTAATTGTAGCGTGGTTTCCGGTTCCGATACCCATGTGAAGGGCCTTCCCA
OD 2 CC      AAAAAGCATGGAAAGAATGATTAATTGAATTAATTGTAGCGTGGTTTCCGGTTCCGATACCCATGTGAAGGGCCTTCCCA
OD 3 CC      AAAAAGCATGGAAAGAATGATTAATTGAATTAATTGTAGCGTGGTTTCCGGTTCCGATACCCATGTGAAGGGCCTTCCCA
OD 4 CC      AAAAAGCATGGAAAGAATGATTAATTGAATTAATTGTAGCGTGGTTTCCGGTTCCGATACCCATGTGAAGGGCCTTCCCA
OD 5 CC      AAAAAGCATGGAAAGAATGATTAATTGAATTAATTGTAGCGTGGTTTCCGGTTCCGATACCCATGTGAAGGGCCTTCCCA
OD 9 CC      AAAAAGCATGGAAAGAATGATTAATTGAATTAATTGTAGCGTGGTTTCCGGTTCCGATACCCATGTGAAGGGCCTTCCCA
OD2004 3 CC  AAAAAGCATGGAAAGAATGATTAATTGAATTAATTGTAGCGTGGTTTCCGGTTCCGATACCCATGTGAAGGGCCTTCCCA
OD2004 4 CC  AAAAAGCATGGAAAGAATGATTAATTGAATTAATTGTAGCGTGGTTTCCGGTTCCGATACCCATGTGAAGGGCCTTCCCA
OD2004 6 CC  AAAAAGCATGGAAAGAATGATTAATTGAATTAATTGTAGCGTGGTTTCCGGTTCCGATACCCATGTGAAGGGCCTTCCCA
OD2004 7 CC  AAAAAGCATGGAAAGAATGATTAATTGAATTAATTGTAGCGTGGTTTCCGGTTCCGATACCCATGTGAAGGGCCTTCCCA
OD2004 10 CC AAAAAGCATGGAAAGAATGATTAATTGAATTAATTGTAGCGTGGTTTCCGGTTCCGATACCCATGTGAAGGGCCTTCCCA
OD2004 14 CC AAAAAGCATGGAAAGAATGATTAATTGAATTAATTGTAGCGTGGTTTCCGGTTCCGATACCCATGTGAAGGGCCTTCCCA
RH 4 CC      AAAAAGCATGGAAAGAATGATTAATTGAATTAATTGTAGCGTGGTTTCCGGTTCCGATACCCATGTGAAGGGCCTTCCCA
RH 5 CC      AAAAAGCATGGAAAGAATGATTAATTGAATTAATTGTAGCGTGGTTTCCGGTTCCGATACCCATGTGAAGGGCCTTCCCA
RH 6 CC      AAAAAGCATGGAAAGAATGATTAATTGAATTAATTGTAGCGTGGTTTCCGGTTCCGATACCCATGTGAAGGGCCTTCCCA
RH 7 CC      AAAAAGCATGGAAAGAATGATTAATTGAATTAATTGTAGCGTGGTTTCCGGTTCCGATACCCATGTGAAGGGCCTTCCCA
RH 9 CC      AAAAAGCATGGAAAGAATGATTAATTGAATTAATTGTAGCGTGGTTTCCGGTTCCGATACCCATGTGAAGGGCCTTCCCA
RH 10 CC     AAAAAGCATGGAAAGAATGATTAATTGAATTAATTGTAGCGTGGTTTCCGGTTCCGATACCCATGTGAAGGGCCTTCCCA
WSA1 1 2 CC  AAAAAGCATGGAAAGAATGATTAATTGAATTAATTGTAGCGTGGTTTCCGGTTCCGATACCCATGTGAAGGGCCTTCCCA
WSA1 1 4 CC  AAAAAGCATGGAAAGAATGATTAATTGAATTAATTGTAGCGTGGTTTCCGGTTCCGATACCCATGTGAAGGGCCTTCCCA
WSA1 1 5 CC  AAAAAGCATGGAAAGAATGATTAATTGAATTAATTGTAGCGTGGTTTCCGGTTCCGATACCCATGTGAAGGGCCTTCCCA
WSA1 1 6 CC  AAAAAGCATGGAAAGAATGATTAATTGAATTAATTGTAGCGTGGTTTCCGGTTCCGATACCCATGTGAAGGGCCTTCCCA
WSA1 1 9 CC  AAAAAGCATGGAAAGAATGATTAATTGAATTAATTGTAGCGTGGTTTCCGGTTCCGATACCCATGTGAAGGGCCTTCCCA
WSA1 1 10 CC AAAAAGCATGGAAAGAATGATTAATTGAATTAATTGTAGCGTGGTTTCCGGTTCCGATACCCATGTGAAGGGCCTTCCCA
WSA1 1 11 CC AAAAAGCATGGAAAGAATGATTAATTGAATTAATTGTAGCGTGGTTTCCGGTTCCGATACCCATGTGAAGGGCCTTCCCA
WSA1 1 12 CC AAAAAGCATGGAAAGAATGATTAATTGAATTAATTGTAGCGTGGTTTCCGGTTCCGATACCCATGTGAAGGGCCTTCCCA
WSA1 1 16 CC AAAAAGCATGGAAAGAATGATTAATTGAATTAATTGTAGCGTGGTTTCCGGTTCCGATACCCATGTGAAGGGCCTTCCCA
WSA1 1 17 CC AAAAAGCATGGAAAGAATGATTAATTGAATTAATTGTAGCGTGGTTTCCGGTTCCGATACCCATGTGAAGGGCCTTCCCA
WSA1 1 18 CC AAAAAGCATGGAAAGAATGATTAATTGAATTAATTGTAGCGTGGTTTCCGGTTCCGATACCCATGTGAAGGGCCTTCCCA
WSA1 1 19 CC AAAAAGCATGGAAAGAATGATTAATTGAATTAATTGTAGCGTGGTTTCCGGTTCCGATACCCATGTGAAGGGCCTTCCCA
WSA1 1 20 CC AAAAAGCATGGAAAGAATGATTAATTGAATTAATTGTAGCGTGGTTTCCGGTTCCGATACCCATGTGAAGGGCCTTCCCA
WSA1 2 2 CC  AAAAAGCATGGAAAGAATGATTAATTGAATTAATTGTAGCGTGGTTTCCGGTTCCGATACCCATGTGAAGGGCCTTCCCA
WSA1 2 3 CC  AAAAAGCATGGAAAGAATGATTAATTGAATTAATTGTAGCGTGGTTTCCGGTTCCGATACCCATGTGAAGGGCCTTCCCA
WSA1 2 4 CC  AAAAAGCATGGAAAGAATGATTAATTGAATTAATTGTAGCGTGGTTTCCGGTTCCGATACCCATGTGAAGGGCCTTCCCA
WSA1 2 5 CC  AAAAAGCATGGAAAGAATGATTAATTGAATTAATTGTAGCGTGGTTTCCGGTTCCGATACCCATGTGAAGGGCCTTCCCA
WSA1 2 7 CC  AAAAAGCATGGAAAGAATGATTAATTGAATTAATTGTAGCGTGGTTTCCGGTTCCGATACCCATGTGAAGGGCCTTCCCA
WSA2 1 CC    AAAAAGCATGGAAAGAATGATTAATTGAATTAATTGTAGCGTGGTTTCCGGTTCCGATACCCATGTGAAGGGCCTTCCCA
WSA2 2 CC    AAAAAGCATGGAAAGAATGATTAATTGAATTAATTGTAGCGTGGTTTCCGGTTCCGATACCCATGTGAAGGGCCTTCCCA
```

1 10 20 30 40 50 60 70 80

WSA2 3 CC	AAAAAGCATGGAAAGAATGATTAATTGAATTAATTGTAGCGTGGTTTCCGGTTCCGATACCCATGTGAAGGGCCTTCCCA
Cr 4 CC	AAAAAGCATGGAAAGAATGATTAATTGAATTAATTGTAGCGTGGTTTCCGGTTCCGATACCCATGTGAAGGGCCTTCCCA
Cr CL2004 CC	AAAAAGCATGGAAAGAATGATTAATTGAATTAATTGTAGCGTGGTTTCCGGTTCCGATACCCATGTGAAGGGCCTTCCCA
Cr2004 1 CC	AAAAAGCATGGAAAGAATGATTAATTGAATTAATTGTAGCGTGGTTTCCGGTTCCGATACCCATGTGAAGGGCCTTCCCA
Cr2004 2 CC	AAAAAGCATGGAAAGAATGATTAATTGAATTAATTGTAGCGTGGTTTCCGGTTCCGATACCCATGTGAAGGGCCTTCCCA
Cr2004 3 CC	AAAAAGCATGGAAAGAATGATTAATTGAATTAATTGTAGCGTGGTTTCCGGTTCCGATACCCATGTGAAGGGCCTTCCCA
Cr2004 4 CC	AAAAAGCATGGAAAGAATGATTAATTGAATTAATTGTAGCGTGGTTTCCGGTTCCGATACCCATGTGAAGGGCCTTCCCA
Cr2004 8 CC	AAAAAGCATGGAAAGAATGATTAATTGAATTAATTGTAGCGTGGTTTCCGGTTCCGATACCCATGTGAAGGGCCTTCCCA
Cr2004 10 CC	AAAAAGCATGGAAAGAATGATTAATTGAATTAATTGTAGCGTGGTTTCCGGTTCCGATACCCATGTGAAGGGCCTTCCCA
Cr2004 11 CC	AAAAAGCATGGAAAGAATGATTAATTGAATTAATTGTAGCGTGGTTTCCGGTTCCGATACCCATGTGAAGGGCCTTCCCA
Cr2004 12 CC	AAAAAGCATGGAAAGAATGATTAATTGAATTAATTGTAGCGTGGTTTCCGGTTCCGATACCCATGTGAAGGGCCTTCCCA
Cr2004 13 CC	AAAAAGCATGGAAAGAATGATTAATTGAATTAATTGTAGCGTGGTTTCCGGTTCCGATACCCATGTGAAGGGCCTTCCCA
Cr2004 14 CC	AAAAAGCATGGAAAGAATGATTAATTGAATTAATTGTAGCGTGGTTTCCGGTTCCGATACCCATGTGAAGGGCCTTCCCA
Nep2004 3 CC	AAAAAGCATGGAAAGAATGATTAATTGAATTAATTGTAGCGTGGTTTCCGGTTCCGATACCCATGTGAAGGGCCTTCCCA
Nep2004 5 CC	AAAAAGCATGGAAAGAATGATTAATTGAATTAATTGTAGCGTGGTTTCCGGTTCCGATACCCATGTGAAGGGCCTTCCCA
Nep2004 6 CC	AAAAAGCATGGAAAGAATGATTAATTGAATTAATTGTAGCGTGGTTTCCGGTTCCGATACCCATGTGAAGGGCCTTCCCA
Nep2004 8 CC	AAAAAGCATGGAAAGAATGATTAATTGAATTAATTGTAGCGTGGTTTCCGGTTCCGATACCCATGTGAAGGGCCTTCCCA
Nep2004 9 CC	AAAAAGCATGGAAAGAATGATTAATTGAATTAATTGTAGCGTGGTTTCCGGTTCCGATACCCATGTGAAGGGCCTTCCCA
Nep2004 10 CC	AAAAAGCATGGAAAGAATGATTAATTGAATTAATTGTAGCGTGGTTTCCGGTTCCGATACCCATGTGAAGGGCCTTCCCA
Nep2004 11 CC	AAAAAGCATGGAAAGAATGATTAATTGAATTAATTGTAGCGTGGTTTCCGGTTCCGATACCCATGTGAAGGGCCTTCCCA
Nep2004 12 CC	AAAAAGCATGGAAAGAATGATTAATTGAATTAATTGTAGCGTGGTTTCCGGTTCCGATACCCATGTGAAGGGCCTTCCCA
Nep2004 14 CC	AAAAAGCATGGAAAGAATGATTAATTGAATTAATTGTAGCGTGGTTTCCGGTTCCGATACCCATGTGAAGGGCCTTCCCA
Nep2004 16 CC	AAAAAGCATGGAAAGAATGATTAATTGAATTAATTGTAGCGTGGTTTCCGGTTCCGATACCCATGTGAAGGGCCTTCCCA
Nep2004 17 CC	AAAAAGCATGGAAAGAATGATTAATTGAATTAATTGTAGCGTGGTTTCCGGTTCCGATACCCATGTGAAGGGCCTTCCCA
Nep2004 18 CC	AAAAAGCATGGAAAGAATGATTAATTGAATTAATTGTAGCGTGGTTTCCGGTTCCGATACCCATGTGAAGGGCCTTCCCA
Nep2004 19 CC	AAAAAGCATGGAAAGAATGATTAATTGAATTAATTGTAGCGTGGTTTCCGGTTCCGATACCCATGTGAAGGGCCTTCCCA
Nep2004 20 CC	AAAAAGCATGGAAAGAATGATTAATTGAATTAATTGTAGCGTGGTTTCCGGTTCCGATACCCATGTGAAGGGCCTTCCCA
Nep2004 21 CC	AAAAAGCATGGAAAGAATGATTAATTGAATTAATTGTAGCGTGGTTTCCGGTTCCGATACCCATGTGAAGGGCCTTCCCA
Nep2004 22 CC	AAAAAGCATGGAAAGAATGATTAATTGAATTAATTGTAGCGTGGTTTCCGGTTCCGATACCCATGTGAAGGGCCTTCCCA
Nep2004 23 CC	AAAAAGCATGGAAAGAATGATTAATTGAATTAATTGTAGCGTGGTTTCCGGTTCCGATACCCATGTGAAGGGCCTTCCCA
Nep2004 24 CC	AAAAAGCATGGAAAGAATGATTAATTGAATTAATTGTAGCGTGGTTTCCGGTTCCGATACCCATGTGAAGGGCCTTCCCA
Nep2004 25 CC	AAAAAGCATGGAAAGAATGATTAATTGAATTAATTGTAGCGTGGTTTCCGGTTCCGATACCCATGTGAAGGGCCTTCCCA
Nep2004 26 CC	AAAAAGCATGGAAAGAATGATTAATTGAATTAATTGTAGCGTGGTTTCCGGTTCCGATACCCATGTGAAGGGCCTTCCCA
Nep2004 27 CC	AAAAAGCATGGAAAGAATGATTAATTGAATTAATTGTAGCGTGGTTTCCGGTTCCGATACCCATGTGAAGGGCCTTCCCA
Nep2004 29 CC	AAAAAGCATGGAAAGAATGATTAATTGAATTAATTGTAGCGTGGTTTCCGGTTCCGATACCCATGTGAAGGGCCTTCCCA
Nep2004 30 CC	AAAAAGCATGGAAAGAATGATTAATTGAATTAATTGTAGCGTGGTTTCCGGTTCCGATACCCATGTGAAGGGCCTTCCCA
Poc 3 CC	AAAAAGCATGGAAAGAATGATTAATTGAATTAATTGTAGCGTGGTTTCCGGTTCCGATACCCATGTGAAGGGCCTTCCCA
Poc 5 CC	AAAAAGCATGGAAAGAATGATTAATTGAATTAATTGTAGCGTGGTTTCCGGTTCCGATACCCATGTGAAGGGCCTTCCCA
Poc 6 CC	AAAAAGCATGGAAAGAATGATTAATTGAATTAATTGTAGCGTGGTTTCCGGTTCCGATACCCATGTGAAGGGCCTTCCCA
Poc 7 CC	AAAAAGCATGGAAAGAATGATTAATTGAATTAATTGTAGCGTGGTTTCCGGTTCCGATACCCATGTGAAGGGCCTTCCCA
Poc 8 CC	AAAAAGCATGGAAAGAATGATTAATTGAATTAATTGTAGCGTGGTTTCCGGTTCCGATACCCATGTGAAGGGCCTTCCCA
Poc 10 CC	AAAAAGCATGGAAAGAATGATTAATTGAATTAATTGTAGCGTGGTTTCCGGTTCCGATACCCATGTGAAGGGCCTTCCCA
Poc 11 CC	AAAAAGCATGGAAAGAATGATTAATTGAATTAATTGTAGCGTGGTTTCCGGTTCCGATACCCATGTGAAGGGCCTTCCCA
Poc 12 CC	AAAAARCATGGAAAGAATGATTAATTGAATTAATTGTAGCGTGGTTTCCGGTTCCGATACCCATGTGAAGGGCCTTCCCA
Poc 13 CC	AAAAAGCATGGAAAGAATGATTAATTGAATTAATTGTAGCGTGGTTTCCGGTTCCGATACCCATGTGAAGGGCCTTCCCA
Poc 14 CC	AAAAAGCATGGAAAGAATGATTAATTGAATTAATTGTAGCGTGGTTTCCGGTTCCGATACCCATGTGAAGGGCCTTCCCA
Poc 15 CC	AAAAAGCATGGAAAGAATGATTAATTGAATTAATTGTAGCGTGGTTTCCGGTTCCGATACCCATGTGAAGGGCCTTCCCA
Poc 16 CC	AAAAAGCATGGAAAGAATGATTAATTGAATTAATTGTAGCGTGGTTTCCGGTTCCGATACCCATGTGAAGGGCCTTCCCA
Poc 17 CC	AAAAAGCATGGAAAGAATGATTAATTGAATTAATTGTAGCGTGGTTTCCGGTTCCGATACCCATGTGAAGGGCCTTCCCA
Poc 21 CC	AAAAAGCATGGAAAGAATGATTAATTGAATTAATTGTAGCGTGGTTTCCGGTTCCGATACCCATGTGAAGGGCCTTCCCA
Poc 22 CC	AAAAAGCATGGAAAGAATGATTAATTGAATTAATTGTAGCGTGGTTTCCGGTTCCGATACCCATGTGAAGGGCCTTCCCA

Sample	Sequence (positions 1–80)
Sipp1 CL1 CC	AAAAAGCATGGAAAGAATGATTAATTGAATTAATTGTAGCGTGGTTTCCGGTTCCGATACCCATGTGAAGGGCCTTCCCA
Sipp 1 Cl2 CC	AAAAAGCATGGAAAGAATGATTAATTGAATTAATTGTAGCGTGGTTTCCGGTTCCGATACCCATGTGAAGGGCCTTCCCA
Sipp4 CL1 CC	AAAAAGCATGGAAAGAATGATTAATTGAATTAATTGTAGCGTGGTTTCCGGTTCCGATACCCATGTGAAGGGCCTTCCCA
Sipp04 2 1 CC	AAAAAGCATGGAAAGAATGATTAATTGAATTAATTGTAGCGTGGTTTCCGGTTCCGATACCCATGTGAAGGGCCTTCCCA
Sipp04 2 5 CC	AAAAAGCATGGAAAGAATGATTAATTGAATTAATTGTAGCGTGGTTTCCGGTTCCGATACCCATGTGAAGGGCCTTCCCA
Sipp04 2 7 CC	AAAAAGCATGGAAAGAATGATTAATTGAATTAATTGTAGCGTGGTTTCCGGTTCCGATACCCATGTGAAGGGCCTTCCCA
Sipp04 2 9 CC	AAAAAGCATGGAAAGAATGATTAATTGAATTAATTGTAGCGTGGTTTCCGGTTCCGATACCCATGTGAAGGGCCTTCCCA
Sipp04 2 11 CC	AAAAAGCATGGAAAGAATGATTAATTGAATTAATTGTAGCGTGGTTTCCGGTTCCGATACCCATGTGAAGGGCCTTCCCA
Sipp04 2 14 CC	AAAAAGCATGGAAAGAATGATTAATTGAATTAATTGTAGCGTGGTTTCCGGTTCCGATACCCATGTGAAGGGCCTTCCCA
Sipp04 2 17 CC	AAAAAGCATGGAAAGAATGATTAATTGAATTAATTGTAGCGTGGTTTCCGGTTCCGATACCCATGTGAAGGGCCTTCCCA
Sipp04 3 1 CC	AAAAAGCATGGAAAGAATGATTAATTGAATTAATTGTAGCGTGGTTTCCGGTTCCGATACCCATGTGAAGGGCCTTCCCA
Sipp04 3 3 CC	AAAAAGCATGGAAAGAATGATTAATTGAATTAATTGTAGCGTGGTTTCCGGTTCCGATACCCATGTGAAGGGCCTTCCCA
Sipp04 3 5 CC	AAAAAGCATGGAAAGAATGATTAATTGAATTAATTGTAGCGTGGTTTCCGGTTCCGATACCCATGTGAAGGGCCTTCCCA
Sipp04 3 6 CC	AAAAAGCATGGAAAGAATGATTAATTGAATTAATTGTAGCGTGGTTTCCGGTTCCGATACCCATGTGAAGGGCCTTCCCA
Sipp 13 15 CC	AAAAAGCATGGAAAGAATGATTAATTGAATTAATTGTAGCGTGGTTTCCGGTTCCGATACCCATGTGAAGGGCCTTCCCA
Sipp 14 1 CC	AAAAAGCATGGAAAGAATGATTAATTGAATTAATTGTAGCGTGGTTTCCGGTTCCGATACCCATGTGAAGGGCCTTCCCA
Sipp 14 2 CC	AAAAAGCATGGAAAGAATGATTAATTGAATTAATTGTAGCGTGGTTTCCGGTTCCGATACCCATGTGAAGGGCCTTCCCA
Sipp 14 3 CC	AAAAAGCATGGAAAGAATGATTAATTGAATTAATTGTAGCGTGGTTTCCGGTTCCGATACCCATGTGAAGGGCCTTCCCA
Sipp 14 4 CC	AAAAAGCATGGAAAGAATGATTAATTGAATTAATTGTAGCGTGGTTTCCGGTTCCGATACCCATGTGAAGGGCCTTCCCA
Sipp 14 5 CC	AAAAAGCATGGAAAGAATGATTAATTGAATTAATTGTAGCGTGGTTTCCGGTTCCGATACCCATGTGAAGGGCCTTCCCA
Sipp 14 6 CC	AAAAAGCATGGAAAGAATGATTAATTGAATTAATTGTAGCGTGGTTTCCGGTTCCGATACCCATGTGAAGGGCCTTCCCA
Sipp 14 7 CC	AAAAAGCATGGAAAGAATGATTAATTGAATTAATTGTAGCGTGGTTTCCGGTTCCGATACCCATGTGAAGGGCCTTCCCA
Sipp 14 8 CC	AAAAAGCATGGAAAGAATGATTAATTGAATTAATTGTAGCGTGGTTTCCGGTTCCGATACCCATGTGAAGGGCCTTCCCA
Sipp 14 9 CC	AAAAAGCATGGAAAGAATGATTAATTGAATTAATTGTAGCGTGGTTTCCGGTTCCGATACCCATGTGAAGGGCCTTCCCA
Sipp 14 10 CC	AAAAAGCATGGAAAGAATGATTAATTGAATTAATTGTAGCGTGGTTTCCGGTTCCGATACCCATGTGAAGGGCCTTCCCA
Sipp 14 11 CC	AAAAAGCATGGAAAGAATGATTAATTGAATTAATTGTAGCGTGGTTTCCGGTTCCGATACCCATGTGAAGGGCCTTCCCA
Sipp 14 12 CC	AAAAAGCATGGAAAGAATGATTAATTGAATTAATTGTAGCGTGGTTTCCGGTTCCGATACCCATGTGAAGGGCCTTCCCA
Sipp 14 13 CC	AAAAAGCATGGAAAGAATGATTAATTGAATTAATTGTAGCGTGGTTTCCGGTTCCGATACCCATGTGAAGGGCCTTCCCA
Sipp 14 14 CC	AAAAAGCATGGAAAGAATGATTAATTGAATTAATTGTAGCGTGGTTTCCGGTTCCGATACCCATGTGAAGGGCCTTCCCA
Sipp 14 15 CC	AAAAAGCATGGAAAGAATGATTAATTGAATTAATTGTAGCGTGGTTTCCGGTTCCGATACCCATGTGAAGGGCCTTCCCA
Sipp 15 1 CC	AAAAAGCATGGAAAGAATGATTAATTGAATTAATTGTAGCGTGGTTTCCGGTTCCGATACCCATGTGAAGGGCCTTCCCA
Sipp 15 2 CC	AAAAAGCATGGAAAGAATGATTAATTGAATTAATTGTAGCGTGGTTTCCGGTTCCGATACCCATGTGAAGGGCCTTCCCA
Sipp 15 3 CC	AAAAAGCATGGAAAGAATGATTAATTGAATTAATTGTAGCGTGGTTTCCGGTTCCGATACCCATGTGAAGGGCCTTCCCA
Sipp 15 4 CC	AAAAAGCATGGAAAGAATGATTAATTGAATTAATTGTAGCGTGGTTTCCGGTTCCGATACCCATGTGAAGGGCCTTCCCA
Sipp 15 5 CC	AAAAAGCATGGAAAGAATGATTAATTGAATTAATTGTAGCGTGGTTTCCGGTTCCGATACCCATGTGAAGGGCCTTCCCA
Sipp 15 6 CC	AAAAAGCATGGAAAGAATGATTAATTGAATTAATTGTAGCGTGGTTTCCGGTTCCGATACCCATGTGAAGGGCCTTCCCA
Sipp 15 7 CC	AAAAAGCATGGAAAGAATGATTAATTGAATTAATTGTAGCGTGGTTTCCGGTTCCGATACCCATGTGAAGGGCCTTCCCA
Sipp 15 8 CC	AAAAAGCATGGAAAGAATGATTAATTGAATTAATTGTAGCGTGGTTTCCGGTTCCGATACCCATGTGAAGGGCCTTCCCA
Sipp 15 9 CC	AAAAAGCATGGAAAGAATGATTAATTGAATTAATTGTAGCGTGGTTTCCGGTTCCGATACCCATGTGAAGGGCCTTCCCA
Sipp 15 10 CC	AAAAAGCATGGAAAGAATGATTAATTGAATTAATTGTAGCGTGGTTTCCGGTTCCGATACCCATGTGAAGGGCCTTCCCA
Sipp 15 11 CC	AAAAAGCATGGAAAGAATGATTAATTGAATTAATTGTAGCGTGGTTTCCGGTTCCGATACCCATGTGAAGGGCCTTCCCA
Sipp 15 12 CC	AAAAAGCATGGAAAGAATGATTAATTGAATTAATTGTAGCGTGGTTTCCGGTTCCGATACCCATGTGAAGGGCCTTCCCA
Sipp 15 13 CC	AAAAAGCATGGAAAGAATGATTAATTGAATTAATTGTAGCGTGGTTTCCGGTTCCGATACCCATGTGAAGGGCCTTCCCA
Sipp 15 14 CC	AAAAAGCATGGAAAGAATGATTAATTGAATTAATTGTAGCGTGGTTTCCGGTTCCGATACCCATGTGAAGGGCCTTCCCA
Sipp 15 15 CC	AAAAAGCATGGAAAGAATGATTAATTGAATTAATTGTAGCGTGGTTTCCGGTTCCGATACCCATGTGAAGGGCCTTCCCA
Sipp 16 1 CS	AAAAAGCATGGAAAKAATGATTAATTGAATTAWTTKTAGCGTGGTTTCCGRTTCCGATACCCAWGTGAAGGGCCTTCCCA
Sipp 16 2 CS	AAAAAGCATGGAAAKAATGATTAATTGAATTAWTTKTAGCGTGGTTTCCGRTTCCGATACCCAWGTGAAGGGCCTTCCCA
Sipp 16 3 CS	AAAAAGCATGGAAAKAATGATTAATTGAATTAWTTKTAGCGTGGTTTCCGRTTCCGATACCCAWGTGAAGGGCCTTCCCA
Sipp 16 8 CC	AAAAAGCATGGAAAGAATGATTAATTGAATTAATTGTAGCGTGGTTTCCGGTTCCGATACCCATGTGAAGGGCCTTCCCA
NJ CL2 CC	AAAAAGCATGGAAAGAATGATTAATTGAATTAATTGTAGCGTGGTTTCCGGTTCCGATACCCATGTGAAGGGCCTTCCCA
NJ CL3 CC	AAAAAGCATGGAAAGAATGATTAATTGAATTAATTGTAGCGTGGTTTCCGGTTCCGATACCCATGTGAAGGGCCTTCCCA

```
             1        10        20        30        40        50        60        70        80
NJM 1 4 CC   AAAAAGCATGGAAAGAATGATTAATTGAATTAATTGTAGCGTGGTTTCCGGTTCCGATACCCATGTGAAGGGCCTTCCCA
NJM 1 5 CC   AAAAAGCATGGAAAGAATGATTAATTGAATTAATTGTAGCGTGGTTTCCGGTTCCGATACCCATGTGAAGGGCCTTCCCA
NJM 1 8 CC   AAAAAGCATGGAAAGAATGATTAATTGAATTAATTGTAGCGTGGTTTCCGGTTCCGATACCCATGTGAAGGGCCTTCCCA
NJM 1 9 CC   AAAAAGCATGGAAAGAATGATTAATTGAATTAATTGTAGCGTGGTTTCCGGTTCCGATACCCATGTGAAGGGCCTTCCCA
NJM 1 10 CC  AAAAAGCATGGAAAGAATGATTAATTGAATTAATTGTAGCGTGGTTTCCGGTTCCGATACCCATGTGAAGGGCCTTCCCA
NJM 1 11 CC  AAAAAGCATGGAAAGAATGATTAATTGAATTAATTGTAGCGTGGTTTCCGGTTCCGATACCCATGTGAAGGGCCTTCCCA
Rh1 CS       AAAAAGCATGGAAAKAATGATTAATTGAATTAWTTKTAGCGTGGTTTCCGRTTCCGATACCCAWGTGAAGGGCCTTCCCA
Rh3 SS       AAAAAGCATGGAAATAATGATTAATTGAATTATTTTTAGCGTGGTTTCCGATTCCGATACCCAAGTGAAGGGCCTTCCCA
Rh5 SS       AAAAAGCATGGAAATAATGATTAATTGAATTATTTTTAGCGTGGTTTCCGATTCCGATACCCAAGTGAAGGGCCTTCCCA
Rh6 CS       AAAAAGCATGGAAAKAATGATTAATTGAATTAWTTKTAGCGTGGTTTCCGRTTCCGATACCCAWGTGAAGGGCCTTCCCA
Rh8 CS       AAAAAGCATGGAAAKAATGATTAATTGAATTAWTTKTAGCGTGGTTTCCGRTTCCGATACCCAWGTGAAGGGCCTTCCCA
Rh9 CC       AAAAAGCATGGAAAGAATGATTAATTGAATTAATTGTAGCGTGGTTTCCGGTTCCGATACCCATGTGAAGGGCCTTCCCA
Rh10 CC      AAAAAGCATGGAAAGAATGATTAATTGAATTAATTGTAGCGTGGTTTCCGGTTCCGATACCCATGTGAAGGGCCTTCCCA
Rh11 CS      AAAAAGCATGGAAAKAATGATTAATTGAATTAWTTKTAGCGTGGTTTCCGRTTCCGATACCCAWGTGAAGGGCCTTCCCA
Rh12 CS      AAAAAGCATGGAAAKAATGATTAATTGAATTAWTTKTAGCGTGGTTTCCGRTTCCGATACCCAWGTGAAGGGCCTTCCCA
Rh13 CC      AAAAAGCATGGAAAGAATGATTAATTGAATTAATTGTAGCGTGGTTTCCGGTTCCGATACCCATGTGAAGGGCCTTCCCA
Rh14 SS      AAAAAGCATGGAAATAATGATTAATTGAATTATTTTTAGCGTGGTTTCCGATTCCGATACCCAAGTGAAGGGCCTTCCCA
Rh15 SS      AAAAAGCATGGAAATAATGATTAATTGAATTATTTTTAGCGTGGTTTCCGATTCCGATACCCAAGTGAAGGGCCTTCCCA
Rh16 CC      AAAAAGCATGGAAAGAATGATTAATTGAATTAATTGTAGCGTGGTTTCCGGTTCCGATACCCATGTGAAGGGCCTTCCCA
Rh17 CS      AAAAAGCATGGAAAKAATGATTAATTGAATTAWTTKTAGCGTGGTTTCCGRTTCCGATACCCAWGTGAAGGGCCTTCCCA
Rh18 CS      AAAAAGCATGGAAAKAATGATTAATTGAATTAWTTKTAGCGTGGTTTCCGRTTCCGATACCCAWGTGAAGGGCCTTCCCA
Rh19 CC      AAAAAGCATGGAAAGAATGATTAATTGAATTAATTGTAGCGTGGTTTCCGGTTCCGATACCCATGTGAAGGGCCTTCCCA
Rh21 CC      AAAAAGCATGGAAAGAATGATTAATTGAATTAATTGTAGCGTGGTTTCCGGTTCCGATACCCATGTGAAGGGCCTTCCCA
Rh23 CS      AAAAAGCATGGAAAKAATGATTAATTGAATTAWTTKTAGCGTGGTTTCCGRTTCCGATACCCAWGTGAAGGGCCTTCCCA
Rh24 CS      AAAAAGCATGGAAAKAATGATTAATTGAATTAWTTKTAGCGTGGTTTCCGRTTCCGATACCCAWGTGAAGGGCCTTCCCA
Rh1-1 SS     AAAAAGCATGGAAATAATGATTAATTGAATTATTTTTAGCGTGGTTTCCGATTCCGATACCCAAGTGAAGGGCCTTCCCA
Rh1-2 CS     AAAAAGCATGGAAAKAATGATTAATTGAATTAWTTKTAGCGTGGTTTCCGRTTCCGATACCCAWGTGAAGGGCCTTCCCA
Rh1-3 CC     AAAAAGCATGGAAAGAATGATTAATTGAATTAATTGTAGCGTGGTTTCCGGTTCCGATACCCATGTGAAGGGCCTTCCCA
Rh1-6 CC     AAAAAGCATGGAAAGAATGATTAATTGAATTAATTGTAGCGTGGTTTCCGGTTCCGATACCCATGTGAAGGGCCTTCCCA
Rh1-7 CC     AAAAAGCATGGAAAGAATGATTAATTGAATTAATTGTAGCGTGGTTTCCGGTTCCGATACCCATGTGAAGGGCCTTCCCA
Rh1-8 CS     AAAAAGCATGGAAAKAATGATTAATTGAATTAWTTKTAGCGTGGTTTCCGRTTCCGATACCCAWGTGAAGGGCCTTCCCA
Rh1-9 SS     AAAAAGCATGGAAATAATGATTAATTGAATTATTTTTAGCGTGGTTTCCGATTCCGATACCCAAGTGAAGGGCCTTCCCA
Rh1-10 CC    AAAAAGCATGGAAAGAATGATTAATTGAATTAATTGTAGCGTGGTTTCCGGTTCCGATACCCATGTGAAGGGCCTTCCCA
Rh1-11 CS    AAAAAGCATGGAAAKAATGATTAATTGAATTAWTTKTAGCGTGGTTTCCGRTTCCGATACCCAWGTGAAGGGCCTTCCCA
Rh1-13 CS    AAAAAGCATGGAAAKAATGATTAATTGAATTAWTTKTAGCGTGGTTTCCGRTTCCGATACCCAWGTGAAGGGCCTTCCCA
Rh1-14 CS    AAAAAGCATGGAAAKAATGATTAATTGAATTAWTTKTAGCGTGGTTTCCGRTTCCGATACCCAWGTGAAGGGCCTTCCCA
Rh1-15 CS    AAAAAGCATGGAAAKAATGATTAATTGAATTAWTTKTAGCGTGGTTTCCGRTTCCGATACCCAWGTGAAGGGCCTTCCCA
Rh1-17 CS    AAAAAGCATGGAAAKAATGATTAATTGAATTAWTTKTAGCGTGGTTTCCGRTTCCGATACCCAWGTGAAGGGCCTTCCCA
Rh1-18 CC    AAAAAGCATGGAAAGAATGATTAATTGAATTAATTGTAGCGTGGTTTCCGGTTCCGATACCCATGTGAAGGGCCTTCCCA
Rh1- 19 SS   AAAAAGCATGGAAATAATGATTAATTGAATTATTTTTAGCGTGGTTTCCGATTCCGATACCCAAGTGAAGGGCCTTCCCA
Rh1-20 CC    AAAAAGCATGGAAAGAATGATTAATTGAATTAATTGTAGCGTGGTTTCCGGTTCCGATACCCATGTGAAGGGCCTTCCCA
Rh1-21 CS    AAAAAGCATGGAAAKAATGATTAATTGAATTAWTTKTAGCGTGGTTTCCGRTTCCGATACCCAWGTGAAGGGCCTTCCCA
Rh1-23 CC    AAAAAGCATGGAAAGAATGATTAATTGAATTAATTGTAGCGTGGTTTCCGGTTCCGATACCCATGTGAAGGGCCTTCCCA
Rh1-24 CC    AAAAAGCATGGAAAGAATGATTAATTGAATTAATTGTAGCGTGGTTTCCGGTTCCGATACCCATGTGAAGGGCCTTCCCA
Rh1-27 CC    AAAAAGCATGGAAAGAATGATTAATTGAATTAATTGTAGCGTGGTTTCCGGTTCCGATACCCATGTGAAGGGCCTTCCCA
Rh1-28 SS    AAAAAGCATGGAAATAATGATTAATTGAATTATTTTTAGCGTGGTTTCCGATTCCGATACCCAAGTGAAGGGCCTTCCCA
Rh1- 30 CC   AAAAAGCATGGAAAGAATGATTAATTGAATTAATTGTAGCGTGGTTTCCGGTTCCGATACCCATGTGAAGGGCCTTCCCA
Rh1-31 CS    AAAAAGCATGGAAAKAATGATTAATTGAATTAWTTKTAGCGTGGTTTCCGRTTCCGATACCCAWGTGAAGGGCCTTCCCA
Rh1-32 CC    AAAAAGCATGGAAAGAATGATTAATTGAATTAATTGTAGCGTGGTTTCCGGTTCCGATACCCATGTGAAGGGCCTTCCCA
Rh1-33 CC    AAAAAGCATGGAAAGAATGATTAATTGAATTAATTGTAGCGTGGTTTCCGGTTCCGATACCCATGTGAAGGGCCTTCCCA
Rh1-34 CC    AAAAAGCATGGAAAGAATGATTAATTGAATTAATTGTAGCGTGGTTTCCGGTTCCGATACCCATGTGAAGGGCCTTCCCA
```

Rh1-35 CS	AAAAAGCATGGAAAKAATGATTAATTGAATTAWTTKTAGCGTGGTTTCCGRTTCCGATACCCAWGTGAAGGGCCTTCCCA
Rh1-36 CS	AAAAAGCATGGAAAKAATGATTAATTGAATTAWTTKTAGCGTGGTTTCCGRTTCCGATACCCAWGTGAAGGGCCTTCCCA
Rh1-37 CS	AAAAAGCATGGAAAKAATGATTAATTGAATTAWTTKTAGCGTGGTTTCCGRTTCCGATACCCAWGTGAAGGGCCTTCCCA
Rh1-38 CS	AAAAAGCATGGAAAKAATGATTAATTGAATTAWTTKTAGCGTGGTTTCCGRTTCCGATACCCAWGTGAAGGGCCTTCCCA
Rh1-39 SS	AAAAAGCATGGAAATAATGATTAATTGAATTATTTTTAGCGTGGTTTCCGATTCCGATACCCAAGTGAAGGGCCTTCCCA
Rh1-40 CC	AAAAAGCATGGAAAGAATGATTAATTGAATTAATTGTAGCGTGGTTTCCGGTTCCGATACCCATGTGAAGGGCCTTCCCA
Rh1-41 CC	AAAAAGCATGGAAAGAATGATTAATTGAATTAATTGTAGCGTGGTTTCCGGTTCCGATACCCATGTGAAGGGCCTTCCCA
Rh1-43 SS	AAAAAGCATGGAAATAATGATTAATTGAATTATTTTTAGCGTGGTTTCCGATTCCGATACCCAAGTGAAGGGCCTTCCCA
Rh1-44 CC	AAAAAGCATGGAAAGAATGATTAATTGAATTAATTGTAGCGTGGTTTCCGGTTCCGATACCCATGTGAAGGGCCTTCCCA
Rh1-47 CS	AAAAAGCATGGAAAKAATGATTAATTGAATTAWTTKTAGCGTGGTTTCCGRTTCCGATACCCAWGTGAAGGGCCTTCCCA
Rh1-49 CS	AAAAAGCATGGAAAKAATGATTAATTGAATTAWTTKTAGCGTGGTTTCCGRTTCCGATACCCAWGTGAAGGGCCTTCCCA
Rh1-52 CS	AAAAAGCATGGAAAKAATGATTAATTGAATTAWTTKTAGCGTGGTTTCCGRTTCCGATACCCAWGTGAAGGGCCTTCCCA
Rh1-53 CS	AAAAAGCATGGAAAKAATGATTAATTGAATTAWTTKTAGCGTGGTTTCCGRTTCCGATACCCAWGTGAAGGGCCTTCCCA
Rh1-54 CC	AAAAAGCATGGAAAGAATGATTAATTGAATTAATTGTAGCGTGGTTTCCGGTTCCGATACCCATGTGAAGGGCCTTCCCA
Rh1-58 CS	AAAAAGCATGGAAAKAATGATTAATTGAATTAWTTKTAGCGTGGTTTCCGRTTCCGATACCCAWGTGAAGGGCCTTCCCA
Rh1-60 SS	AAAAAGCATGGAAATAATGATTAATTGAATTATTTTTAGCGTGGTTTCCGATTCCGATACCCAAGTGAAGGGCCTTCCCA
Bar1a 3 CC	AAAAARCATGGAAAGAATGATTAATTGAATTAATTGTAGCGTGGTTTCCGGTTCCGATACCCATGTGAAGGGCCTTCCCA
Bar1a 4 CC	AAAAAACATGGAAAGAATGATTAATTGAATTAATTGTAGCGTGGTTTCCGGTTCCGATACCCATGTGAAGGGCCTTCCCA
Bar1a 5 CC	AAAAARCATGGAAAGAATGATTAATTGAATTAATTGTAGCGTGGTTTCCGGTTCCGATACCCATGTGAAGGGCCTTCCCA
Bar1a 6 CC	AAAAAGCATGGAAAGAATGATTAATTGAATTAATTGTAGCGTGGTTTCCGGTTCCGATACCCATGTGAAGGGCCTTCCCA
Bar1a 8 CC	AAAAAACATGGAAAGAATGATTAATTGAATTAATTGTAGCGTGGTTTCCGGTTCCGATACCCATGTGAAGGGCCTTCCCA
Bar1a 9 CC	AAAAARCATGGAAAGAATGATTAATTGAATTAATTGTAGCGTGGTTTCCGGTTCCGATACCCATGTGAAGGGCCTTCCCA
Bar1a 10 CC	AAAAAGCATGGAAAGAATGATTAATTGAATTAATTGTAGCGTGGTTTCCGGTTCCGATACCCATGTGAAGGGCCTTCCCA
Bar1a 11 CC	AAAAARCATGGAAAGAATGATTAATTGAATTAATTGTAGCGTGGTTTCCGGTTCCGATACCCATGTGAAGGGCCTTCCCA
Bar1a 13 CC	AAAAARCATGGAAAGAATGATTAATTGAATTAATTGTAGCGTGGTTTCCGGTTCCGATACCCATGTGAAGGGCCTTCCCA
Bar1a 14 CC	AAAAAGCATGGAAAGAATGATTAATTGAATTAATTGTAGCGTGGTTTCCGGTTCCGATACCCATGTGAAGGGCCTTCCCA
Bar1a 15 CC	AAAAARCATGGAAAGAATGATTAATTGAATTAATTGTAGCGTGGTTTCCGGTTCCGATACCCATGTGAAGGGCCTTCCCA
Bar1a 16 CC	AAAAARCATGGAAAGAATGATTAATTGAATTAATTGTAGCGTGGTTTCCGGTTCCGATACCCATGTGAAGGGCCTTCCCA
Bar1a 17 CC	AAAAAGCATGGAAAGAATGATTAATTGAATTAATTGTAGCGTGGTTTCCGGTTCCGATACCCATGTGAAGGGCCTTCCCA
Bar1a 18 CC	AAAAARCATGGAAAGAATGATTAATTGAATTAATTGTAGCGTGGTTTCCGGTTCCGATACCCATGTGAAGGGCCTTCCCA
Bar1a 19 CC	AAAAARCATGGAAAGAATGATTAATTGAATTAATTGTAGCGTGGTTTCCGGTTCCGATACCCATGTGAAGGGCCTTCCCA
Bar1c 1 CC	AAAAARCATGGAAAGAATGATTAATTGAATTAATTGTAGCGTGGTTTCCGGTTCCGATACCCATGTGAAGGGCCTTCCCA
Bar1c 2 CC	AAAAARCATGGAAAGAATGATTAATTGAATTAATTGTAGCGTGGTTTCCGGTTCCGATACCCATGTGAAGGGCCTTCCCA
Bar2 2 CC	AAAAAGCATGGAAAGAATGATTAATTGAATTAATTGTAGCGTGGTTTCCGGTTCCGATACCCATGTGAAGGGCCTTCCCA
Bar2a 4 CC	AAAAAGCATGGAAAGAATGATTAATTGAATTAATTGTAGCGTGGTTTCCGGTTCCGATACCCATGTGAAGGGCCTTCCCA
Bar2a 5 CC	AAAAARCATGGAAAGAATGATTAATTGAATTAATTGTAGCGTGGTTTCCGGTTCCGATACCCATGTGAAGGGCCTTCCCA
Bar2b 1 CC	AAAAAGCATGGAAAGAATGATTAATTGAATTAATTGTAGCGTGGTTTCCGGTTCCGATACCCATGTGAAGGGCCTTCCCA
Bar2b 2 CC	AAAAARCATGGAAAGAATGATTAATTGAATTAATTGTAGCGTGGTTTCCGGTTCCGATACCCATGTGAAGGGCCTTCCCA
Bar2c 6 CC	AAAAARCATGGAAAGAATGATTAATTGAATTAATTGTAGCGTGGTTTCCGGTTCCGATACCCATGTGAAGGGCCTTCCCA
SJ1 4 CC	AAAAAGCATGGAAAGAATGATTAATTGAATTAATTGTAGCGTGGTTTCCGGTTCCGATACCCATGTGAAGGGCCTTCCCA
SJ1 6 CC	AAAAAGCATGGAAAGAATGATTAATTGAATTAATTGTAGCGTGGTTTCCGGTTCCGATACCCATGTGAAGGGCCTTCCCA
SJ1 7 CC	AAAAAGCATGGAAAGAATGATTAATTGAATTAATTGTAGCGTGGTTTCCGGTTCCGATACCCATGTGAAGGGCCTTCCCA
SJ2 4 CC	AAAAAGCATGGAAAGAATGATTAATTGAATTAATTGTAGCGTGGTTTCCGGTTCCGATACCCATGTGAAGGGCCTTCCCA
SJ2 5 CC	AAAAAGCATGGAAAGAATGATTAATTGAATTAATTGTAGCGTGGTTTCCGGTTCCGATACCCATGTGAAGGGCCTTCCCA
SJ2 6 CC	AAAAAGCATGGAAAGAATGATTAATTGAATTAATTGTAGCGTGGTTTCCGGTTCCGATACCCATGTGAAGGGCCTTCCCA
SJ2 7 CC	AAAAAGCATGGAAAGAATGATTAATTGAATTAATTGTAGCGTGGTTTCCGGTTCCGATACCCATGTGAAGGGCCTTCCCA
SJ3 4 CC	AAAAAGCATGGAAAGAATGATTAATTGAATTAATTGTAGCGTGGTTTCCGGTTCCGATACCCATGTGAAGGGCCTTCCCA
SJ3 5 CC	AAAAAGCATGGAAAGAATGATTAATTGAATTAATTGTAGCGTGGTTTCCGGTTCCGATACCCATGTGAAGGGCCTTCCCA
SJ3 6 CC	AAAAAGCATGGAAAGAATGATTAATTGAATTAATTGTAGCGTGGTTTCCGGTTCCGATACCCATGTGAAGGGCCTTCCCA
SJ3 8 CC	AAAAAGCATGGAAAGAATGATTAATTGAATTAATTGTAGCGTGGTTTCCGGTTCCGATACCCATGTGAAGGGCCTTCCCA
SJ25 3 CC	AAAAAGCATGGAAAGAATGATTAATTGAATTAATTGTAGCGTGGTTTCCGGTTCCGATACCCATGTGAAGGGCCTTCCCA

	1 10 20 30 40 50 60 70 80
Coos 3 CC	AAAAAGCATGGAAAGAATGATTAATTGAATTAATTGTAGCGTGGTTTCCGGTTCCGATACCCATGTGAAGGGCCTTCCCA
CABC1 CC	AAAAAGCATGGAAAGAATGATTAATTGAATTAATTGTAGCGTGGTTTCCGGTTCCGATACCCATGTGAAGGGCCTTCCCA
CABC2 CS	AAAAAGCATGGAAAKAATGATTAATTGAATTAWTTKTAGCGTGGTTTCCGRTTCCGATACCCAWGTGAAGGGCCTTCCCA
CABC3 CC	AAAAAGCATGGAAAGAATGATTAATTGAATTAATTGTAGCGTGGTTTCCGGTTCCGATACCCATGTGAAGGGCCTTCCCA
CABC4 CC	AAAAAGCATGGAAAGAATGATTAATTGAATTAATTGTAGCGTGGTTTCCGGTTCCGATACCCATGTGAAGGGCCTTCCCA
CABC5 CS	AAAAAGCATGGAAAKAATGATTAATTGAATTAWTTKTAGCGTGGTTTCCGRTTCCGATACCCAWGTGAAGGGCCTTCCCA
CASM1 CS	AAAAAGCATGGAAAKAATGATTAATTGAATTAWTTKTAGCGTGGTTTCCGRTTCCGATACCCAWGTGAAGGGCCTTCCCA
CASM3 CS	AAAAAGCATGGAAAKAATGATTAATTGAATTAWTTKTAGCGTGGTTTCCGRTTCCGATACCCAWGTGAAGGGCCTTCCCA
CASM9 CC	AAAAAGCATGGAAAGAATGATTAATTGAATTAATTGTAGCGTGGTTTCCGGTTCCGATACCCATGTGAAGGGCCTTCCCA
CASM11 CC	AAAAAGCATGGAAAGAATGATTAATTGAATTAATTGTAGCGTGGTTTCCGGTTCCGATACCCATGTGAAGGGCCTTCCCA
CASM13 CS	AAAAAGCATGGAAAKAATGATTAATTGAATTAWTTKTAGCGTGGTTTCCGRTTCCGATACCCAWGTGAAGGGCCTTCCCA
CB5 CC	AAAAAGCATGGAAAGAATGATTAATTGAATTAATTGTAGCGTGGTTTCCGGTTCCGATACCCATGTGAAGGGCCTTCCCA
CC2 6 CC	AAAAAGCATGGAAAGAATGATTAATTGAATTAATTGTAGCGTGGTTTCCGGTTCCGATACCCATGTGAAGGGCCTTCCCA
CD 2 CC	AAAAAGCATGGAAAGAATGATTAATTGAATTAATTGTAGCGTGGTTTCCGGTTCCGATACCCATGTGAAGGGCCTTCCCA
UK Gil 1 CL CC	AAAAARCATGGAAAGAATGATTAATTGAATTAATTGTAGCGTGGTTTCCGGTTCCGATACCCATGTGAAGGGCCTTCCCA
UK Gil 3 CC	AAAAARCATGGAAAGAATGATTAATTGAATTAATTGTAGCGTGGTTTCCGGTTCCGATACCCATGTGAAGGGCCTTCCCA
UK Gil 4 CC	AAAAARCATGGAAAGAATGATTAATTGAATTAATTGTAGCGTGGTTTCCGGTTCCGATACCCATGTGAAGGGCCTTCCCA
UK Gil 5 CC	AAAAARCATGGAAAGAATGATTAATTGAATTAATTGTAGCGTGGTTTCCGGTTCCGATACCCATGTGAAGGGCCTTCCCA
UK Gil 6 CC	AAAAARCATGGAAAGAATGATTAATTGAATTAATTGTAGCGTGGTTTCCGGTTCCGATACCCATGTGAAGGGCCTTCCCA
UK Gil 7 CC	AAAAARCATGGAAAGAATGATTAATTGAATTAATTGTAGCGTGGTTTCCGGTTCCGATACCCATGTGAAGGGCCTTCCCA
UK Gil 8 CC	AAAAARCATGGAAAGAATGATTAATTGAATTAATTGTAGCGTGGTTTCCGGTTCCGATACCCATGTGAAGGGCCTTCCCA
Gil 10 CC	AAAAARCATGGAAAGAATGATTAATTGAATTAATTGTAGCGTGGTTTCCGGTTCCGATACCCATGTGAAGGGCCTTCCCA
Gil 11 CC	AAAAARCATGGAAAGAATGATTAATTGAATTAATTGTAGCGTGGTTTCCGGTTCCGATACCCATGTGAAGGGCCTTCCCA
UK NrSalt 1 CC	AAAAARCATGGAAAGAATGATTAATTGAATTAATTGTAGCGTGGTTTCCGGTTCCGATACCCATGTGAAGGGCCTTCCCA
UK NrSalt 2 CC	AAAAARCATGGAAAGAATGATTAATTGAATTAATTGTAGCGTGGTTTCCGGTTCCGATACCCATGTGAAGGGCCTTCCCA
UK NrSalt 3 CC	AAAAARCATGGAAAGAATGATTAATTGAATTAATTGTAGCGTGGTTTCCGGTTCCGATACCCATGTGAAGGGCCTTCCCA
UK NrSalt 4 CC	AAAAARCATGGAAAGAATGATTAATTGAATTAATTGTAGCGTGGTTTCCGGTTCCGATACCCATGTGAAGGGCCTTCCCA
UK Nr Salt 5 CC	AAAAARCATGGAAAGAATGATTAATTGAATTAATTGTAGCGTGGTTTCCGGTTCCGATACCCATGTGAAGGGCCTTCCCA
UK Nr Salt 6 CC	AAAAARCATGGAAAGAATGATTAATTGAATTAATTGTAGCGTGGTTTCCGGTTCCGATACCCATGTGAAGGGCCTTCCCA
UK Nr Salt 7 CC	AAAAARCATGGAAAGAATGATTAATTGAATTAATTGTAGCGTGGTTTCCGGTTCCGATACCCATGTGAAGGGCCTTCCCA
UK Nr Salt 8 CC	AAAAARCATGGAAAGAATGATTAATTGAATTAATTGTAGCGTGGTTTCCGGTTCCGATACCCATGTGAAGGGCCTTCCCA
UK Nr Salt 9 CC	AAAAARCATGGAAAGAATGATTAATTGAATTAATTGTAGCGTGGTTTCCGGTTCCGATACCCATGTGAAGGGCCTTCCCA
UK NrSalt 10 CC	AAAAARCATGGAAAGAATGATTAATTGAATTAATTGTAGCGTGGTTTCCGGTTCCGATACCCATGTGAAGGGCCTTCCCA
UK NrSalt 11 CC	AAAAARCATGGAAAGAATGATTAATTGAATTAATTGTAGCGTGGTTTCCGGTTCCGATACCCATGTGAAGGGCCTTCCCA
UK NrSalt 13 CC	AAAAARCATGGAAAGAATGATTAATTGAATTAATTGTAGCGTGGTTTCCGGTTCCGATACCCATGTGAAGGGCCTTCCCA
UK NrSalt 14 CC	AAAAARCATGGAAAGAATGATTAATTGAATTAATTGTAGCGTGGTTTCCGGTTCCGATACCCATGTGAAGGGCCTTCCCA
UK NrSalt 15 CC	AAAAARCATGGAAAGAATGATTAATTGAATTAATTGTAGCGTGGTTTCCGGTTCCGATACCCATGTGAAGGGCCTTCCCA
UK Salt 1 CC	AAAAARCATGGAAAGAATGATTAATTGAATTAATTGTAGCGTGGTTTCCGGTTCCGATACCCATGTGAAGGGCCTTCCCA
UK Salt 3 CC	AAAAARCATGGAAAGAATGATTAATTGAATTAATTGTAGCGTGGTTTCCGGTTCCGATACCCATGTGAAGGGCCTTCCCA
UK Salt 4 CC	AAAAARCATGGAAAGAATGATTAATTGAATTAATTGTAGCGTGGTTTCCGGTTCCGATACCCATGTGAAGGGCCTTCCCA
UK Salt 5 CC	AAAAARCATGGAAAGAATGATTAATTGAATTAATTGTAGCGTGGTTTCCGGTTCCGATACCCATGTGAAGGGCCTTCCCA
UK Salt 6 CC	AAAAARCATGGAAAGAATGATTAATTGAATTAATTGTAGCGTGGTTTCCGGTTCCGATACCCATGTGAAGGGCCTTCCCA
UK Salt 7 CC	AAAAARCATGGAAAGAATGATTAATTGAATTAATTGTAGCGTGGTTTCCGGTTCCGATACCCATGTGAAGGGCCTTCCCA
UK Salt 8 CC	AAAAARCATGGAAAGAATGATTAATTGAATTAATTGTAGCGTGGTTTCCGGTTCCGATACCCATGTGAAGGGCCTTCCCA
UK Salt 9 CC	AAAAARCATGGAAAGAATGATTAATTGAATTAATTGTAGCGTGGTTTCCGGTTCCGATACCCATGTGAAGGGCCTTCCCA
UK Salt 10 CC	AAAAARCATGGAAAGAATGATTAATTGAATTAATTGTAGCGTGGTTTCCGGTTCCGATACCCATGTGAAGGGCCTTCCCA

	81 90 100 110 120 130 140 150 159
NS D88 02 CS	TGGGGGACTCCCTGGGCAGTTTTYGACTTCTAAAAGCAAGTCATACCCGTCTGTCCAGGTAATTCTATTGTTTACCTGA
NS D88 03 CC	TGGGGGACTCCCTGGGCAGTTTTCGACTTYTAAAAGCAAGTCATACCCGTCTGTCCAGGTAATTCTATTGTTTACCTCA
NS D88 04 CS	TGGGGGACTCCCTGGGCAGTTTTCGACTTCTAAAAGCAAGTCATACCCGTCTGTCCAGGTAATTCTATTGTTTACCTSA
NS D296 03 SS	TGGGGGACTCCCTGGGCAGTTTTYGACTTCTAAAAGCAAGTCATACCCGTCTGTCCAGGTAATTCTATTGTTTACCTGA
NS D296 07 CS	TGGGGGACTCCCTGGGCAGTTTTCGACTTCTAAAAGCAAGTCATACCCGTCTGTCCAGGTAATTCTATTGTTTACCTSA
NS D296 08 CS	TGGGGGACTCCCTGGGCAGTTTTCGACTTCTAAAAGCAAGTCATACCCGTCTGTCCAGGTAATTCTATTGTTTACCTSA
NS D296 09 CS	TGGGGGACTCCCTGGGCAGTTTTYGACTTCTAAAAGCAAGTCATACCCGTCTGTCCAGGTAATTCTATTGTTTACCTGA
NS D296 11 CS	TGGGGGACTCCCTGGGCAGTTTTCGACTTCTAAAAGCAAGTCATACCCGTCTGTCCAGGTAATTCTATTGTTTACCTGA
NS D296 12 CS	TGGGGGACTCCCTGGGCAGTTTTCGACTTCTAAAAGCAAGTCATACCCGTCTGTCCAGGTAATTCTATTGTTTACCTSA
Hal1 4 CS	TGGGGGACTCCCTGGGCAGTTTTCGACTTCTAAAAGCAAGTCATACCCGTCTGTCCAGGTAATTCTATTGTTTACCTSA
Hal1 5 CS	TGGGGGACTCCCTGGGCAGTTTTCGACTTCTAAAAGCAAGTCATACCCGTCTGTCCAGGTAATTCTATTGTTTACCTSA
Hal1 6 CS	TGGGGGACTCCCTGGGCAGTTTTCGACTTCTAAAAGCAAGTCATACCCGTCTGTCCAGGTAATTCTATTGTTTACCTSA
Hal1 7 CS	TGGGGGACTCCCTGGGCAGTTTTCGACTTCTAAAAGCAAGTCATACCCGTCTGTCCAGGTAATTCTATTGTTTACCTSA
Hal1 8 CS	TGGGGGACTCCCTGGGCAGTTTTCGACTTCTAAAAGCAAGTCATACCCGTCTGTCCAGGTAATTCTATTGTTTACCTSA
Hal1 9 SS	TGGGGGACTCCCTGGGCAGTTTTCGACTTCTAAAAGCAAGTCATACCCGTCTGTCCAGGTAATTCTATTGTTTACCTGA
Hal1 10 CS	TGGGGGACTCCCTGGGCAGTTTTCGACTTCTAAAAGCAAGTCATACCCGTCTGTCCAGGTAATTCTATTGTTTACCTSA
Hal1 11 CS	TGGGGGACTCCCTGGGCAGTTTTCGACTTCTAAAAGCAAGTCATACCCGTCTGTCCAGGTAATTCTATTGTTTACCTSA
Hal1 12 CS	TGGGGGACTCCCTGGGCAGTTTTCGACTTCTAAAAGCAAGTCATACCCGTCTGTCCAGGTAATTCTATTGTTTACCTSA
Hal1 14 CS	TGGGGGACTCCCTGGGCAGTTTTCGACTTCTAAAAGCAAGTCATACCCGTCTGTCCAGGTAATTCTATTGTTTACCTSA
Hal1 15 CS	TGGGGGACTCCCTGGGCAGTTTTCGACTTCTAAAAGCAAGTCATACCCGTCTGTCCAGGTAATTCTATTGTTTACCTSA
Hal1 16 CS	TGGGGGACTCCCTGGGCAGTTTTCGACTTCTAAAAGCAAGTCATACCCGTCTGTCCAGGTAATTCTATTGTTTACCTSA
Hal1 17 CS	TGGGGGACTCCCTGGGCAGTTTTCGACTTCTAAAAGCAAGTCATACCCGTCTGTCCAGGTAATTCTATTGTTTACCTSA
Hal1 19 CS	TGGGGGACTCCCTGGGCAGTTTTCGACTTCTAAAAGCAAGTCATACCCGTCTGTCCAGGTAATTCTATTGTTTACCTSA
Hal1 20 CS	TGGGGGACTCCCTGGGCAGTTTTCGACTTCTAAAAGCAAGTCATACCCGTCTGTCCAGGTAATTCTATTGTTTACCTSA
Hal1 21 SS	TGGGGGACTCCCTGGGCAGTTTTCGACTTCTAAAAGCAAGTCATACCCGTCTGTCCAGGTAATTCTATTGTTTACCTGA
Hal1 23 CS	TGGGGGACTCCCTGGGCAGTTTTCGACTTCTAAAAGCAAGTCATACCCGTCTGTCCAGGTAATTCTATTGTTTACCTSA
NS F299 1 SS	TGGGGGACTCCCTGGGCAGTTTTCGACTTCTAAAAGCAAGTCATACCCGTCTGTCCAGGTAATTCTATTGTTTACCTGA
NS F299 3 SS	TGGGGGACTCCCTGGGCAGTTTTCGACTTCTAAAAGCAAGTCATACCCGTCTGTCCAGGTAATTCTATTGTTTACCTGA
NS F299 4 CC	TGGGGGACTCCCTGGGCAGTTTTCGACTTCTAAAAGCAAGTCATACCCGTCTGTCCAGGTAATTCTATTGTTTACCTCA
NS F299 5 SS	TGGGGGACTCCCTGGGCAGTTTTYGACTTCTAAAAGCAAGTCATACCCGTCTGTCCAGGTAATTCTATTGTTTACCTGA
NS F299 6 SS	TGGGGGACTCCCTGGGCAGTTTTCGACTTCTAAAAGCAAGTCATACCCGTCTGTCCAGGTAATTCTATTGTTTACCTGA
NS F 299 8 SS	TGGGGGACTCCCTGGGCAGTTTTYGACTTCTAAAAGCAAGTCATACCCGTCTGTCCAGGTAATTCTATTGTTTACCTGA
NS F299 9 SS	TGGGGGACTCCCTGGGCAGTTTTCGACTTCTAAAAGCAAGTCATACCCGTCTGTCCAGGTAATTCTATTGTTTACCTGA
NS F299 10 SS	TGGGGGACTCCCTGGGCAGTTTTYGACTTCTAAAAGCAAGTCATACCCGTCTGTCCAGGTAATTCTATTGTTTACCTGA
NS F299 11 SS	TGGGGGACTCCCTGGGCAGTTTTCGACTTCTAAAAGCAAGTCATACCCGTCTGTCCAGGTAATTCTATTGTTTACCTGA
NS B293 1 CC	TGGGGGACTCCCTGGGCAGTTTTCGACTTCTAAAAGCAAGTCATACCCGTCTGTCCAGGTAATTCTATTGTTTACCTCA
NS B293 2 CC	TGGGGGACTCCCTGGGCAGTTTTCGACTTCTAAAAGCAAGTCATACCCGTCTGTCCAGGTAATTCTATTGTTTACCTCA
NS B293 3 CC	TGGGGGACTCCCTGGGCAGTTTTCGACTTCTAAAAGCAAGTCATACCCGTCTGTCCAGGTAATTCTATTGTTTACCTCA
NS B293 4 CC	TGGGGGACTCCCTGGGCAGTTTTCGACTTCTAAAAGCAAGTCATACCCGTCTGTCCAGGTAATTCTATTGTTTACCTCA
NS B293 5 CC	TGGGGGACTCCCTGGGCAGTTTTCGACTTCTAAAAGCAAGTCATACCCGTCTGTCCAGGTAATTCTATTGTTTACCTCA
NS B293 6 Cy	TGGGGGACTCCCTGGGCAGTTTTCGACTTCTAAAAGCAAGTCATACCCGTCTGTCCAGGTAATTCTATTGTTTACCTCA
NS B293 7 CC	TGGGGGACTCCCTGGGCAGTTTTCGACTTCTAAAAGCAAGTCATACCCGTCTGTCCAGGTAATTCTATTGTTTACCTCA
NS B293 8 CC	TGGGGGACTCCCTGGGCAGTTTTCGACTTCTAAAAGCAAGTCATACCCGTCTGTCCAGGTAATTCTATTGTTTACCTCA
NS B293 9 CC	TGGGGGACTCCCTGGGCAGTTTTCGACTTCTAAAAGCAAGTCATACCCGTCTGTCCAGGTAATTCTATTGTTTACCTCA
NS B293 10 CC	TGGGGGACTCCCTGGGCAGTTTTCGACTTCTAAAAGCAAGTCATACCCGTCTGTCCAGGTAATTCTATTGTTTACCTCA
NS B293 11 CC	TGGGGGACTCCCTGGGCAGTTTTYGACTTCTAAAAGCAAGTCATACCCGTCTGTCCAGGTAATTCTATTGTTTACCTCA
NS B294 1 CC	TGGGGGACTCCCTGGGCAGTTTTCGACTTCTAAAAGCAAGTCATACCCGTCTGTCCAGGTAATTCTATTGTTTACCTCA
NS B294 2 CC	TGGGGGACTCCCTGGGCAGTTTTCGACTTCTAAAAGCAAGTCATACCCGTCTGTCCAGGTAATTCTATTGTTTACCTCA
NS B294 4 CC	TGGGGGACTCCCTGGGCAGTTTTYGACTTCTAAAAGCAAGTCATACCCGTCTGTCCAGGTAATTCTATTGTTTACMTSA
NS B294 5 CC	TGGGGGACTCCCTGGGCAGTTTTCGACTTCTAAAAGCAAGTCATACCCGTCTGTCCAGGTAATTCTATTGTTTACCTCA
NS B294 6 CC	TGGGGGACTCCCTGGGCAGTTTTCGACTTCTAAAAGCAAGTCATACCCGTCTGTCCAGGTAATTCTATTGTTTACCTCA

	81 90 100 110 120 130 140 150 159
NS B294 7 CC	TGGGGGACTCCCTGGGCAGTTTTYGACTTCTAAAAGCAAGTCATACCCGTCTGTCCAGGTAATTCTATTGTTTACCTCA
NS B294 9 CC	TGGGGGACTCCCTGGGCAGTTTTCGACTTCTAAAAGCAAGTCATACCCGTCTGTCCAGGTAATTCTATTGTTTACCTCA
NS B294 10 CC	TGGGGGACTCCCTGGGCAGTTTTCGACTTCTAAAAGCAAGTCATACCCGTCTGTCCAGGTAATTCTATTGTTTACCTCA
NS A291 3 CS	TGGGGGACTCCCTGGGCAGTTTTYGACTTCTAAAAGCAAGTCATACCCGTCTGTCCAGGTAATTCTATTGTTTACCTGA
NS A291 4 CC	TGGGGGACTCCCTGGGCAGTTTTCGACTTCTAAAAGCAAGTCATACCCGTCTGTCCAGGTAATTCTATTGTTTACCTCA
NS A291 5 CS	TGGGGGACTCCCTGGGCAGTTTTCGACTTCTAAAAGCAAGTCATACCCGTCTGTCCAGGTAATTCTATTGTTTACCTSA
NS A291 6 CS	TGGGGGACTCCCTGGGCAGTTTTCGACTTCTAAAAGCAAGTCATACCCGTCTGTCCAGGTAATTCTATTGTTTACCTSA
NS A291 7 CS	TGGGGGACTCCCTGGGCAGTTTTCGACTTCTAAAAGCAAGTCATACCCGTCTGTCCAGGTAATTCTATTGTTTACCTSA
NS A291 8 CS	TGGGGGACTCCCTGGGCAGTTTTCGACTTCTAAAAGCAAGTCATACCCGTCTGTCCAGGTAATTCTATTGTTTACCTSA
NS A291 10 CS	TGGGGGACTCCCTGGGCAGTTTTCGACTTCTAAAAGCAAGTCATACCCGTCTGTCCAGGTAATTCTATTGTTTACCTSA
NS A291 11 CS	TGGGGGACTCCCTGGGCAGTTTTCGACTTCTAAAAGCAAGTCATACCCGTCTGTCCAGGTAATTCTATTGTTTACCTSA
NS A291 12 CS	TGGGGGACTCCCTGGGCAGTTTTCGACTTCTAAAAGCAAGTCATACCCGTCTGTCCAGGTAATTCTATTGTTTACCTSA
NS A291 13 CS	TGGGGGACTCCCTGGGCAGTTTTYGACTTCTAAAAGCAAGTCATACCCGTCTGTCCAGGTAATTCTATTGTTTACCTSA
NS A291 15 CS	TGGGGGACTCCCTGGGCAGTTTTCGACTTCTAAAAGCAAGTCATACCCGTCTGTCCAGGTAATTCTATTGTTTACCTSA
NS A291 17 CS	TGGGGGACTCCCTGGGCAGTTTTCGACTTCTAAAAGCAAGTCATACCCGTCTGTCCAGGTAATTCTATTGTTTACCTSA
NS A291 18 CS	TGGGGGACTCCCTGGGCAGTTTTYGACTTCTAAAAGCAAGTCATACCCGTCTGTCCAGGTAATTCTATTGTTTACCTSA
NS A291 19 CC	TGGGGGACTCCCTGGGCAGTTTTYGACTTCTAAAAGCAAGTCATACCCGTCTGTCCAGGTAATTCTATTGTTTACCTCA
NS A291 20 CS	TGGGGGACTCCCTGGGCAGTTTTCGACTTCTAAAAGCAAGTCATACCCGTCTGTCCAGGTAATTCTATTGTTTACCTSA
NS A291 21 CS	TGGGGGACTCCCTGGGCAGTTTTCGACTTCTAAAAGCAAGTCATACCCGTCTGTCCAGGTAATTCTATTGTTTACCTSA
NS E298 1 CC	TGGGGGACTCCCTGGGCAGTTTTCGACTTCTAAAAGCAAGTCATACCCGTCTGTCCAGGTAATTCTATTGTTTACCTCA
NS E298 2 CC	TGGGGGACTCCCTGGGCAGTTTTCGACTTCTAAAAGCAAGTCATACCCGTCTGTCCAGGTAATTCTATTGTTTACCTCA
NS E298 3 CC	TGGGGGACTCCCTGGGCAGTTTTYGACTTCTAAAAGCAAGTCATACCCGTCTGTCCAGGTAATTCTATTGTTTACCTCA
NS E298 4 SS	TGGGGGACTCCCTGGGCAGTTTTCGACTTCTAAAAGCAAGTCATACCCGTCTGTCCAGGTAATTCTATTGTTTACCTGA
NS E298 5 CC	TGGGGGACTCCCTGGGCAGTTTTCGACTTCTAAAAGCAAGTCATACCCGTCTGTCCAGGTAATTCTATTGTTTACCTCA
NS E298 6 CC	TGGGGGACTCCCTGGGCAGTTTTCGACTTCTAAAAGCAAGTCATACCCGTCTGTCCAGGTAATTCTATTGTTTACCTCA
NS E298 7 CC	TGGGGGACTCCCTGGGCAGTTTTCGACTTCTAAAAGCAAGTCATACCCGTCTGTCCAGGTAATTCTATTGTTTACCTCA
NS E298 8 CC	TGGGGGACTCCCTGGGCAGTTTTCGACTTCTAAAAGCAAGTCATACCCGTCTGTCCAGGTAATTCTATTGTTTACCTCA
NS E298 9 CC	TGGGGGACTCCCTGGGCAGTTTTYGACTTCTAAAAGCAAGTCATACCCGTCTGTCCAGGTAATTCTATTGTTTACCTCA
NS E298 10 CC	TGGGGGACTCCCTGGGCAGTTTTYGACTTCTAAAAGCAAGTCATACCCGTCTGTCCAGGTAATTCTATTGTTTACCTCA
KNG 1 1 CC	TGGGGGACTCCCTGGGCAGTTTTCGACTTCTAAAAGCAAGTCATACCCGTCTGTCCAGGTAATTCTATTGTTTACCTCA
KNG 1 2 CC	TGGGGGACTCCCTGGGCAGTTTTCGACTTCTAAAAGCAAGTCATACCCGTCTGTCCAGGTAATTCTATTGTTTACCTCA
KNG 1 3 CC	TGGGGGACTCCCTGGGCAGTTTTCGACTTCTAAAAGCAAGTCATACCCGTCTGTCCAGGTAATTCTATTGTTTACCTCA
KNG 1 4 CC	TGGGGGACTCCCTGGGCAGTTTTCGACTTCTAAAAGCAAGTCATACCCGTCTGTCCAGGTAATTCTATTGTTTACCTCA
KNG 1 5 CC	TGGGGGACTCCCTGGGCAGTTTTCGACTTCTAAAAGCAAGTCATACCCGTCTGTCCAGGTAATTCTATTGTTTACCTCA
KNG 1 6 CC	TGGGGGACTCCCTGGGCAGTTTTCGACTTCTAAAAGCAAGTCATACCCGTCTGTCCAGGTAATTCTATTGTTTACCTCA
KNG 1 7 CC	TGGGGGACTCCCTGGGCAGTTTTCGACTTCTAAAAGCAAGTCATACCCGTCTGTCCAGGTAATTCTATTGTTTACCTCA
KNG 1 8 CC	TGGGGGACTCCCTGGGCAGTTTTCGACTTCTAAAAGCAAGTCATACCCGTCTGTCCAGGTAATTCTATTGTTTACCTCA
KNG 1 9 CC	TGGGGGACTCCCTGGGCAGTTTTCGACTTCTAAAAGCAAGTCATACCCGTCTGTCCAGGTAATTCTATTGTTTACCTCA
KNG 1 11 CC	TGGGGGACTCCCTGGGCAGTTTTCGACTTCTAAAAGCAAGTCATACCCGTCTGTCCAGGTAATTCTATTGTTTACCTCA
KNG 1 12 CC	TGGGGGACTCCCTGGGCAGTTTTCGACTTCTAAAAGCAAGTCATACCCGTCTGTCCAGGTAATTCTATTGTTTACCTCA
KNG 1 13 CC	TGGGGGACTCCCTGGGCAGTTTTCGACTTCTAAAAGCAAGTCATACCCGTCTGTCCAGGTAATTCTATTGTTTACCTCA
KNG 2 1 CC	TGGGGGACTCCCTGGGCAGTTTTCGACTTCTAAAAGCAAGTCATACCCGTCTGTCCAGGTAATTCTATTGTTTACCTCA
KNG 2 2 CC	TGGGGGACTCCCTGGGCAGTTTTCGACTTCTAAAAGCAAGTCATACCCGTCTGTCCAGGTAATTCTATTGTTTACCTCA
KNG 2 5 CC	TGGGGGACTCCCTGGGCAGTTTTCGACTTCTAAAAGCAAGTCATACCCGTCTGTCCAGGTAATTCTATTGTTTACCTCA
KNG 2 6 CC	TGGGGGACTCCCTGGGCAGTTTTCGACTTCTAAAAGCAAGTCATACCCGTCTGTCCAGGTAATTCTATTGTTTACCTCA
KNG 2 7 CC	TGGGGGACTCCCTGGGCAGTTTTCGACTTCTAAAAGCAAGTCATACCCGTCTGTCCAGGTAATTCTATTGTTTACCTCA
KNG 2 8 CC	TGGGGGACTCCCTGGGCAGTTTTCGACTTCTAAAAGCAAGTCATACCCGTCTGTCCAGGTAATTCTATTGTTTACCTCA
KNG 2 9 CC	TGGGGGACTCCCTGGGCAGTTTTCGACTTCTAAAAGCAAGTCATACCCGTCTGTCCAGGTAATTCTATTGTTTACCTCA
KNG 3 2 CC	TGGGGGACTCCCTGGGCAGTTTTCGACTTCTAAAAGCAAGTCATACCCGTCTGTCCAGGTAATTCTATTGTTTACCTCA
KNG 3 3 CC	TGGGGGACTCCCTGGGCAGTTTTCGACTTCTAAAAGCAAGTCATACCCGTCTGTCCAGGTAATTCTATTGTTTACCTCA
KNG 3 4 CC	TGGGGGACTCCCTGGGCAGTTTTCGACTTCTAAAAGCAAGTCATACCCGTCTGTCCAGGTAATTCTATTGTTTACCTCA

```
                 81        90                  100                 110                 120                 130                 140                 150              159
KNG 3 5 CC       TGGGGGACTCCCTGGGCAGTTTTCGACTTCTAAAAGCAAGTCATACCCGTCTGTCCAGGTAATTCTATTGTTTACCTCA
KNG 3 6 CC       TGGGGGACTCCCTGGGCAGTTTTCGACTTCTAAAAGCAAGTCATACCCGTCTGTCCAGGTAATTCTATTGTTTACCTCA
KNG 3 7 CC       TGGGGGACTCCCTGGGCAGTTTTCGACTTCTAAAAGCAAGTCATACCCGTCTGTCCAGGTAATTCTATTGTTTACCTCA
KNG 3 8 CC       TGGGGGACTCCCTGGGCAGTTTTCGACTTCTAAAAGCAAGTCATACCCGTCTGTCCAGGTAATTCTATTGTTTACCTCA
KNG 3 9 CC       TGGGGGACTCCCTGGGCAGTTTTCGACTTCTAAAAGCAAGTCATACCCGTCTGTCCAGGTAATTCTATTGTTTACCTCA
KNG 3 10 CC      TGGGGGACTCCCTGGGCAGTTTTCGACTTCTAAAAGCAAGTCATACCCGTCTGTCCAGGTAATTCTATTGTTTACCTCA
KNG 3 14-CC      TGGGGGACTCCCTGGGCAGTTTTYGACTTCTAAAAGCAAGTCATACCCGTCTGTCCAGGTAATTCTATTGTTTACCTSA
NS C1 CC         TGGGGGACTCCCTGGGCAGTTTTYGACTTCTAAAAGCAAGTCATACCCGTCTGTCCAGGTAATTCTATTGTTTACCTCA
NS C2 CC         TGGGGGACTCCCTGGGCAGTTTTCGACTTCTAAAAGCAAGTCATACCCGTCTGTCCAGGTAATTCTATTGTTTACCTCA
NS C3 CC         TGGGGGACTCCCTGGGCAGTTTTCGACTTCTAAAAGCAAGTCATACCCGTCTGTCCAGGTAATTCTATTGTTTACCTCA
NS C4 CC         TGGGGGACTCCCTGGGCAGTTTTCGACTTCTAAAAGCAAGTCATACCCGTCTGTCCAGGTAATTCTATTGTTTACCTCA
NS C6 CC         TGGGGGACTCCCTGGGCAGTTTTCGACTTCTAAAAGCAAGTCATACCCGTCTGTCCAGGTAATTCTATTGTTTACCTCA
NS C9 CC         TGGGGGACTCCCTGGGCAGTTTTCGACTTCTAAAAGCAAGTCATACCCGTCTGTCCAGGTAATTCTATTGTTTACCTCA
OD 1 CC          TGGGGGACTCCCTGGGCAGTTTTCGACTTCTAAAAGCAAGTCATACCCGTCTGTCCAGGTAATTCTATTGTTTACCTCA
OD 2 CC          TGGGGGACTCCCTGGGCAGTTTTCGACTTCTAAAAGCAAGTCATACCCGTCTGTCCAGGTAATTCTATTGTTTACCTCA
OD 3 CC          TGGGGGACTCCCTGGGCAGTTTTCGACTTCTAAAAGCAAGTCATACCCGTCTGTCCAGGTAATTCTATTGTTTACCTCA
OD 4 CC          TGGGGGACTCCCTGGGCAGTTTTCGACTTCTAAAAGCAAGTCATACCCGTCTGTCCAGGTAATTCTATTGTTTACCTCA
OD 5 CC          TGGGGGACTCCCTGGGCAGTTTTCGACTTCTAAAAGCAAGTCATACCCGTCTGTCCAGGTAATTCTATTGTTTACCTCA
OD 9 CC          TGGGGGACTCCCTGGGCAGTTTTCGACTTCTAAAAGCAAGTCATACCCGTCTGTCCAGGTAATTCTATTGTTTACCTCA
OD2004 3 CC      TGGGGGACTCCCTGGGCAGTTTTCGACTTCTAAAAGCAAGTCATACCCGTCTGTCCAGGTAATTCTATTGTTTACCTCA
OD2004 4 CC      TGGGGGACTCCCTGGGCAGTTTTCGACTTCTAAAAGCAAGTCATACCCGTCTGTCCAGGTAATTCTATTGTTTACCTCA
OD2004 6 CC      TGGGGGACTCCCTGGGCAGTTTTCGACTTCTAAAAGCAAGTCATACCCGTCTGTCCAGGTAATTCTATTGTTTACCTCA
OD2004 7 CC      TGGGGGACTCCCTGGGCAGTTTTCGACTTCTAAAAGCAAGTCATACCCGTCTGTCCAGGTAATTCTATTGTTTACCTCA
OD2004 10 CC     TGGGGGACTCCCTGGGCAGTTTTCGACTTCTAAAAGCAAGTCATACCCGTCTGTCCAGGTAATTCTATTGTTTACCTCA
OD2004 14 CC     TGGGGGACTCCCTGGGCAGTTTTCGACTTCTAAAAGCAAGTCATACCCGTCTGTCCAGGTAATTCTATTGTTTACCTCA
RH 4 CC          TGGGGGACTCCCTGGGCAGTTTTCGACTTCTAAAAGCAAGTCATACCCGTCTGTCCAGGTAATTCTATTGTTTACCTCA
RH 5 CC          TGGGGGACTCCCTGGGCAGTTTTCGACTTCTAAAAGCAAGTCATACCCGTCTGTCCAGGTAATTCTATTGTTTACCTCA
RH 6 CC          TGGGGGACTCCCTGGGCAGTTTTCGACTTCTAAAAGCAAGTCATACCCGTCTGTCCAGGTAATTCTATTGTTTACCTCA
RH 7 CC          TGGGGGACTCCCTGGGCAGTTTTCGACTTCTAAAAGCAAGTCATACCCGTCTGTCCAGGTAATTCTATTGTTTACCTCA
RH 9 CC          TGGGGGACTCCCTGGGCAGTTTTCGACTTCTAAAAGCAAGTCATACCCGTCTGTCCAGGTAATTCTATTGTTTACCTCA
RH 10 CC         TGGGGGACTCCCTGGGCAGTTTTCGACTTCTAAAAGCAAGTCATACCCGTCTGTCCAGGTAATTCTATTGTTTACCTCA
WSA1 1 2 CC      TGGGGGACTCCCTGGGCAGTTTTCGACTTCTAAAAGCAAGTCATACCCGTCTGTCCAGGTAATTCTATTGTTTACCTCA
WSA1 1 4 CC      TGGGGGACTCCCTGGGCAGTTTTCGACTTCTAAAAGCAAGTCATACCCGTCTGTCCAGGTAATTCTATTGTTTACCTCA
WSA1 1 5 CC      TGGGGGACTCCCTGGGCAGTTTTCGACTTCTAAAAGCAAGTCATACCCGTCTGTCCAGGTAATTCTATTGTTTACCTCA
WSA1 1 6 CC      TGGGGGACTCCCTGGGCAGTTTTCGACTTCTAAAAGCAAGTCATACCCGTCTGTCCAGGTAATTCTATTGTTTACCTCA
WSA1 1 9 CC      TGGGGGACTCCCTGGGCAGTTTTCGACTTCTAAAAGCAAGTCATACCCGTCTGTCCAGGTAATTCTATTGTTTACCTCA
WSA1 1 10 CC     TGGGGGACTCCCTGGGCAGTTTTYGACTTCTAAAAGCAAGTCATACCCGTCTGTCCAGGTAATTCTATTGTTTACCTCA
WSA1 1 11 CC     TGGGGGACTCCCTGGGCAGTTTTCGACTTCTAAAAGCAAGTCATACCCGTCTGTCCAGGTAATTCTATTGTTTACCTCA
WSA1 1 12 CC     TGGGGGACTCCCTGGGCAGTTTTCGACTTCTAAAAGCAAGTCATACCCGTCTGTCCAGGTAATTCTATTGTTTACCTCA
WSA1 1 16 CC     TGGGGGACTCCCTGGGCAGTTTTCGACTTCTAAAAGCAAGTCATACCCGTCTGTCCAGGTAATTCTATTGTTTACCTCA
WSA1 1 17 CC     TGGGGGACTCCCTGGGCAGTTTTCGACTTCTAAAAGCAAGTCATACCCGTCTGTCCAGGTAATTCTATTGTTTACCTCA
WSA1 1 18 CC     TGGGGGACTCCCTGGGCAGTTTTCGACTTCTAAAAGCAAGTCATACCCGTCTGTCCAGGTAATTCTATTGTTTACCTCA
WSA1 1 19 CC     TGGGGGACTCCCTGGGCAGTTTTCGACTTCTAAAAGCAAGTCATACCCGTCTGTCCAGGTAATTCTATTGTTTACCTCA
WSA1 1 20 CC     TGGGGGACTCCCTGGGCAGTTTTCGACTTCTAAAAGCAAGTCATACCCGTCTGTCCAGGTAATTCTATTGTTTACCTCA
WSA1 2 2 CC      TGGGGGACTCCCTGGGCAGTTTTCGACTTCTAAAAGCAAGTCATACCCGTCTGTCCAGGTAATTCTATTGTTTACCTCA
WSA1 2 3 CC      TGGGGGACTCCCTGGGCAGTTTTCGACTTCTAAAAGCAAGTCATACCCGTCTGTCCAGGTAATTCTATTGTTTACCTCA
WSA1 2 4 CC      TGGGGGACTCCCTGGGCAGTTTTCGACTTCTAAAAGCAAGTCATACCCGTCTGTCCAGGTAATTCTATTGTTTACCTCA
WSA1 2 5 CC      TGGGGGACTCCCTGGGCAGTTTTCGACTTCTAAAAGCAAGTCATACCCGTCTGTCCAGGTAATTCTATTGTTTACCTCA
WSA1 2 7 CC      TGGGGGACTCCCTGGGCAGTTTTCGACTTCTAAAAGCAAGTCATACCCGTCTGTCCAGGTAATTCTATTGTTTACCTCA
WSA2 1 CC        TGGGGGACTCCCTGGGCAGTTTTCGACTTCTAAAAGCAAGTCATACCCGTCTGTCCAGGTAATTCTATTGTTTACCTCA
WSA2 2 CC        TGGGGGACTCCCTGGGCAGTTTTCGACTTCTAAAAGCAAGTCATACCCGTCTGTCCAGGTAATTCTATTGTTTACCTCA
```

	81 90 100 110 120 130 140 150 159
WSA2 3 CC	TGGGGGACTCCCTGGGCAGTTTTCGACTTCTAAAAGCAAGTCATACCCGTCTGTCCAGGTAATTCTATTGTTTACCTCA
Cr 4 CC	TGGGGGACTCCCTGGGCAGTTTTCGACTTCTAAAAGCAAGTCATACCCGTCTGTCCAGGTAATTCTATTGTTTACCTCA
Cr CL2004 CC	TGGGGGACTCCCTGGGCAGTTTTCGACTTCTAAAAGCAAGTCATACCCGTCTGTCCAGGTAATTCTATTGTTTACCTCA
Cr2004 1 CC	TGGGGGACTCCCTGGGCAGTTTTCGACTTCTAAAAGCAAGTCATACCCGTCTGTCCAGGTAATTCTATTGTTTACCTCA
Cr2004 2 CC	TGGGGGACTCCCTGGGCAGTTTTCGACTTCTAAAAGCAAGTCATACCCGTCTGTCCAGGTAATTCTATTGTTTACCTCA
Cr2004 3 CC	TGGGGGACTCCCTGGGCAGTTTTCGACTTCTAAAAGCAAGTCATACCCGTCTGTCCAGGTAATTCTATTGTTTACCTCA
Cr2004 4 CC	TGGGGGACTCCCTGGGCAGTTTTCGACTTCTAAAAGCAAGTCATACCCGTCTGTCCAGGTAATTCTATTGTTTACCTCA
Cr2004 8 CC	TGGGGGACTCCCTGGGCAGTTTTCGACTTCTAAAAGCAAGTCATACCCGTCTGTCCAGGTAATTCTATTGTTTACCTCA
Cr2004 10 CC	TGGGGGACTCCCTGGGCAGTTTTCGACTTCTAAAAGCAAGTCATACCCGTCTGTCCAGGTAATTCTATTGTTTACCTCA
Cr2004 11 CC	TGGGGGACTCCCTGGGCAGTTTTCGACTTCTAAAAGCAAGTCATACCCGTCTGTCCAGGTAATTCTATTGTTTACCTCA
Cr2004 12 CC	TGGGGGACTCCCTGGGCAGTTTTCGACTTCTAAAAGCAAGTCATACCCGTCTGTCCAGGTAATTCTATTGTTTACCTCA
Cr2004 13 CC	TGGGGGACTCCCTGGGCAGTTTTCGACTTCTAAAAGCAAGTCATACCCGTCTGTCCAGGTAATTCTATTGTTTACCTCA
Cr2004 14 CC	TGGGGGACTCCCTGGGCAGTTTTCGACTTCTAAAAGCAAGTCATACCCGTCTGTCCAGGTAATTCTATTGTTTACCTCA
Nep2004 3 CC	TGGGGGACTCCCTGGGCAGTTTTCGACTTCTAAAAGCAAGTCATACCCGTCTGTCCAGGTAATTCTATTGTTTACCTCA
Nep2004 5 CC	TGGGGGACTCCCTGGGCAGTTTTCGACTTCTAAAAGCAAGTCATACCCGTCTGTCCAGGTAATTCTATTGTTTACCTCA
Nep2004 6 CC	TGGGGGACTCCCTGGGCAGTTTTCGACTTCTAAAAGCAAGTCATACCCGTCTGTCCAGGTAATTCTATTGTTTACCTCA
Nep2004 8 CC	TGGGGGACTCCCTGGGCAGTTTTCGACTTCTAAAAGCAAGTCATACCCGTCTGTCCAGGTAATTCTATTGTTTACCTCA
Nep2004 9 CC	TGGGGGACTCCCTGGGCAGTTTTCGACTTCTAAAAGCAAGTCATACCCGTCTGTCCAGGTAATTCTATTGTTTACCTCA
Nep2004 10 CC	TGGGGGACTCCCTGGGCAGTTTTCGACTTCTAAAAGCAAGTCATACCCGTCTGTCCAGGTAATTCTATTGTTTACCTCA
Nep2004 11 CC	TGGGGGACTCCCTGGGCAGTTTTCGACTTCTAAAAGCAAGTCATACCCGTCTGTCCAGGTAATTCTATTGTTTACCTCA
Nep2004 12 CC	TGGGGGACTCCCTGGGCAGTTTTCGACTTCTAAAAGCAAGTCATACCCGTCTGTCCAGGTAATTCTATTGTTTACCTCA
Nep2004 14 CC	TGGGGGACTCCCTGGGCAGTTTTCGACTTCTAAAAGCAAGTCATACCCGTCTGTCCAGGTAATTCTATTGTTTACCTCA
Nep2004 16 CC	TGGGGGACTCCCTGGGCAGTTTTCGACTTCTAAAAGCAAGTCATACCCGTCTGTCCAGGTAATTCTATTGTTTACCTCA
Nep2004 17 CC	TGGGGGACTCCCTGGGCAGTTTTCGACTTCTAAAAGCAAGTCATACCCGTCTGTCCAGGTAATTCTATTGTTTACCTCA
Nep2004 18 CC	TGGGGGACTCCCTGGGCAGTTTTCGACTTCTAAAAGCAAGTCATACCCGTCTGTCCAGGTAATTCTATTGTTTACCTCA
Nep2004 19 CC	TGGGGGACTCCCTGGGCAGTTTTCGACTTCTAAAAGCAAGTCATACCCGTCTGTCCAGGTAATTCTATTGTTTACCTCA
Nep2004 20 CC	TGGGGGACTCCCTGGGCAGTTTTCGACTTCTAAAAGCAAGTCATACCCGTCTGTCCAGGTAATTCTATTGTTTACCTCA
Nep2004 21 CC	TGGGGGACTCCCTGGGCAGTTTTCGACTTCTAAAAGCAAGTCATACCCGTCTGTCCAGGTAATTCTATTGTTTACCTCA
Nep2004 22 CC	TGGGGGACTCCCTGGGCAGTTTTCGACTTCTAAAAGCAAGTCATACCCGTCTGTCCAGGTAATTCTATTGTTTACCTCA
Nep2004 23 CC	TGGGGGACTCCCTGGGCAGTTTTCGACTTCTAAAAGCAAGTCATACCCGTCTGTCCAGGTAATTCTATTGTTTACCTCA
Nep2004 24 CC	TGGGGGACTCCCTGGGCAGTTTTCGACTTCTAAAAGCAAGTCATACCCGTCTGTCCAGGTAATTCTATTGTTTACCTCA
Nep2004 25 CC	TGGGGGACTCCCTGGGCAGTTTTCGACTTCTAAAAGCAAGTCATACCCGTCTGTCCAGGTAATTCTATTGTTTACCTCA
Nep2004 26 CC	TGGGGGACTCCCTGGGCAGTTTTCGACTTCTAAAAGCAAGTCATACCCGTCTGTCCAGGTAATTCTATTGTTTACCTCA
Nep2004 27 CC	TGGGGGACTCCCTGGGCAGTTTTCGACTTCTAAAAGCAAGTCATACCCGTCTGTCCAGGTAATTCTATTGTTTACCTCA
Nep2004 29 CC	TGGGGGACTCCCTGGGCAGTTTTCGACTTCTAAAAGCAAGTCATACCCGTCTGTCCAGGTAATTCTATTGTTTACCTCA
Nep2004 30 CC	TGGGGGACTCCCTGGGCAGTTTTCGACTTCTAAAAGCAAGTCATACCCGTCTGTCCAGGTAATTCTATTGTTTACCTCA
Poc 3 CC	TGGGGGACTCCCTGGGCAGTTTTCGACTTCTAAAAGCAAGTCATACCCGTCTGTCCAGGTAATTCTATTGTTTACCTCA
Poc 5 CC	TGGGGGACTCCCTGGGCAGTTTTCGACTTCTAAAAGCAAGTCATACCCGTCTGTCCAGGTAATTCTATTGTTTACCTCA
Poc 6 CC	TGGGGGACTCCCTGGGCAGTTTTCGACTTCTAAAAGCAAGTCATACCCGTCTGTCCAGGTAATTCTATTGTTTACCTCA
Poc 7 CC	TGGGGGACTCCCTGGGCAGTTTTCGACTTCTAAAAGCAAGTCATACCCGTCTGTCCAGGTAATTCTATTGTTTACCTCA
Poc 8 CC	TGGGGGACTCCCTGGGCAGTTTTCGACTTCTAAAAGCAAGTCATACCCGTCTGTCCAGGTAATTCTATTGTTTACCTCA
Poc 10 CC	TGGGGGACTCCCTGGGCAGTTTTCGACTTCTAAAAGCAAGTCATACCCGTCTGTCCAGGTAATTCTATTGTTTACCTCA
Poc 11 CC	TGGGGGACTCCCTGGGCAGTTTTCGACTTCTAAAAGCAAGTCATACCCGTCTGTCCAGGTAATTCTATTGTTTACCTCA
Poc 12 CC	TGGGGGACTCCCTGGGCAGTTTTCGACTTCTAAAAGCAAGTCATACCCGTCTGTCCAGGTAATTCTATTGTTTACCTCA
Poc 13 CC	TGGGGGACTCCCTGGGCAGTTTTCGACTTCTAAAAGCAAGTCATACCCGTCTGTCCAGGTAATTCTATTGTTTACCTCA
Poc 14 CC	TGGGGGACTCCCTGGGCAGTTTTCGACTTCTAAAAGCAAGTCATACCCGTCTGTCCAGGTAATTCTATTGTTTACCTCA
Poc 15 CC	TGGGGGACTCCCTGGGCAGTTTTCGACTTCTAAAAGCAAGTCATACCCGTCTGTCCAGGTAATTCTATTGTTTACCTCA
Poc 16 CC	TGGGGGACTCCCTGGGCAGTTTTCGACTTCTAAAAGCAAGTCATACCCGTCTGTCCAGGTAATTCTATTGTTTACCTCA
Poc 17 CC	TGGGGGACTCCCTGGGCAGTTTTCGACTTCTAAAAGCAAGTCATACCCGTCTGTCCAGGTAATTCTATTGTTTACCTCA
Poc 21 CC	TGGGGGACTCCCTGGGCAGTTTTCGACTTCTAAAAGCAAGTCATACCCGTCTGTCCAGGTAATTCTATTGTTTACCTCA
Poc 22 CC	TGGGGGACTCCCTGGGCAGTTTTCGACTTCTAAAAGCAAGTCATACCCGTCTGTCCAGGTAATTCTATTGTTTACCTCA

Sample	81–159
Sipp1 CL1 CC	TGGGGGACTCCCTGGGCAGTTTTCGACTTCTAAAAGCAAGTCATACCCGTCTGTCCAGGTAATTCTATTGTTTACCTCA
Sipp 1 Cl2 CC	TGGGGGACTCCCTGGGCAGTTTTCGACTTCTAAAAGCAAGTCATACCCGTCTGTCCAGGTAATTCTATTGTTTACCTCA
Sipp4 CL1 CC	TGGGGGACTCCCTGGGCAGTTTTCGACTTCTAAAAGCAAGTCATACCCGTCTGTCCAGGTAATTCTATTGTTTACCTCA
Sipp04 2 1 CC	TGGGGGACTCCCTGGGCAGTTTTCGACTTCTAAAAGCAAGTCATACCCGTCTGTCCAGGTAATTCTATTGTTTACCTCA
Sipp04 2 5 CC	TGGGGGACTCCCTGGGCAGTTTTCGACTTCTAAAAGCAAGTCATACCCGTCTGTCCAGGTAATTCTATTGTTTACCTCA
Sipp04 2 7 CC	TGGGGGACTCCCTGGGCAGTTTTCGACTTCTAAAAGCAAGTCATACCCGTCTGTCCAGGTAATTCTATTGTTTACCTCA
Sipp04 2 9 CC	TGGGGGACTCCCTGGGCAGTTTTCGACTTCTAAAAGCAAGTCATACCCGTCTGTCCAGGTAATTCTATTGTTTACCTCA
Sipp04 2 11 CC	TGGGGGACTCCCTGGGCAGTTTTCGACTTCTAAAAGCAAGTCATACCCGTCTGTCCAGGTAATTCTATTGTTTACCTCA
Sipp04 2 14 CC	TGGGGGACTCCCTGGGCAGTTTTCGACTTCTAAAAGCAAGTCATACCCGTCTGTCCAGGTAATTCTATTGTTTACCTCA
Sipp04 2 17 CC	TGGGGGACTCCCTGGGCAGTTTTCGACTTCTAAAAGCAAGTCATACCCGTCTGTCCAGGTAATTCTATTGTTTACCTCA
Sipp04 3 1 CC	TGGGGGACTCCCTGGGCAGTTTTCGACTTCTAAAAGCAAGTCATACCCGTCTGTCCAGGTAATTCTATTGTTTACCTCA
Sipp04 3 3 CC	TGGGGGACTCCCTGGGCAGTTTTCGACTTCTAAAAGCAAGTCATACCCGTCTGTCCAGGTAATTCTATTGTTTACCTCA
Sipp04 3 5 CC	TGGGGGACTCCCTGGGCAGTTTTCGACTTCTAAAAGCAAGTCATACCCGTCTGTCCAGGTAATTCTATTGTTTACCTCA
Sipp04 3 6 CC	TGGGGGACTCCCTGGGCAGTTTTCGACTTCTAAAAGCAAGTCATACCCGTCTGTCCAGGTAATTCTATTGTTTACCTCA
Sipp 13 15 CC	TGGGGGACTCCCTGGGCAGTTTTCGACTTCTAAAAGCAAGTCATACCCGTCTGTCCAGGTAATTCTATTGTTTACCTCA
Sipp 14 1 CC	TGGGGGACTCCCTGGGCAGTTTTCGACTTCTAAAAGCAAGTCATACCCGTCTGTCCAGGTAATTCTATTGTTTACCTCA
Sipp 14 2 CC	TGGGGGACTCCCTGGGCAGTTTTCGACTTCTAAAAGCAAGTCATACCCGTCTGTCCAGGTAATTCTATTGTTTACCTCA
Sipp 14 3 CC	TGGGGGACTCCCTGGGCAGTTTTCGACTTCTAAAAGCAAGTCATACCCGTCTGTCCAGGTAATTCTATTGTTTACCTCA
Sipp 14 4 CC	TGGGGGACTCCCTGGGCAGTTTTCGACTTCTAAAAGCAAGTCATACCCGTCTGTCCAGGTAATTCTATTGTTTACCTCA
Sipp 14 5 CC	TGGGGGACTCCCTGGGCAGTTTTCGACTTCTAAAAGCAAGTCATACCCGTCTGTCCAGGTAATTCTATTGTTTACCTCA
Sipp 14 6 CC	TGGGGGACTCCCTGGGCAGTTTTCGACTTCTAAAAGCAAGTCATACCCGTCTGTCCAGGTAATTCTATTGTTTACCTCA
Sipp 14 7 CC	TGGGGGACTCCCTGGGCAGTTTTCGACTTCTAAAAGCAAGTCATACCCGTCTGTCCAGGTAATTCTATTGTTTACCTCA
Sipp 14 8 CC	TGGGGGACTCCCTGGGCAGTTTTCGACTTCTAAAAGCAAGTCATACCCGTCTGTCCAGGTAATTCTATTGTTTACCTCA
Sipp 14 9 CC	TGGGGGACTCCCTGGGCAGTTTTCGACTTCTAAAAGCAAGTCATACCCGTCTGTCCAGGTAATTCTATTGTTTACCTCA
Sipp 14 10 CC	TGGGGGACTCCCTGGGCAGTTTTCGACTTCTAAAAGCAAGTCATACCCGTCTGTCCAGGTAATTCTATTGTTTACCTCA
Sipp 14 11 CC	TGGGGGACTCCCTGGGCAGTTTTCGACTTCTAAAAGCAAGTCATACCCGTCTGTCCAGGTAATTCTATTGTTTACCTCA
Sipp 14 12 CC	TGGGGGACTCCCTGGGCAGTTTTCGACTTCTAAAAGCAAGTCATACCCGTCTGTCCAGGTAATTCTATTGTTTACCTCA
Sipp 14 13 CC	TGGGGGACTCCCTGGGCAGTTTTCGACTTCTAAAAGCAAGTCATACCCGTCTGTCCAGGTAATTCTATTGTTTACCTCA
Sipp 14 14 CC	TGGGGGACTCCCTGGGCAGTTTTCGACTTCTAAAAGCAAGTCATACCCGTCTGTCCAGGTAATTCTATTGTTTACCTCA
Sipp 14 15 CC	TGGGGGACTCCCTGGGCAGTTTTCGACTTCTAAAAGCAAGTCATACCCGTCTGTCCAGGTAATTCTATTGTTTACCTCA
Sipp 15 1 CC	TGGGGGACTCCCTGGGCAGTTTTCGACTTCTAAAAGCAAGTCATACCCGTCTGTCCAGGTAATTCTATTGTTTACCTCA
Sipp 15 2 CC	TGGGGGACTCCCTGGGCAGTTTTCGACTTCTAAAAGCAAGTCATACCCGTCTGTCCAGGTAATTCTATTGTTTACCTCA
Sipp 15 3 CC	TGGGGGACTCCCTGGGCAGTTTTCGACTTCTAAAAGCAAGTCATACCCGTCTGTCCAGGTAATTCTATTGTTTACCTCA
Sipp 15 4 CC	TGGGGGACTCCCTGGGCAGTTTTCGACTTCTAAAAGCAAGTCATACCCGTCTGTCCAGGTAATTCTATTGTTTACCTCA
Sipp 15 5 CC	TGGGGGACTCCCTGGGCAGTTTTCGACTTCTAAAAGCAAGTCATACCCGTCTGTCCAGGTAATTCTATTGTTTACCTCA
Sipp 15 6 CC	TGGGGGACTCCCTGGGCAGTTTTCGACTTCTAAAAGCAAGTCATACCCGTCTGTCCAGGTAATTCTATTGTTTACCTCA
Sipp 15 7 CC	TGGGGGACTCCCTGGGCAGTTTTCGACTTCTAAAAGCAAGTCATACCCGTCTGTCCAGGTAATTCTATTGTTTACCTCA
Sipp 15 8 CC	TGGGGGACTCCCTGGGCAGTTTTCGACTTCTAAAAGCAAGTCATACCCGTCTGTCCAGGTAATTCTATTGTTTACCTCA
Sipp 15 9 CC	TGGGGGACTCCCTGGGCAGTTTTCGACTTCTAAAAGCAAGTCATACCCGTCTGTCCAGGTAATTCTATTGTTTACCTCA
Sipp 15 10 CC	TGGGGGACTCCCTGGGCAGTTTTCGACTTCTAAAAGCAAGTCATACCCGTCTGTCCAGGTAATTCTATTGTTTACCTCA
Sipp 15 11 CC	TGGGGGACTCCCTGGGCAGTTTTCGACTTCTAAAAGCAAGTCATACCCGTCTGTCCAGGTAATTCTATTGTTTACCTCA
Sipp 15 12 CC	TGGGGGACTCCCTGGGCAGTTTTCGACTTCTAAAAGCAAGTCATACCCGTCTGTCCAGGTAATTCTATTGTTTACCTCA
Sipp 15 13 CC	TGGGGGACTCCCTGGGCAGTTTTCGACTTCTAAAAGCAAGTCATACCCGTCTGTCCAGGTAATTCTATTGTTTACCTCA
Sipp 15 14 CC	TGGGGGACTCCCTGGGCAGTTTTCGACTTCTAAAAGCAAGTCATACCCGTCTGTCCAGGTAATTCTATTGTTTACCTCA
Sipp 15 15 CC	TGGGGGACTCCCTGGGCAGTTTTCGACTTCTAAAAGCAAGTCATACCCGTCTGTCCAGGTAATTCTATTGTTTACCTCA
Sipp 16 1 CS	TGGGGGACTCCCTGGGCAGTTTTCGACTTCTAAAAGCAAGTCATACCCGTCTGTCCAGGTAATTCTATTGTTTACCTSA
Sipp 16 2 CS	TGGGGGACTCCCTGGGCAGTTTTCGACTTCTAAAAGCAAGTCATACCCGTCTGTCCAGGTAATTCTATTGTTTACCTSA
Sipp 16 3 CS	TGGGGGACTCCCTGGGCAGTTTTCGACTTCTAAAAGCAAGTCATACCCGTCTGTCCAGGTAATTCTATTGTTTACCTSA
Sipp 16 8 CC	TGGGGGACTCCCTGGGCAGTTTTCGACTTCTAAAAGCAAGTCATACCCGTCTGTCCAGGTAATTCTATTGTTTACCTCA
NJ CL2 CC	TGGGGGACTCCCTGGGCAGTTTTCGACTTCTAAAAGCAAGTCATACCCGTCTGTCCAGGTAATTCTATTGTTTACCTCA
NJ CL3 CC	TGGGGGACTCCCTGGGCAGTTTTCGACTTCTAAAAGCAAGTCATACCCGTCTGTCCAGGTAATTCTATTGTTTACCTCA

	81 90 100 110 120 130 140 150 159
NJM 1 4 CC	TGGGGGACTCCCTGGGCAGTTTTCGACTTCTAAAAGCAAGTCATACCCGTCTGTCCAGGTAATTCTATTGTTTACCTCA
NJM 1 5 CC	TGGGGGACTCCCTGGGCAGTTTTCGACTTCTAAAAGCAAGTCATACCCGTCTGTCCAGGTAATTCTATTGTTTACCTCA
NJM 1 8 CC	TGGGGGACTCCCTGGGCAGTTTTCGACTTCTAAAAGCAAGTCATACCCGTCTGTCCAGGTAATTCTATTGTTTACCTCA
NJM 1 9 CC	TGGGGGACTCCCTGGGCAGTTTTCGACTTCTAAAAGCAAGTCATACCCGTCTGTCCAGGTAATTCTATTGTTTACCTCA
NJM 1 10 CC	TGGGGGACTCCCTGGGCAGTTTTCGACTTCTAAAAGCAAGTCATACCCGTCTGTCCAGGTAATTCTATTGTTTACCTCA
NJM 1 11 CC	TGGGGGACTCCCTGGGCAGTTTTCGACTTCTAAAAGCAAGTCATACCCGTCTGTCCAGGTAATTCTATTGTTTACCTCA
Rh1 CS	TGGGGGACTCCCTGGGCAGTTTTCGACTTCTAAAAGCAAGTCATACCCGTCTGTCCAGGTAATTCTATTGTTTACCTSA
Rh3 SS	TGGGGGACTCCCTGGGCAGTTTTCGACTTCTAAAAGCAAGTCATACCCGTCTGTCCAGGTAATTCTATTGTTTACCTGA
Rh5 SS	TGGGGGACTCCCTGGGCAGTTTTCGACTTCTAAAAGCAAGTCATACCCGTCTGTCCAGGTAATTCTATTGTTTACCTGA
Rh6 CS	TGGGGGACTCCCTGGGCAGTTTTCGACTTCTAAAAGCAAGTCATACCCGTCTGTCCAGGTAATTCTATTGTTTACCTSA
Rh8 CS	TGGGGGACTCCCTGGGCAGTTTTCGACTTCTAAAAGCAAGTCATACCCGTCTGTCCAGGTAATTCTATTGTTTACCTSA
Rh9 CC	TGGGGGACTCCCTGGGCAGTTTTCGACTTCTAAAAGCAAGTCATACCCGTCTGTCCAGGTAATTCTATTGTTTACCTCA
Rh10 CC	TGGGGGACTCCCTGGGCAGTTTTCGACTTCTAAAAGCAAGTCATACCCGTCTGTCCAGGTAATTCTATTGTTTACCTCA
Rh11 CS	TGGGGGACTCCCTGGGCAGTTTTCGACTTCTAAAAGCAAGTCATACCCGTCTGTCCAGGTAATTCTATTGTTTACCTSA
Rh12 CS	TGGGGGACTCCCTGGGCAGTTTTCGACTTCTAAAAGCAAGTCATACCCGTCTGTCCAGGTAATTCTATTGTTTACCTSA
Rh13 CC	TGGGGGACTCCCTGGGCAGTTTTCGACTTCTAAAAGCAAGTCATACCCGTCTGTCCAGGTAATTCTATTGTTTACCTCA
Rh14 SS	TGGGGGACTCCCTGGGCAGTTTTCGACTTCTAAAAGCAAGTCATACCCGTCTGTCCAGGTAATTCTATTGTTTACCTGA
Rh15 SS	TGGGGGACTCCCTGGGCAGTTTTCGACTTCTAAAAGCAAGTCATACCCGTCTGTCCAGGTAATTCTATTGTTTACCTGA
Rh16 CC	TGGGGGACTCCCTGGGCAGTTTTCGACTTCTAAAAGCAAGTCATACCCGTCTGTCCAGGTAATTCTATTGTTTACCTCA
Rh17 CS	TGGGGGACTCCCTGGGCAGTTTTCGACTTCTAAAAGCAAGTCATACCCGTCTGTCCAGGTAATTCTATTGTTTACCTSA
Rh18 CS	TGGGGGACTCCCTGGGCAGTTTTCGACTTCTAAAAGCAAGTCATACCCGTCTGTCCAGGTAATTCTATTGTTTACCTSA
Rh19 CC	TGGGGGACTCCCTGGGCAGTTTTCGACTTCTAAAAGCAAGTCATACCCGTCTGTCCAGGTAATTCTATTGTTTACCTCA
Rh21 CC	TGGGGGACTCCCTGGGCAGTTTTCGACTTCTAAAAGCAAGTCATACCCGTCTGTCCAGGTAATTCTATTGTTTACCTCA
Rh23 CS	TGGGGGACTCCCTGGGCAGTTTTCGACTTCTAAAAGCAAGTCATACCCGTCTGTCCAGGTAATTCTATTGTTTACCTSA
Rh24 CS	TGGGGGACTCCCTGGGCAGTTTTCGACTTCTAAAAGCAAGTCATACCCGTCTGTCCAGGTAATTCTATTGTTTACCTSA
Rh1-1 SS	TGGGGGACTCCCTGGGCAGTTTTCGACTTCTAAAAGCAAGTCATACCCGTCTGTCCAGGTAATTCTATTGTTTACCTGA
Rh1-2 CS	TGGGGGACTCCCTGGGCAGTTTTCGACTTCTAAAAGCAAGTCATACCCGTCTGTCCAGGTAATTCTATTGTTTACCTSA
Rh1-3 CC	TGGGGGACTCCCTGGGCAGTTTTCGACTTCTAAAAGCAAGTCATACCCGTCTGTCCAGGTAATTCTATTGTTTACCTCA
Rh1-6 CC	TGGGGGACTCCCTGGGCAGTTTTCGACTTCTAAAAGCAAGTCATACCCGTCTGTCCAGGTAATTCTATTGTTTACCTCA
Rh1-7 CC	TGGGGGACTCCCTGGGCAGTTTTCGACTTCTAAAAGCAAGTCATACCCGTCTGTCCAGGTAATTCTATTGTTTACCTCA
Rh1-8 CS	TGGGGGACTCCCTGGGCAGTTTTCGACTTCTAAAAGCAAGTCATACCCGTCTGTCCAGGTAATTCTATTGTTTACCTSA
Rh1-9 SS	TGGGGGACTCCCTGGGCAGTTTTYGACTTCTAAAAGCAAGTCATACCCGTCTGTCCAGGTAATTCTATTGTTTACCTGA
Rh1-10 CC	TGGGGGACTCCCTGGGCAGTTTTCGACTTCTAAAAGCAAGTCATACCCGTCTGTCCAGGTAATTCTATTGTTTACCTCA
Rh1-11 CS	TGGGGGACTCCCTGGGCAGTTTTCGACTTCTAAAAGCAAGTCATACCCGTCTGTCCAGGTAATTCTATTGTTTACCTSA
Rh1-13 CS	TGGGGGACTCCCTGGGCAGTTTTCGACTTCTAAAAGCAAGTCATACCCGTCTGTCCAGGTAATTCTATTGTTTACCTSA
Rh1-14 CS	TGGGGGACTCCCTGGGCAGTTTTCGACTTCTAAAAGCAAGTCATACCCGTCTGTCCAGGTAATTCTATTGTTTACCTSA
Rh1-15 CS	TGGGGGACTCCCTGGGCAGTTTTCGACTTCTAAAAGCAAGTCATACCCGTCTGTCCAGGTAATTCTATTGTTTACCTSA
Rh1-17 CS	TGGGGGACTCCCTGGGCAGTTTTCGACTTCTAAAAGCAAGTCATACCCGTCTGTCCAGGTAATTCTATTGTTTACCTSA
Rh1-18 CC	TGGGGGACTCCCTGGGCAGTTTTCGACTTCTAAAAGCAAGTCATACCCGTCTGTCCAGGTAATTCTATTGTTTACCTCA
Rh1- 19 SS	TGGGGGACTCCCTGGGCAGTTTTCGACTTCTAAAAGCAAGTCATACCCGTCTGTCCAGGTAATTCTATTGTTTACCTGA
Rh1-20 CC	TGGGGGACTCCCTGGGCAGTTTTCGACTTCTAAAAGCAAGTCATACCCGTCTGTCCAGGTAATTCTATTGTTTACCTCA
Rh1-21 CS	TGGGGGACTCCCTGGGCAGTTTTCGACTTCTAAAAGCAAGTCATACCCGTCTGTCCAGGTAATTCTATTGTTTACCTSA
Rh1-23 CC	TGGGGGACTCCCTGGGCAGTTTTCGACTTCTAAAAGCAAGTCATACCCGTCTGTCCAGGTAATTCTATTGTTTACCTCA
Rh1-24 CC	TGGGGGACTCCCTGGGCAGTTTTCGACTTCTAAAAGCAAGTCATACCCGTCTGTCCAGGTAATTCTATTGTTTACCTCA
Rh1-27 CC	TGGGGGACTCCCTGGGCAGTTTTCGACTTCTAAAAGCAAGTCATACCCGTCTGTCCAGGTAATTCTATTGTTTACCTCA
Rh1-28 SS	TGGGGGACTCCCTGGGCAGTTTTCGACTTCTAAAAGCAAGTCATACCCGTCTGTCCAGGTAATTCTATTGTTTACCTGA
Rh1- 30 CC	TGGGGGACTCCCTGGGCAGTTTTCGACTTCTAAAAGCAAGTCATACCCGTCTGTCCAGGTAATTCTATTGTTTACCTCA
Rh1-31 CS	TGGGGGACTCCCTGGGCAGTTTTCGACTTCTAAAAGCAAGTCATACCCGTCTGTCCAGGTAATTCTATTGTTTACCTSA
Rh1-32 CC	TGGGGGACTCCCTGGGCAGTTTTCGACTTCTAAAAGCAAGTCATACCCGTCTGTCCAGGTAATTCTATTGTTTACCTCA
Rh1-33 CC	TGGGGGACTCCCTGGGCAGTTTTCGACTTCTAAAAGCAAGTCATACCCGTCTGTCCAGGTAATTCTATTGTTTACCTCA
Rh1-34 CC	TGGGGGACTCCCTGGGCAGTTTTCGACTTCTAAAAGCAAGTCATACCCGTCTGTCCAGGTAATTCTATTGTTTACCTCA

	81 90 100 110 120 130 140 150 159
Rh1-35 CS	TGGGGGACTCCCTGGGCAGTTTTCGACTTCTAAAAGCAAGTCATACCCGTCTGTCCAGGTAATTCTATTGTTTACCTSA
Rh1-36 CS	TGGGGGACTCCCTGGGCAGTTTTCGACTTCTAAAAGCAAGTCATACCCGTCTGTCCAGGTAATTCTATTGTTTACCTSA
Rh1-37 CS	TGGGGGACTCCCTGGGCAGTTTTCGACTTCTAAAAGCAAGTCATACCCGTCTGTCCAGGTAATTCTATTGTTTACCTSA
Rh1-38 CS	TGGGGGACTCCCTGGGCAGTTTTCGACTTCTAAAAGCAAGTCATACCCGTCTGTCCAGGTAATTCTATTGTTTACCTSA
Rh1-39 SS	TGGGGGACTCCCTGGGCAGTTTTCGACTTCTAAAAGCAAGTCATACCCGTCTGTCCAGGTAATTCTATTGTTTACCTGA
Rh1-40 CC	TGGGGGACTCCCTGGGCAGTTTTCGACTTCTAAAAGCAAGTCATACCCGTCTGTCCAGGTAATTCTATTGTTTACCTCA
Rh1-41 CC	TGGGGGACTCCCTGGGCAGTTTTCGACTTCTAAAAGCAAGTCATACCCGTCTGTCCAGGTAATTCTATTGTTTACCTCA
Rh1-43 SS	TGGGGGACTCCCTGGGCAGTTTTCGACTTCTAAAAGCAAGTCATACCCGTCTGTCCAGGTAATTCTATTGTTTACCTGA
Rh1-44 CC	TGGGGGACTCCCTGGGCAGTTTTCGACTTCTAAAAGCAAGTCATACCCGTCTGTCCAGGTAATTCTATTGTTTACCTCA
Rh1-47 CS	TGGGGGACTCCCTGGGCAGTTTTCGACTTCTAAAAGCAAGTCATACCCGTCTGTCCAGGTAATTCTATTGTTTACCTSA
Rh1-49 CS	TGGGGGACTCCCTGGGCAGTTTTCGACTTCTAAAAGCAAGTCATACCCGTCTGTCCAGGTAATTCTATTGTTTACCTSA
Rh1-52 CS	TGGGGGACTCCCTGGGCAGTTTTCGACTTCTAAAAGCAAGTCATACCCGTCTGTCCAGGTAATTCTATTGTTTACCTSA
Rh1-53 CS	TGGGGGACTCCCTGGGCAGTTTTCGACTTCTAAAAGCAAGTCATACCCGTCTGTCCAGGTAATTCTATTGTTTACCTSA
Rh1-54 CC	TGGGGGACTCCCTGGGCAGTTTTCGACTTCTAAAAGCAAGTCATACCCGTCTGTCCAGGTAATTCTATTGTTTACCTCA
Rh1-58 CS	TGGGGGACTCCCTGGGCAGTTTTCGACTTCTAAAAGCAAGTCATACCCGTCTGTCCAGGTAATTCTATTGTTTACCTSA
Rh1-60 SS	TGGGGGACTCCCTGGGCAGTTTTCGACTTCTAAAAGCAAGTCATACCCGTCTGTCCAGGTAATTCTATTGTTTACCTGA
Bar1a 3 CC	TGGGGGACTCCCTGGGCAGTTTTCGACTTCTAAAAGCAAGTCATACCCGTCTGTCCAGGTAATTCTATTGTTTACCTSA
Bar1a 4 CC	TGGGGGACTCCCTGGGCAGTTTTCGACTTCTAAAAGCAAGTCATACCCGTCTGTCCAGGTAATTCTATTGTTTACCTGA
Bar1a 5 CC	TGGGGGACTCCCTGGGCAGTTTTCGACTTCTAAAAGCAAGTCATACCCGTCTGTCCAGGTAATTCTATTGTTTACCTSA
Bar1a 6 CC	TGGGGGACTCCCTGGGCAGTTTTCGACTTCTAAAAGCAAGTCATACCCGTCTGTCCAGGTAATTCTATTGTTTACCTCA
Bar1a 8 CC	TGGGGGACTCCCTGGGCAGTTTTCGACTTCTAAAAGCAAGTCATACCCGTCTGTCCAGGTAATTCTATTGTTTACCTGA
Bar1a 9 CC	TGGGGGACTCCCTGGGCAGTTTTCGACTTCTAAAAGCAAGTCATACCCGTCTGTCCAGGTAATTCTATTGTTTACCTSA
Bar1a 10 CC	TGGGGGACTCCCTGGGCAGTTTTCGACTTCTAAAAGCAAGTCATACCCGTCTGTCCAGGTAATTCTATTGTTTACCTCA
Bar1a 11 CC	TGGGGGACTCCCTGGGCAGTTTTCGACTTCTAAAAGCAAGTCATACCCGTCTGTCCAGGTAATTCTATTGTTTACCTSA
Bar1a 13 CC	TGGGGGACTCCCTGGGCAGTTTTCGACTTCTAAAAGCAAGTCATACCCGTCTGTCCAGGTAATTCTATTGTTTACCTSA
Bar1a 14 CC	TGGGGGACTCCCTGGGCAGTTTTCGACTTCTAAAAGCAAGTCATACCCGTCTGTCCAGGTAATTCTATTGTTTACCTCA
Bar1a 15 CC	TGGGGGACTCCCTGGGCAGTTTTCGACTTCTAAAAGCAAGTCATACCCGTCTGTCCAGGTAATTCTATTGTTTACCTSA
Bar1a 16 CC	TGGGGGACTCCCTGGGCAGTTTTCGACTTCTAAAAGCAAGTCATACCCGTCTGTCCAGGTAATTCTATTGTTTACCTSA
Bar1a 17 CC	TGGGGGACTCCCTGGGCAGTTTTCGACTTCTAAAAGCAAGTCATACCCGTCTGTCCAGGTAATTCTATTGTTTACCTCA
Bar1a 18 CC	TGGGGGACTCCCTGGGCAGTTTTCGACTTCTAAAAGCAAGTCATACCCGTCTGTCCAGGTAATTCTATTGTTTACCTSA
Bar1a 19 CC	TGGGGGACTCCCTGGGCAGTTTTCGACTTCTAAAAGCAAGTCATACCCGTCTGTCCAGGTAATTCTATTGTTTACCTSA
Bar1c 1 CC	TGGGGGACTCCCTGGGCAGTTTTCGACTTCTAAAAGCAAGTCATACCCGTCTGTCCAGGTAATTCTATTGTTTACCTSA
Bar1c 2 CC	TGGGGGACTCCCTGGGCAGTTTTCGACTTCTAAAAGCAAGTCATACCCGTCTGTCCAGGTAATTCTATTGTTTACCTSA
Bar2 2 CC	TGGGGGACTCCCTGGGCAGTTTTCGACTTCTAAAAGCAAGTCATACCCGTCTGTCCAGGTAATTCTATTGTTTACCTCA
Bar2a 4 CC	TGGGGGACTCCCTGGGCAGTTTTCGACTTCTAAAAGCAAGTCATACCCGTCTGTCCAGGTAATTCTATTGTTTACCTCA
Bar2a 5 CC	TGGGGGACTCCCTGGGCAGTTTTCGACTTCTAAAAGCAAGTCATACCCGTCTGTCCAGGTAATTCTATTGTTTACCTSA
Bar2b 1 CC	TGGGGGACTCCCTGGGCAGTTTTCGACTTCTAAAAGCAAGTCATACCCGTCTGTCCAGGTAATTCTATTGTTTACCTCA
Bar2b 2 CC	TGGGGGACTCCCTGGGCAGTTTTCGACTTCTAAAAGCAAGTCATACCCGTCTGTCCAGGTAATTCTATTGTTTACCTSA
Bar2c 6 CC	TGGGGGACTCCCTGGGCAGTTTTCGACTTCTAAAAGCAAGTCATACCCGTCTGTCCAGGTAATTCTATTGTTTACCTSA
SJ1 4 CC	TGGGGGACTCCCTGGGCAGTTTTCGACTTCTAAAAGCAAGTCATACCCGTCTGTCCAGGTAATTCTATTGTTTACCTCA
SJ1 6 CC	TGGGGGACTCCCTGGGCAGTTTTCGACTTCTAAAAGCAAGTCATACCCGTCTGTCCAGGTAATTCTATTGTTTACCTCA
SJ1 7 CC	TGGGGGACTCCCTGGGCAGTTTTCGACTTCTAAAAGCAAGTCATACCCGTCTGTCCAGGTAATTCTATTGTTTACCTCA
SJ2 4 CC	TGGGGGACTCCCTGGGCAGTTTTCGACTTCTAAAAGCAAGTCATACCCGTCTGTCCAGGTAATTCTATTGTTTACCTCA
SJ2 5 CC	TGGGGGACTCCCTGGGCAGTTTTCGACTTCTAAAAGCAAGTCATACCCGTCTGTCCAGGTAATTCTATTGTTTACCTCA
SJ2 6 CC	TGGGGGACTCCCTGGGCAGTTTTCGACTTCTAAAAGCAAGTCATACCCGTCTGTCCAGGTAATTCTATTGTTTACCTCA
SJ2 7 CC	TGGGGGACTCCCTGGGCAGTTTTCGACTTCTAAAAGCAAGTCATACCCGTCTGTCCAGGTAATTCTATTGTTTACCTCA
SJ3 4 CC	TGGGGGACTCCCTGGGCAGTTTTCGACTTCTAAAAGCAAGTCATACCCGTCTGTCCAGGTAATTCTATTGTTTACCTCA
SJ3 5 CC	TGGGGGACTCCCTGGGCAGTTTTCGACTTCTAAAAGCAAGTCATACCCGTCTGTCCAGGTAATTCTATTGTTTACCTCA
SJ3 6 CC	TGGGGGACTCCCTGGGCAGTTTTCGACTTCTAAAAGCAAGTCATACCCGTCTGTCCAGGTAATTCTATTGTTTACCTCA
SJ3 8 CC	TGGGGGACTCCCTGGGCAGTTTTCGACTTCTAAAAGCAAGTCATACCCGTCTGTCCAGGTAATTCTATTGTTTACCTCA
SJ25 3 CC	TGGGGGACTCCCTGGGCAGTTTTCGACTTCTAAAAGCAAGTCATACCCGTCTGTCCAGGTAATTCTATTGTTTACCTCA

Sample	81–159
Coos 3 CC	TGGGGGACTCCCTGGGCAGTTTTCGACTTCTAAAAGCAAGTCATACCCGTCTGTCCAGGTAATTCTATTGTTTACCTCA
CABC1 CC	TGGGGGACTCCCTGGGCAGTTTTCGACTTCTAAAAGCAAGTCATACCCGTCTGTCCAGGTAATTCTATTGTTTACCTCA
CABC2 CS	TGGGGGACTCCCTGGGCAGTTTTCGACTTCTAAAAGCAAGTCATACCCGTCTGTCCAGGTAATTCTATTGTTTACCTSA
CABC3 CC	TGGGGGACTCCCTGGGCAGTTTTCGACTTCTAAAAGCAAGTCATACCCGTCTGTCCAGGTAATTCTATTGTTTACCTCA
CABC4 CC	TGGGGGACTCCCTGGGCAGTTTTCGACTTCTAAAAGCAAGTCATACCCGTCTGTCCAGGTAATTCTATTGTTTACCTCA
CABC5 CS	TGGGGGACTCCCTGGGCAGTTTTCGACTTCTAAAAGCAAGTCATACCCGTCTGTCCAGGTAATTCTATTGTTTACCTSA
CASM1 CS	TGGGGGACTCCCTGGGCAGTTTTCGACTTCTAAAAGCAAGTCATACCCGTCTGTCCAGGTAATTCTATTGTTTACCTSA
CASM3 CS	TGGGGGACTCCCTGGGCAGTTTTCGACTTCTAAAAGCAAGTCATACCCGTCTGTCCAGGTAATTCTATTGTTTACCTSA
CASM9 CC	TGGGGGACTCCCTGGGCAGTTTTCGACTTCTAAAAGCAAGTCATACCCGTCTGTCCAGGTAATTCTATTGTTTACCTCA
CASM11 CC	TGGGGGACTCCCTGGGCAGTTTTCGACTTCTAAAAGCAAGTCATACCCGTCTGTCCAGGTAATTCTATTGTTTACCTCA
CASM13 CS	TGGGGGACTCCCTGGGCAGTTTTCGACTTCTAAAAGCAAGTCATACCCGTCTGTCCAGGTAATTCTATTGTTTACCTSA
CB5 CC	TGGGGGACTCCCTGGGCAGTTTTCGACTTCTAAAAGCAAGTCATACCCGTCTGTCCAGGTAATTCTATTGTTTACCTCA
CC2 6 CC	TGGGGGACTCCCTGGGCAGTTTTCGACTTCTAAAAGCAAGTCATACCCGTCTGTCCAGGTAATTCTATTGTTTACCTCA
CD 2 CC	TGGGGGACTCCCTGGGCAGTTTTCGACTTCTAAAAGCAAGTCATACCCGTCTGTCCAGGTAATTCTATTGTTTACCTCA
UK Gil 1 CL CC	TGGGGGACTCCCTGGGCAGTTTTCGACTTCTAAAAGCAAGTCATACCCGTCTGTCCAGGTAATTCTATTGTTTACCTCA
UK Gil 3 CC	TGGGGGACTCCCTGGGCAGTTTTCGACTTCTAAAAGCAAGTCATACCCGTCTGTCCAGGTAATTCTATTGTTTACCTCA
UK Gil 4 CC	TGGGGGACTCCCTGGGCAGTTTTCGACTTCTAAAAGCAAGTCATACCCGTCTGTCCAGGTAATTCTATTGTTTACCTCA
UK Gil 5 CC	TGGGGGACTCCCTGGGCAGTTTTCGACTTCTAAAAGCAAGTCATACCCGTCTGTCCAGGTAATTCTATTGTTTACCTCA
UK Gil 6 CC	TGGGGGACTCCCTGGGCAGTTTTCGACTTCTAAAAGCAAGTCATACCCGTCTGTCCAGGTAATTCTATTGTTTACCTCA
UK Gil 7 CC	TGGGGGACTCCCTGGGCAGTTTTCGACTTCTAAAAGCAAGTCATACCCGTCTGTCCAGGTAATTCTATTGTTTACCTCA
UK Gil 8 CC	TGGGGGACTCCCTGGGCAGTTTTCGACTTCTAAAAGCAAGTCATACCCGTCTGTCCAGGTAATTCTATTGTTTACCTCA
Gil 10 CC	TGGGGGACTCCCTGGGCAGTTTTCGACTTCTAAAAGCAAGTCATACCCGTCTGTCCAGGTAATTCTATTGTTTACCTCA
Gil 11 CC	TGGGGGACTCCCTGGGCAGTTTTCGACTTCTAAAAGCAAGTCATACCCGTCTGTCCAGGTAATTCTATTGTTTACCTCA
UK NrSalt 1 CC	TGGGGGACTCCCTGGGCAGTTTTCGACTTCTAAAAGCAAGTCATACCCGTCTGTCCAGGTAATTCTATTGTTTACCTCA
UK NrSalt 2 CC	TGGGGGACTCCCTGGGCAGTTTTCGACTTCTAAAAGCAAGTCATACCCGTCTGTCCAGGTAATTCTATTGTTTACCTCA
UK NrSalt 3 CC	TGGGGGACTCCCTGGGCAGTTTTCGACTTCTAAAAGCAAGTCATACCCGTCTGTCCAGGTAATTCTATTGTTTACCTCA
UK NrSalt 4 CC	TGGGGGACTCCCTGGGCAGTTTTCGACTTCTAAAAGCAAGTCATACCCGTCTGTCCAGGTAATTCTATTGTTTACCTCA
UK Nr Salt 5 CC	TGGGGGACTCCCTGGGCAGTTTTCGACTTCTAAAAGCAAGTCATACCCGTCTGTCCAGGTAATTCTATTGTTTACCTCA
UK Nr Salt 6 CC	TGGGGGACTCCCTGGGCAGTTTTCGACTTCTAAAAGCAAGTCATACCCGTCTGTCCAGGTAATTCTATTGTTTACCTCA
UK Nr Salt 7 CC	TGGGGGACTCCCTGGGCAGTTTTCGACTTCTAAAAGCAAGTCATACCCGTCTGTCCAGGTAATTCTATTGTTTACCTCA
UK Nr Salt 8 CC	TGGGGGACTCCCTGGGCAGTTTTCGACTTCTAAAAGCAAGTCATACCCGTCTGTCCAGGTAATTCTATTGTTTACCTCA
UK Nr Salt 9 CC	TGGGGGACTCCCTGGGCAGTTTTCGACTTCTAAAAGCAAGTCATACCCGTCTGTCCAGGTAATTCTATTGTTTACCTCA
UK NrSalt 10 CC	TGGGGGACTCCCTGGGCAGTTTTCGACTTCTAAAAGCAAGTCATACCCGTCTGTCCAGGTAATTCTATTGTTTACCTCA
UK NrSalt 11 CC	TGGGGGACTCCCTGGGCAGTTTTCGACTTCTAAAAGCAAGTCATACCCGTCTGTCCAGGTAATTCTATTGTTTACCTCA
UK NrSalt 13 CC	TGGGGGACTCCCTGGGCAGTTTTCGACTTCTAAAAGCAAGTCATACCCGTCTGTCCAGGTAATTCTATTGTTTACCTCA
UK NrSalt 14 CC	TGGGGGACTCCCTGGGCAGTTTTCGACTTCTAAAAGCAAGTCATACCCGTCTGTCCAGGTAATTCTATTGTTTACCTCA
UK NrSalt 15 CC	TGGGGGACTCCCTGGGCAGTTTTCGACTTCTAAAAGCAAGTCATACCCGTCTGTCCAGGTAATTCTATTGTTTACCTCA
UK Salt 1 CC	TGGGGGACTCCCTGGGCAGTTTTCGACTTCTAAAAGCAAGTCATACCCGTCTGTCCAGGTAATTCTATTGTTTACCTCA
UK Salt 3 CC	TGGGGGACTCCCTGGGCAGTTTTCGACTTCTAAAAGCAAGTCATACCCGTCTGTCCAGGTAATTCTATTGTTTACCTCA
UK Salt 4 CC	TGGGGGACTCCCTGGGCAGTTTTCGACTTCTAAAAGCAAGTCATACCCGTCTGTCCAGGTAATTCTATTGTTTACCTCA
UK Salt 5 CC	TGGGGGACTCCCTGGGCAGTTTTCGACTTCTAAAAGCAAGTCATACCCGTCTGTCCAGGTAATTCTATTGTTTACCTCA
UK Salt 6 CC	TGGGGGACTCCCTGGGCAGTTTTCGACTTCTAAAAGCAAGTCATACCCGTCTGTCCAGGTAATTCTATTGTTTACCTCA
UK Salt 7 CC	TGGGGGACTCCCTGGGCAGTTTTCGACTTCTAAAAGCAAGTCATACCCGTCTGTCCAGGTAATTCTATTGTTTACCTCA
UK Salt 8 CC	TGGGGGACTCCCTGGGCAGTTTTCGACTTCTAAAAGCAAGTCATACCCGTCTGTCCAGGTAATTCTATTGTTTACCTCA
UK Salt 9 CC	TGGGGGACTCCCTGGGCAGTTTTCGACTTCTAAAAGCAAGTCATACCCGTCTGTCCAGGTAATTCTATTGTTTACCTCA
UK Salt 10 CC	TGGGGGACTCCCTGGGCAGTTTTCGACTTCTAAAAGCAAGTCATACCCGTCTGTCCAGGTAATTCTATTGTTTACCTCA
