# Supplementary material for: Two Alleles of NF-κB in the Sea Anemone Nematostella vectensis Are Widely Dispersed in Nature and Encode Proteins with Distinct Activities
Source: PLoS One. 2009 Oct 6;4(10):e7311. doi: 10.1371/journal.pone.0007311 (PMC2751831; doi:10.1371/journal.pone.0007311)
Supplement: Figure S5 — Individual animals sequenced in the population genetic study. For each individual, the table lists the collection site, the sequences of the individual's two NF-κB alleles at the seven variable positions in the alignment (Allele 1 and Allele 2 SNPs), the posterior probability that these “best alleles” are the true alleles (Prob.), the name of each allele (Allele 1, Allele 2), and the genotype. (0.14 MB PDF) [file pone.0007311.s005.pdf]

Supplemental Figure 5. List of 403 individual animals sequenced in the population genetic study.

Collection Site	Individual	Allele 1 SNPs	Allele 2 SNPs	Prob.	Allele 1	Allele 2	Genotype
**Chezzetcook**	NS88-02CS	GGAGGTG	GTTTAAG	1.000	**C4**	**S1**	C4S1
**NOVA SCOTIA**	NS88-03CC	GGAGGTC	GGAGGTC	1.000	**C1**	**C1**	C1C1
[n=26]	NS88-04CS	GGAGGTC	GTTTAAG	1.000	**C1**	**S1**	C1S1
	NS296-03SS	GTTTAAG	GTTTAAG	1.000	**S1**	**S1**	S1S1
	NS296-07CS	GGAGGTC	GTTTAAG	1.000	**C1**	**S1**	C1S1
	NS296-08CS	GGAGGTC	GTTTAAG	1.000	**C1**	**S1**	C1S1
	NS296-09CS	GGAGGTG	GTTTAAG	1.000	**C4**	**S1**	C4S1
	NS296-11CS	GGAGGTG	GTTTAAG	1.000	**C4**	**S1**	C4S1
	NS296-12CS	GGAGGTC	GTTTAAG	1.000	**C1**	**S1**	C1S1
	Hal1-4CS	GGAGGTC	GTTTAAG	1.000	**C1**	**S1**	C1S1
	Hal1-5CS	GGAGGTC	GTTTAAG	1.000	**C1**	**S1**	C1S1
	Hal1-6CS	GGAGGTC	GTTTAAG	1.000	**C1**	**S1**	C1S1
	Hal1-7CS	GGAGGTC	GTTTAAG	1.000	**C1**	**S1**	C1S1
	Hal1-8CS	GGAGGTC	GTTTAAG	1.000	**C1**	**S1**	C1S1
	Hal1-9SS	GTTTAAG	GTTTAAG	1.000	**S1**	**S1**	S1S1
	Hal1-10CS	GGAGGTC	GTTTAAG	1.000	**C1**	**S1**	C1S1
	Hal1-11CS	GGAGGTC	GTTTAAG	1.000	**C1**	**S1**	C1S1
	Hal1-12CS	GGAGGTC	GTTTAAG	1.000	**C1**	**S1**	C1S1
	Hal1-14CS	GGAGGTC	GTTTAAG	1.000	**C1**	**S1**	C1S1
	Hal1-15CS	GGAGGTC	GTTTAAG	1.000	**C1**	**S1**	C1S1
	Hal1-16CS	GGAGGTC	GTTTAAG	1.000	**C1**	**S1**	C1S1
	Hal1-17CS	GGAGGTC	GTTTAAG	1.000	**C1**	**S1**	C1S1
	Hal1-19CS	GGAGGTC	GTTTAAG	1.000	**C1**	**S1**	C1S1
	Hal1-20CS	GGAGGTC	GTTTAAG	1.000	**C1**	**S1**	C1S1
	Hal1-21SS	GTTTAAG	GTTTAAG	1.000	**S1**	**S1**	S1S1
	Hal1-23CS	GGAGGTC	GTTTAAG	1.000	**C1**	**S1**	C1S1
**Peggy's Cove**	NS299-1SS	GTTTAAG	GTTTAAG	1.000	**S1**	**S1**	S1S1
**NOVA SCOTIA**	NS299-3SS	GTTTAAG	GTTTAAG	1.000	**S1**	**S1**	S1S1
[n=9]	NS299-4CC	GGAGGTC	GGAGGTC	1.000	**C1**	**C1**	C1C1
	NS299-5SS	GTTTAAG	GTTTAAG	1.000	**S1**	**S1**	S1S1
	NS299-6SS	GTTTAAG	GTTTAAG	1.000	**S1**	**S1**	S1S1
	NS299-8SS	GTTTAAG	GTTTAAG	1.000	**S1**	**S1**	S1S1
	NS299-9SS	GTTTAAG	GTTTAAG	1.000	**S1**	**S1**	S1S1
	NS299-10SS	GTTTAAG	GTTTAAG	1.000	**S1**	**S1**	S1S1
	NS299-11SS	GTTTAAG	GTTTAAG	1.000	**S1**	**S1**	S1S1
**Mahone Bay**	NS293-1CC	GGAGGTC	GGAGGTC	1.000	**C1**	**C1**	C1C1
**NOVA SCOTIA**	NS293-2CC	GGAGGTC	GGAGGTC	1.000	**C1**	**C1**	C1C1
[n=19]	NS293-3CC	GGAGGTC	GGAGGTC	1.000	**C1**	**C1**	C1C1
	NS293-4CC	GGAGGTC	GGAGGTC	1.000	**C1**	**C1**	C1C1
	NS293-5CC	GGAGGTC	GGAGGTC	1.000	**C1**	**C1**	C1C1
	NS293-6CC	GGAGGTC	GGAGGTC	1.000	**C1**	**C1**	C1C1
	NS293-7CC	GGAGGTC	GGAGGTC	1.000	**C1**	**C1**	C1C1
	NS293-8CC	GGAGGTC	GGAGGTC	1.000	**C1**	**C1**	C1C1
	NS293-9CC	GGAGGTC	GGAGGTC	1.000	**C1**	**C1**	C1C1
	NS293-10CC	GGAGGTC	GGAGGTC	1.000	**C1**	**C1**	C1C1
	NS293-11CC	GGAGGTC	GGAGGTC	1.000	**C1**	**C1**	C1C1

Supplemental Figure 5. List of 403 individual animals sequenced in the population genetic study.

Collection Site	Individual	Allele 1 SNPs	Allele 2 SNPs	Prob.	Allele 1	Allele 2	Genotype
	NS294-1CC	GGAGGTC	GGAGGTC	1.000	**C1**	**C1**	C1C1
	NS294-2CC	GGAGGTC	GGAGGTC	1.000	**C1**	**C1**	C1C1
	NS294-4CC	GGAGGTC	GGAGGTG	1.000	**C1**	**C4**	C1C4
	NS294-5CC	GGAGGTC	GGAGGTC	1.000	**C1**	**C1**	C1C1
	NS294-6CC	GGAGGTC	GGAGGTC	1.000	**C1**	**C1**	C1C1
	NS294-7CC	GGAGGTC	GGAGGTC	1.000	**C1**	**C1**	C1C1
	NS294-9CC	GGAGGTC	GGAGGTC	1.000	**C1**	**C1**	C1C1
	NS294-10CC	GGAGGTC	GGAGGTC	1.000	**C1**	**C1**	C1C1
**Crescent**	NS291-3CS	GGAGGTG	GTTTAAG	1.000	**C4**	**S1**	C4S1
**Beach**	NS291-4CC	GGAGGTC	GGAGGTC	1.000	**C1**	**C1**	C1C1
**NOVA SCOTIA**	NS291-5CS	GGAGGTC	GTTTAAG	1.000	**C1**	**S1**	C1S1
[n=16]	NS291-6CS	GGAGGTC	GTTTAAG	1.000	**C1**	**S1**	C1S1
	NS291-7CS	GGAGGTC	GTTTAAG	1.000	**C1**	**S1**	C1S1
	NS291-8CS	GGAGGTC	GTTTAAG	1.000	**C1**	**S1**	C1S1
	NS291-10CS	GGAGGTC	GTTTAAG	1.000	**C1**	**S1**	C1S1
	NS291-11CS	GGAGGTC	GTTTAAG	1.000	**C1**	**S1**	C1S1
	NS291-12CS	GGAGGTC	GTTTAAG	1.000	**C1**	**S1**	C1S1
	NS291-13CS	GGAGGTC	GTTTAAG	1.000	**C1**	**S1**	C1S1
	NS291-15CS	GGAGGTC	GTTTAAG	1.000	**C1**	**S1**	C1S1
	NS291-17CS	GGAGGTC	GTTTAAG	1.000	**C1**	**S1**	C1S1
	NS291-18CS	GGAGGTC	GTTTAAG	1.000	**C1**	**S1**	C1S1
	NS291-19CC	GGAGGTC	GGAGGTC	1.000	**C1**	**C1**	C1C1
	NS291-20CS	GGAGGTC	GTTTAAG	1.000	**C1**	**S1**	C1S1
	NS291-21CS	GGAGGTC	GTTTAAG	1.000	**C1**	**S1**	C1S1
**Noel**	NS298-1CC	GGAGGTC	GGAGGTC	1.000	**C1**	**C1**	C1C1
**NOVA SCOTIA**	NS298-2CC	GGAGGTC	GGAGGTC	1.000	**C1**	**C1**	C1C1
[n=10]	NS298-3CC	GGAGGTC	GGAGGTC	1.000	**C1**	**C1**	C1C1
	NS298-4SS	GTTTAAG	GTTTAAG	1.000	**S1**	**S1**	S1S1
	NS298-5CC	GGAGGTC	GGAGGTC	1.000	**C1**	**C1**	C1C1
	NS298-6CC	GGAGGTC	GGAGGTC	1.000	**C1**	**C1**	C1C1
	NS298-7CC	GGAGGTC	GGAGGTC	1.000	**C1**	**C1**	C1C1
	NS298-8CC	GGAGGTC	GGAGGTC	1.000	**C1**	**C1**	C1C1
	NS298-9CC	GGAGGTC	GGAGGTC	1.000	**C1**	**C1**	C1C1
	NS298-10CC	GGAGGTC	GGAGGTC	1.000	**C1**	**C1**	C1C1
**Kingsport**	KNG1-1CC	GGAGGTC	GGAGGTC	1.000	**C1**	**C1**	C1C1
**NOVA SCOTIA**	KNG1-2CC	GGAGGTC	AGAGGTC	1.000	**C1**	**C2**	C1C2
[n=35]	KNG1-3CC	GGAGGTC	AGAGGTC	1.000	**C1**	**C2**	C1C2
	KNG1-4CC	GGAGGTC	AGAGGTC	1.000	**C1**	**C2**	C1C2
	KNG1-5CC	GGAGGTC	GGAGGTC	1.000	**C1**	**C1**	C1C1
	KNG1-6CC	GGAGGTC	AGAGGTC	1.000	**C1**	**C2**	C1C2
	KNG1-7CC	GGAGGTC	AGAGGTC	1.000	**C1**	**C2**	C1C2
	KNG1-8CC	GGAGGTC	AGAGGTC	1.000	**C1**	**C2**	C1C2
	KNG1-9CC	GGAGGTC	AGAGGTC	1.000	**C1**	**C2**	C1C2
	KNG1-11CC	GGAGGTC	AGAGGTC	1.000	**C1**	**C2**	C1C2
	KNG1-12CC	GGAGGTC	AGAGGTC	1.000	**C1**	**C2**	C1C2
	KNG1-13CC	GGAGGTC	AGAGGTC	1.000	**C1**	**C2**	C1C2

Supplemental Figure 5. List of 403 individual animals sequenced in the population genetic study.

Collection Site	Individual	Allele 1 SNPs	Allele 2 SNPs	Prob.	Allele 1	Allele 2	Genotype
	KNG2-1CC	GGAGGTC	AGAGGTC	1.000	**C1**	**C2**	C1C2
	KNG2-2CC	GGAGGTC	GGAGGTC	1.000	**C1**	**C1**	C1C1
	KNG2-5CC	GGAGGTC	AGAGGTC	1.000	**C1**	**C2**	C1C2
	KNG2-6CC	GGAGGTC	GGAGGTC	1.000	**C1**	**C1**	C1C1
	KNG2-7CC	GGAGGTC	GGAGGTC	1.000	**C1**	**C1**	C1C1
	KNG2-8CC	GGAGGTC	GGAGGTC	1.000	**C1**	**C1**	C1C1
	KNG2-9CC	GGAGGTC	GGAGGTC	1.000	**C1**	**C1**	C1C1
	KNG3-2CC	GGAGGTC	GGAGGTC	1.000	**C1**	**C1**	C1C1
	KNG3-3CC	GGAGGTC	AGAGGTC	1.000	**C1**	**C2**	C1C2
	KNG3-4CC	GGAGGTC	AGAGGTC	1.000	**C1**	**C2**	C1C2
	KNG3-5CC	GGAGGTC	GGAGGTC	1.000	**C1**	**C1**	C1C1
	KNG3-6CC	GGAGGTC	GGAGGTC	1.000	**C1**	**C1**	C1C1
	KNG3-7CC	GGAGGTC	GGAGGTC	1.000	**C1**	**C1**	C1C1
	KNG3-8CC	GGAGGTC	GGAGGTC	1.000	**C1**	**C1**	C1C1
	KNG3-9CC	GGAGGTC	GGAGGTC	1.000	**C1**	**C1**	C1C1
	KNG3-10CC	GGAGGTC	GGAGGTC	1.000	**C1**	**C1**	C1C1
	KNG3-14CC	GGAGGTC	GGAGGTG	1.000	**C1**	**C4**	C1C4
	NSC1CC	GGAGGTC	GGAGGTC	1.000	**C1**	**C1**	C1C1
	NSC2CC	GGAGGTC	GGAGGTC	1.000	**C1**	**C1**	C1C1
	NSC3CC	GGAGGTC	GGAGGTC	1.000	**C1**	**C1**	C1C1
	NSC4CC	GGAGGTC	GGAGGTC	1.000	**C1**	**C1**	C1C1
	NSC6CC	GGAGGTC	GGAGGTC	1.000	**C1**	**C1**	C1C1
	NSC9CC	GGAGGTC	GGAGGTC	1.000	**C1**	**C1**	C1C1
**Odiorne Point**	OD1CC	GGAGGTC	GGAGGTC	1.000	**C1**	**C1**	C1C1
**NEW HAMPSHIRE**	OD2CC	GGAGGTC	GGAGGTC	1.000	**C1**	**C1**	C1C1
[n=12]	OD3CC	GGAGGTC	GGAGGTC	1.000	**C1**	**C1**	C1C1
	OD4CC	GGAGGTC	GGAGGTC	1.000	**C1**	**C1**	C1C1
	OD5CC	GGAGGTC	GGAGGTC	1.000	**C1**	**C1**	C1C1
	OD9CC	GGAGGTC	GGAGGTC	1.000	**C1**	**C1**	C1C1
	OD04-3CC	GGAGGTC	GGAGGTC	1.000	**C1**	**C1**	C1C1
	OD04-4CC	GGAGGTC	GGAGGTC	1.000	**C1**	**C1**	C1C1
	OD04-6CC	GGAGGTC	GGAGGTC	1.000	**C1**	**C1**	C1C1
	OD04-7CC	GGAGGTC	GGAGGTC	1.000	**C1**	**C1**	C1C1
	OD04-10CC	GGAGGTC	GGAGGTC	1.000	**C1**	**C1**	C1C1
	OD04-14CC	GGAGGTC	GGAGGTC	1.000	**C1**	**C1**	C1C1
**Rye Harbor**	RH4CC	GGAGGTC	GGAGGTC	1.000	**C1**	**C1**	C1C1
**NEW HAMPSHIRE**	RH5CC	GGAGGTC	GGAGGTC	1.000	**C1**	**C1**	C1C1
[n=6]	RH6CC	GGAGGTC	GGAGGTC	1.000	**C1**	**C1**	C1C1
	RH7CC	GGAGGTC	GGAGGTC	1.000	**C1**	**C1**	C1C1
	RH9CC	GGAGGTC	GGAGGTC	1.000	**C1**	**C1**	C1C1
	RH10CC	GGAGGTC	GGAGGTC	1.000	**C1**	**C1**	C1C1
**Wallis Sands**	WS1-1-2CC	GGAGGTC	GGAGGTC	1.000	**C1**	**C1**	C1C1
**NEW HAMPSHIRE**	WS1-1-4CC	GGAGGTC	GGAGGTC	1.000	**C1**	**C1**	C1C1
[n=21]	WS1-1-5CC	GGAGGTC	GGAGGTC	1.000	**C1**	**C1**	C1C1
	WS1-1-6CC	GGAGGTC	GGAGGTC	1.000	**C1**	**C1**	C1C1
	WS1-1-9CC	GGAGGTC	GGAGGTC	1.000	**C1**	**C1**	C1C1

Supplemental Figure 5. List of 403 individual animals sequenced in the population genetic study.

Collection Site	Individual	Allele 1 SNPs	Allele 2 SNPs	Prob.	Allele 1	Allele 2	Genotype
	WS1-1-10CC	GGAGGTC	GGAGGTC	1.000	**C1**	**C1**	C1C1
	WS1-1-11CC	GGAGGTC	GGAGGTC	1.000	**C1**	**C1**	C1C1
	WS1-1-12CC	GGAGGTC	GGAGGTC	1.000	**C1**	**C1**	C1C1
	WS1-1-16CC	GGAGGTC	GGAGGTC	1.000	**C1**	**C1**	C1C1
	WS1-1-17CC	GGAGGTC	GGAGGTC	1.000	**C1**	**C1**	C1C1
	WS1-1-18CC	GGAGGTC	GGAGGTC	1.000	**C1**	**C1**	C1C1
	WS1-1-19CC	GGAGGTC	GGAGGTC	1.000	**C1**	**C1**	C1C1
	WS1-1-20CC	GGAGGTC	GGAGGTC	1.000	**C1**	**C1**	C1C1
	WS1-2-2CC	GGAGGTC	GGAGGTC	1.000	**C1**	**C1**	C1C1
	WS1-2-3CC	GGAGGTC	GGAGGTC	1.000	**C1**	**C1**	C1C1
	WS1-2-4CC	GGAGGTC	GGAGGTC	1.000	**C1**	**C1**	C1C1
	WS1-2-5CC	GGAGGTC	GGAGGTC	1.000	**C1**	**C1**	C1C1
	WS1-2-7CC	GGAGGTC	GGAGGTC	1.000	**C1**	**C1**	C1C1
	WS2-1CC	GGAGGTC	GGAGGTC	1.000	**C1**	**C1**	C1C1
	WS2-2CC	GGAGGTC	GGAGGTC	1.000	**C1**	**C1**	C1C1
	WS2-3CC	GGAGGTC	GGAGGTC	1.000	**C1**	**C1**	C1C1
**Crane**	Cr4CC	GGAGGTC	GGAGGTC	1.000	**C1**	**C1**	C1C1
**MASSACHUSETTS**	Cr04-0CC	GGAGGTC	GGAGGTC	1.000	**C1**	**C1**	C1C1
[n=12]	Cr04-1CC	GGAGGTC	GGAGGTC	1.000	**C1**	**C1**	C1C1
	Cr04-2CC	GGAGGTC	GGAGGTC	1.000	**C1**	**C1**	C1C1
	Cr04-3CC	GGAGGTC	GGAGGTC	1.000	**C1**	**C1**	C1C1
	Cr04-4CC	GGAGGTC	GGAGGTC	1.000	**C1**	**C1**	C1C1
	Cr04-8CC	GGAGGTC	GGAGGTC	1.000	**C1**	**C1**	C1C1
	Cr04-10CC	GGAGGTC	GGAGGTC	1.000	**C1**	**C1**	C1C1
	Cr04-11CC	GGAGGTC	GGAGGTC	1.000	**C1**	**C1**	C1C1
	Cr04-12CC	GGAGGTC	GGAGGTC	1.000	**C1**	**C1**	C1C1
	Cr04-13CC	GGAGGTC	GGAGGTC	1.000	**C1**	**C1**	C1C1
	Cr04-14	GGAGGTC	GGAGGTC	1.000	**C1**	**C1**	C1C1
**Neponset**	Ne04-3CC	GGAGGTC	GGAGGTC	1.000	**C1**	**C1**	C1C1
**MASSACHUSETTS**	Ne04-5CC	GGAGGTC	GGAGGTC	1.000	**C1**	**C1**	C1C1
[n=23]	Ne04-6CC	GGAGGTC	GGAGGTC	1.000	**C1**	**C1**	C1C1
	Ne04-8CC	GGAGGTC	GGAGGTC	1.000	**C1**	**C1**	C1C1
	Ne04-9CC	GGAGGTC	GGAGGTC	1.000	**C1**	**C1**	C1C1
	Ne04-10CC	GGAGGTC	GGAGGTC	1.000	**C1**	**C1**	C1C1
	Ne04-11CC	GGAGGTC	GGAGGTC	1.000	**C1**	**C1**	C1C1
	Ne04-12CC	GGAGGTC	GGAGGTC	1.000	**C1**	**C1**	C1C1
	Ne04-14CC	GGAGGTC	GGAGGTC	1.000	**C1**	**C1**	C1C1
	Ne04-16CC	GGAGGTC	GGAGGTC	1.000	**C1**	**C1**	C1C1
	Ne04-17CC	GGAGGTC	GGAGGTC	1.000	**C1**	**C1**	C1C1
	Ne04-18CC	GGAGGTC	GGAGGTC	1.000	**C1**	**C1**	C1C1
	Ne04-19CC	GGAGGTC	GGAGGTC	1.000	**C1**	**C1**	C1C1
	Ne04-20CC	GGAGGTC	GGAGGTC	1.000	**C1**	**C1**	C1C1
	Ne04-21CC	GGAGGTC	GGAGGTC	1.000	**C1**	**C1**	C1C1
	Ne04-22CC	GGAGGTC	GGAGGTC	1.000	**C1**	**C1**	C1C1
	Ne04-23CC	GGAGGTC	GGAGGTC	1.000	**C1**	**C1**	C1C1
	Ne04-24CC	GGAGGTC	GGAGGTC	1.000	**C1**	**C1**	C1C1

Supplemental Figure 5. List of 403 individual animals sequenced in the population genetic study.

Collection Site	Individual	Allele 1 SNPs	Allele 2 SNPs	Prob.	Allele 1	Allele 2	Genotype
	Ne04-25CC	GGAGGTC	GGAGGTC	1.000	**C1**	**C1**	C1C1
	Ne04-26CC	GGAGGTC	GGAGGTC	1.000	**C1**	**C1**	C1C1
	Ne04-27CC	GGAGGTC	GGAGGTC	1.000	**C1**	**C1**	C1C1
	Ne04-29CC	GGAGGTC	GGAGGTC	1.000	**C1**	**C1**	C1C1
	Ne04-30CC	GGAGGTC	GGAGGTC	1.000	**C1**	**C1**	C1C1
**Pocasset**	Po3CC	GGAGGTC	GGAGGTC	1.000	**C1**	**C1**	C1C1
**MASSACHUSETTS**	Po5CC	GGAGGTC	GGAGGTC	1.000	**C1**	**C1**	C1C1
[n=15]	Po6CC	GGAGGTC	GGAGGTC	1.000	**C1**	**C1**	C1C1
	Po7CC	GGAGGTC	GGAGGTC	1.000	**C1**	**C1**	C1C1
	Po8CC	GGAGGTC	GGAGGTC	1.000	**C1**	**C1**	C1C1
	Po10CC	GGAGGTC	GGAGGTC	1.000	**C1**	**C1**	C1C1
	Po11CC	GGAGGTC	GGAGGTC	1.000	**C1**	**C1**	C1C1
	Po12CC	GGAGGTC	AGAGGTC	1.000	**C1**	**C2**	C1C2
	Po13CC	GGAGGTC	GGAGGTC	1.000	**C1**	**C1**	C1C1
	Po14CC	GGAGGTC	GGAGGTC	1.000	**C1**	**C1**	C1C1
	Po15CC	GGAGGTC	GGAGGTC	1.000	**C1**	**C1**	C1C1
	Po16CC	GGAGGTC	GGAGGTC	1.000	**C1**	**C1**	C1C1
	Po17CC	GGAGGTC	GGAGGTC	1.000	**C1**	**C1**	C1C1
	Po21CC	GGAGGTC	GGAGGTC	1.000	**C1**	**C1**	C1C1
	Po22CC	GGAGGTC	GGAGGTC	1.000	**C1**	**C1**	C1C1
**Sippewisset**	Si1-1CC	GGAGGTC	GGAGGTC	1.000	**C1**	**C1**	C1C1
**MASSACHUSETTS**	Si1-2CC	GGAGGTC	GGAGGTC	1.000	**C1**	**C1**	C1C1
[n=49]	Si4-1CC	GGAGGTC	GGAGGTC	1.000	**C1**	**C1**	C1C1
	Si4-2-1CC	GGAGGTC	GGAGGTC	1.000	**C1**	**C1**	C1C1
	Si4-2-5CC	GGAGGTC	GGAGGTC	1.000	**C1**	**C1**	C1C1
	Si4-2-7CC	GGAGGTC	GGAGGTC	1.000	**C1**	**C1**	C1C1
	Si4-2-9CC	GGAGGTC	GGAGGTC	1.000	**C1**	**C1**	C1C1
	Si4-2-11CC	GGAGGTC	GGAGGTC	1.000	**C1**	**C1**	C1C1
	Si4-2-14CC	GGAGGTC	GGAGGTC	1.000	**C1**	**C1**	C1C1
	Si4-2-17CC	GGAGGTC	GGAGGTC	1.000	**C1**	**C1**	C1C1
	Si4-3-1CC	GGAGGTC	GGAGGTC	1.000	**C1**	**C1**	C1C1
	Si4-3-3CC	GGAGGTC	GGAGGTC	1.000	**C1**	**C1**	C1C1
	Si4-3-5CC	GGAGGTC	GGAGGTC	1.000	**C1**	**C1**	C1C1
	Si4-3-6CC	GGAGGTC	GGAGGTC	1.000	**C1**	**C1**	C1C1
	Si13-15CC	GGAGGTC	GGAGGTC	1.000	**C1**	**C1**	C1C1
	Si14-1CC	GGAGGTC	GGAGGTC	1.000	**C1**	**C1**	C1C1
	Si14-2CC	GGAGGTC	GGAGGTC	1.000	**C1**	**C1**	C1C1
	Si14-3CC	GGAGGTC	GGAGGTC	1.000	**C1**	**C1**	C1C1
	Si14-4CC	GGAGGTC	GGAGGTC	1.000	**C1**	**C1**	C1C1
	Si14-5CC	GGAGGTC	GGAGGTC	1.000	**C1**	**C1**	C1C1
	Si14-6CC	GGAGGTC	GGAGGTC	1.000	**C1**	**C1**	C1C1
	Si14-7CC	GGAGGTC	GGAGGTC	1.000	**C1**	**C1**	C1C1
	Si14-8CC	GGAGGTC	GGAGGTC	1.000	**C1**	**C1**	C1C1
	Si14-9CC	GGAGGTC	GGAGGTC	1.000	**C1**	**C1**	C1C1
	Si14-10CC	GGAGGTC	GGAGGTC	1.000	**C1**	**C1**	C1C1
	Si14-11CC	GGAGGTC	GGAGGTC	1.000	**C1**	**C1**	C1C1

Supplemental Figure 5. List of 403 individual animals sequenced in the population genetic study.

Collection Site	Individual	Allele 1 SNPs	Allele 2 SNPs	Prob.	Allele 1	Allele 2	Genotype
	Si14-12CC	GGAGGTC	GGAGGTC	1.000	**C1**	**C1**	C1C1
	Si14-13CC	GGAGGTC	GGAGGTC	1.000	**C1**	**C1**	C1C1
	Si14-14CC	GGAGGTC	GGAGGTC	1.000	**C1**	**C1**	C1C1
	Si14-15CC	GGAGGTC	GGAGGTC	1.000	**C1**	**C1**	C1C1
	Si15-1CC	GGAGGTC	GGAGGTC	1.000	**C1**	**C1**	C1C1
	Si15-2CC	GGAGGTC	GGAGGTC	1.000	**C1**	**C1**	C1C1
	Si15-3CC	GGAGGTC	GGAGGTC	1.000	**C1**	**C1**	C1C1
	Si15-4CC	GGAGGTC	GGAGGTC	1.000	**C1**	**C1**	C1C1
	Si15-5CC	GGAGGTC	GGAGGTC	1.000	**C1**	**C1**	C1C1
	Si15-6CC	GGAGGTC	GGAGGTC	1.000	**C1**	**C1**	C1C1
	Si15-7CC	GGAGGTC	GGAGGTC	1.000	**C1**	**C1**	C1C1
	Si15-8CC	GGAGGTC	GGAGGTC	1.000	**C1**	**C1**	C1C1
	Si15-9CC	GGAGGTC	GGAGGTC	1.000	**C1**	**C1**	C1C1
	Si15-10CC	GGAGGTC	GGAGGTC	1.000	**C1**	**C1**	C1C1
	Si15-11CC	GGAGGTC	GGAGGTC	1.000	**C1**	**C1**	C1C1
	Si15-12CC	GGAGGTC	GGAGGTC	1.000	**C1**	**C1**	C1C1
	Si15-13CC	GGAGGTC	GGAGGTC	1.000	**C1**	**C1**	C1C1
	Si15-14CC	GGAGGTC	GGAGGTC	1.000	**C1**	**C1**	C1C1
	Si15-15CC	GGAGGTC	GGAGGTC	1.000	**C1**	**C1**	C1C1
	Si16-1CS	GGAGGTC	GTTTAAG	1.000	**C1**	**S1**	C1S1
	Si16-2CS	GGAGGTC	GTTTAAG	1.000	**C1**	**S1**	C1S1
	Si16-3CS	GGAGGTC	GTTTAAG	1.000	**C1**	**S1**	C1S1
	Si16-8CS	GGAGGTC	GGAGGTC	1.000	**C1**	**C1**	C1C1
**Meadowlands**	NJ2CC	GGAGGTC	GGAGGTC	1.000	**C1**	**C1**	C1C1
**NEW JERSEY**	NJ3CC	GGAGGTC	GGAGGTC	1.000	**C1**	**C1**	C1C1
[n=8]	NJ1-4CC	GGAGGTC	GGAGGTC	1.000	**C1**	**C1**	C1C1
	NJ1-5CC	GGAGGTC	GGAGGTC	1.000	**C1**	**C1**	C1C1
	NJ1-8CC	GGAGGTC	GGAGGTC	1.000	**C1**	**C1**	C1C1
	NJ1-9CC	GGAGGTC	GGAGGTC	1.000	**C1**	**C1**	C1C1
	NJ1-10CC	GGAGGTC	GGAGGTC	1.000	**C1**	**C1**	C1C1
	NJ1-11CC	GGAGGTC	GGAGGTC	1.000	**C1**	**C1**	C1C1
**Rhode River**	Rh1CS	GGAGGTC	GTTTAAG	1.000	**C1**	**S1**	C1S1
**MARYLAND**	Rh3SS	GTTTAAG	GTTTAAG	1.000	**S1**	**S1**	S1S1
[n=61]	Rh5SS	GTTTAAG	GTTTAAG	1.000	**S1**	**S1**	S1S1
	Rh6CS	GGAGGTC	GTTTAAG	1.000	**C1**	**S1**	C1S1
	Rh8CS	GGAGGTC	GTTTAAG	1.000	**C1**	**S1**	C1S1
	Rh9CC	GGAGGTC	GGAGGTC	1.000	**C1**	**C1**	C1C1
	Rh10CC	GGAGGTC	GGAGGTC	1.000	**C1**	**C1**	C1C1
	Rh11CS	GGAGGTC	GTTTAAG	1.000	**C1**	**S1**	C1S1
	Rh12CS	GGAGGTC	GTTTAAG	1.000	**C1**	**S1**	C1S1
	Rh13CC	GGAGGTC	GGAGGTC	1.000	**C1**	**C1**	C1C1
	Rh14SS	GTTTAAG	GTTTAAG	1.000	**S1**	**S1**	S1S1
	Rh15SS	GTTTAAG	GTTTAAG	1.000	**S1**	**S1**	S1S1
	Rh16CC	GGAGGTC	GGAGGTC	1.000	**C1**	**C1**	C1C1
	Rh17CS	GGAGGTC	GTTTAAG	1.000	**C1**	**S1**	C1S1
	Rh18CS	GGAGGTC	GTTTAAG	1.000	**C1**	**S1**	C1S1

Supplemental Figure 5. List of 403 individual animals sequenced in the population genetic study.

Collection Site	Individual	Allele 1 SNPs	Allele 2 SNPs	Prob.	Allele 1	Allele 2	Genotype
	Rh19CC	GGAGGTC	GGAGGTC	1.000	**C1**	**C1**	C1C1
	Rh21CC	GGAGGTC	GGAGGTC	1.000	**C1**	**C1**	C1C1
	Rh23CS	GGAGGTC	GTTTAAG	1.000	**C1**	**S1**	C1S1
	Rh24CS	GGAGGTC	GTTTAAG	1.000	**C1**	**S1**	C1S1
	Rh1-1SS	GTTTAAG	GTTTAAG	1.000	**S1**	**S1**	S1S1
	Rh1-2CS	GGAGGTC	GTTTAAG	1.000	**C1**	**S1**	C1S1
	Rh1-3CC	GGAGGTC	GGAGGTC	1.000	**C1**	**C1**	C1C1
	Rh1-6CC	GGAGGTC	GGAGGTC	1.000	**C1**	**C1**	C1C1
	Rh1-7CC	GGAGGTC	GGAGGTC	1.000	**C1**	**C1**	C1C1
	Rh1-8CS	GGAGGTC	GTTTAAG	1.000	**C1**	**S1**	C1S1
	Rh1-9SS	GTTTAAG	GTTTAAG	1.000	**S1**	**S1**	S1S1
	Rh1-10CC	GGAGGTC	GGAGGTC	1.000	**C1**	**C1**	C1C1
	Rh1-11CS	GGAGGTC	GTTTAAG	1.000	**C1**	**S1**	C1S1
	Rh1-13CS	GGAGGTC	GTTTAAG	1.000	**C1**	**S1**	C1S1
	Rh1-14CS	GGAGGTC	GTTTAAG	1.000	**C1**	**S1**	C1S1
	Rh1-15CS	GGAGGTC	GTTTAAG	1.000	**C1**	**S1**	C1S1
	Rh1-17CS	GGAGGTC	GTTTAAG	1.000	**C1**	**S1**	C1S1
	Rh1-18CC	GGAGGTC	GGAGGTC	1.000	**C1**	**C1**	C1C1
	Rh1-19SS	GTTTAAG	GTTTAAG	1.000	**S1**	**S1**	S1S1
	Rh1-20CC	GGAGGTC	GGAGGTC	1.000	**C1**	**C1**	C1C1
	Rh1-21CS	GGAGGTC	GTTTAAG	1.000	**C1**	**S1**	C1S1
	Rh1-23CC	GGAGGTC	GGAGGTC	1.000	**C1**	**C1**	C1C1
	Rh1-24CC	GGAGGTC	GGAGGTC	1.000	**C1**	**C1**	C1C1
	Rh1-27CC	GGAGGTC	GGAGGTC	1.000	**C1**	**C1**	C1C1
	Rh1-28SS	GTTTAAG	GTTTAAG	1.000	**S1**	**S1**	S1S1
	Rh1-30CC	GGAGGTC	GGAGGTC	1.000	**C1**	**C1**	C1C1
	Rh1-31CS	GGAGGTC	GTTTAAG	1.000	**C1**	**S1**	C1S1
	Rh1-32CC	GGAGGTC	GGAGGTC	1.000	**C1**	**C1**	C1C1
	Rh1-33CC	GGAGGTC	GGAGGTC	1.000	**C1**	**C1**	C1C1
	Rh1-34CC	GGAGGTC	GGAGGTC	1.000	**C1**	**C1**	C1C1
	Rh1-35CS	GGAGGTC	GTTTAAG	1.000	**C1**	**S1**	C1S1
	Rh1-36CS	GGAGGTC	GTTTAAG	1.000	**C1**	**S1**	C1S1
	Rh1-37CS	GGAGGTC	GTTTAAG	1.000	**C1**	**S1**	C1S1
	Rh1-38CS	GGAGGTC	GTTTAAG	1.000	**C1**	**S1**	C1S1
	Rh1-39SS	GTTTAAG	GTTTAAG	1.000	**S1**	**S1**	S1S1
	Rh1-40CC	GGAGGTC	GGAGGTC	1.000	**C1**	**C1**	C1C1
	Rh1-41CC	GGAGGTC	GGAGGTC	1.000	**C1**	**C1**	C1C1
	Rh1-43SS	GTTTAAG	GTTTAAG	1.000	**S1**	**S1**	S1S1
	Rh1-44CC	GGAGGTC	GGAGGTC	1.000	**C1**	**C1**	C1C1
	Rh1-47CS	GGAGGTC	GTTTAAG	1.000	**C1**	**S1**	C1S1
	Rh1-49CS	GGAGGTC	GTTTAAG	1.000	**C1**	**S1**	C1S1
	Rh1-52CS	GGAGGTC	GTTTAAG	1.000	**C1**	**S1**	C1S1
	Rh1-53CS	GGAGGTC	GTTTAAG	1.000	**C1**	**S1**	C1S1
	Rh1-54CC	GGAGGTC	GGAGGTC	1.000	**C1**	**C1**	C1C1
	Rh1-58CS	GGAGGTC	GTTTAAG	1.000	**C1**	**S1**	C1S1
	Rh1-60SS	GTTTAAG	GTTTAAG	1.000	**S1**	**S1**	S1S1

Supplemental Figure 5. List of 403 individual animals sequenced in the population genetic study.

Collection Site	Individual	Allele 1 SNPs	Allele 2 SNPs	Prob.	Allele 1	Allele 2	Genotype
**Baruch**	Ba1a3CC	GGAGGTC	AGAGGTG	0.970	**C1**	**C3**	C1C3
**SOUTH**	Ba1a4CC	AGAGGTG	AGAGGTG	1.000	**C3**	**C3**	C3C3
**CAROLINA**	Ba1a5CC	GGAGGTC	AGAGGTG	0.969	**C1**	**C3**	C1C3
[n=23]	Ba1a6CC	GGAGGTC	GGAGGTC	1.000	**C1**	**C1**	C1C1
	Ba1a8CC	AGAGGTG	AGAGGTG	1.000	**C3**	**C3**	C3C3
	Ba1a9CC	GGAGGTC	AGAGGTG	0.969	**C1**	**C3**	C1C3
	Ba1a10CC	GGAGGTC	GGAGGTC	1.000	**C1**	**C1**	C1C1
	Ba1a11CC	GGAGGTC	AGAGGTG	0.969	**C1**	**C3**	C1C3
	Ba1a13CC	GGAGGTC	AGAGGTG	0.969	**C1**	**C3**	C1C3
	Ba1a14CC	GGAGGTC	GGAGGTC	1.000	**C1**	**C1**	C1C1
	Ba1a15CC	GGAGGTC	AGAGGTG	0.969	**C1**	**C3**	C1C3
	Ba1a16CC	GGAGGTC	AGAGGTG	0.970	**C1**	**C3**	C1C3
	Ba1a17CC	GGAGGTC	GGAGGTC	1.000	**C1**	**C1**	C1C1
	Ba1a18CC	GGAGGTC	AGAGGTG	0.969	**C1**	**C3**	C1C3
	Ba1a19CC	GGAGGTC	AGAGGTG	0.969	**C1**	**C3**	C1C3
	Ba1c1CC	GGAGGTC	AGAGGTG	0.970	**C1**	**C3**	C1C3
	Ba1c2CC	GGAGGTC	AGAGGTG	0.969	**C1**	**C3**	C1C3
	Ba2-2CC	GGAGGTC	GGAGGTC	1.000	**C1**	**C1**	C1C1
	Ba2a4CC	GGAGGTC	GGAGGTC	1.000	**C1**	**C1**	C1C1
	Ba2a5CC	GGAGGTC	AGAGGTG	0.969	**C1**	**C3**	C1C3
	Ba2b1CC	GGAGGTC	GGAGGTC	1.000	**C1**	**C1**	C1C1
	Ba2b2CC	GGAGGTC	AGAGGTG	0.969	**C1**	**C3**	C1C3
	Ba2c6CC	GGAGGTC	AGAGGTG	0.970	**C1**	**C3**	C1C3
**San Juan**	SJ1-4CC	GGAGGTC	GGAGGTC	1.000	**C1**	**C1**	C1C1
**WASHINGTON**	SJ1-6CC	GGAGGTC	GGAGGTC	1.000	**C1**	**C1**	C1C1
[n=12]	SJ1-7CC	GGAGGTC	GGAGGTC	1.000	**C1**	**C1**	C1C1
	SJ2-4CC	GGAGGTC	GGAGGTC	1.000	**C1**	**C1**	C1C1
	SJ2-5CC	GGAGGTC	GGAGGTC	1.000	**C1**	**C1**	C1C1
	SJ2-6CC	GGAGGTC	GGAGGTC	1.000	**C1**	**C1**	C1C1
	SJ2-7CC	GGAGGTC	GGAGGTC	1.000	**C1**	**C1**	C1C1
	SJ3-4CC	GGAGGTC	GGAGGTC	1.000	**C1**	**C1**	C1C1
	SJ3-5CC	GGAGGTC	GGAGGTC	1.000	**C1**	**C1**	C1C1
	SJ3-6CC	GGAGGTC	GGAGGTC	1.000	**C1**	**C1**	C1C1
	SJ3-8CC	GGAGGTC	GGAGGTC	1.000	**C1**	**C1**	C1C1
	SJ25-3CC	GGAGGTC	GGAGGTC	1.000	**C1**	**C1**	C1C1
**Coos Bay, OR**	Co3CC	GGAGGTC	GGAGGTC	1.000	**C1**	**C1**	C1C1
**San Francisco**	CABC1CC	GGAGGTC	GGAGGTC	1.000	**C1**	**C1**	C1C1
**CALIFORNIA**	CABC2CS	GGAGGTC	GTTTAAG	1.000	**C1**	**S1**	C1S1
[n=5]	CABC3CC	GGAGGTC	GGAGGTC	1.000	**C1**	**C1**	C1C1
	CABC4CC	GGAGGTC	GGAGGTC	1.000	**C1**	**C1**	C1C1
	CABC5CS	GGAGGTC	GTTTAAG	1.000	**C1**	**S1**	C1S1
**Humboldt Bay**	CASM1CS	GGAGGTC	GTTTAAG	1.000	**C1**	**S1**	C1S1
**CALIFORNIA**	CASM3CS	GGAGGTC	GTTTAAG	1.000	**C1**	**S1**	C1S1
[n=8]	CASM9CC	GGAGGTC	GGAGGTC	1.000	**C1**	**C1**	C1C1
	CASM11CC	GGAGGTC	GGAGGTC	1.000	**C1**	**C1**	C1C1
	CASM13CS	GGAGGTC	GTTTAAG	1.000	**C1**	**S1**	C1S1

Supplemental Figure 5. List of 403 individual animals sequenced in the population genetic study.

Collection Site	Individual	Allele 1 SNPs	Allele 2 SNPs	Prob.	Allele 1	Allele 2	Genotype
	CB5CC	GGAGGTC	GGAGGTC	1.000	**C1**	**C1**	C1C1
	CC2-6CC	GGAGGTC	GGAGGTC	1.000	**C1**	**C1**	C1C1
	CD2CC	GGAGGTC	GGAGGTC	1.000	**C1**	**C1**	C1C1
**Gilkicker**	Gi1CC	GGAGGTC	AGAGGTC	1.000	**C1**	**C2**	C1C2
**UK**	Gi3CC	GGAGGTC	AGAGGTC	1.000	**C1**	**C2**	C1C2
[n=9]	Gi4CC	GGAGGTC	AGAGGTC	1.000	**C1**	**C2**	C1C2
	Gi5CC	GGAGGTC	AGAGGTC	1.000	**C1**	**C2**	C1C2
	Gi6CC	GGAGGTC	AGAGGTC	1.000	**C1**	**C2**	C1C2
	Gi7CC	GGAGGTC	AGAGGTC	1.000	**C1**	**C2**	C1C2
	Gi8CC	GGAGGTC	AGAGGTC	1.000	**C1**	**C2**	C1C2
	Gi10CC	GGAGGTC	AGAGGTC	1.000	**C1**	**C2**	C1C2
	Gi11CC	GGAGGTC	AGAGGTC	1.000	**C1**	**C2**	C1C2
**Near Salterns**	NrS1CC	GGAGGTC	AGAGGTC	1.000	**C1**	**C2**	C1C2
**UK**	NrS2CC	GGAGGTC	AGAGGTC	1.000	**C1**	**C2**	C1C2
[n=14]	NrS3CC	GGAGGTC	AGAGGTC	1.000	**C1**	**C2**	C1C2
	NrS4CC	GGAGGTC	AGAGGTC	1.000	**C1**	**C2**	C1C2
	NrS5CC	GGAGGTC	AGAGGTC	1.000	**C1**	**C2**	C1C2
	NrS6CC	GGAGGTC	AGAGGTC	1.000	**C1**	**C2**	C1C2
	NrS7CC	GGAGGTC	AGAGGTC	1.000	**C1**	**C2**	C1C2
	NrS8CC	GGAGGTC	AGAGGTC	1.000	**C1**	**C2**	C1C2
	NrS9CC	GGAGGTC	AGAGGTC	1.000	**C1**	**C2**	C1C2
	NrS10CC	GGAGGTC	AGAGGTC	1.000	**C1**	**C2**	C1C2
	NrS11CC	GGAGGTC	AGAGGTC	1.000	**C1**	**C2**	C1C2
	NrS13CC	GGAGGTC	AGAGGTC	1.000	**C1**	**C2**	C1C2
	NrS14CC	GGAGGTC	AGAGGTC	1.000	**C1**	**C2**	C1C2
	NrS15CC	GGAGGTC	AGAGGTC	1.000	**C1**	**C2**	C1C2
**Salterns**	Sa1CC	GGAGGTC	AGAGGTC	1.000	**C1**	**C2**	C1C2
**UK**	Sa3CC	GGAGGTC	AGAGGTC	1.000	**C1**	**C2**	C1C2
[n=9]	Sa4CC	GGAGGTC	AGAGGTC	1.000	**C1**	**C2**	C1C2
	Sa5CC	GGAGGTC	AGAGGTC	1.000	**C1**	**C2**	C1C2
	Sa6CC	GGAGGTC	AGAGGTC	1.000	**C1**	**C2**	C1C2
	Sa7CC	GGAGGTC	AGAGGTC	1.000	**C1**	**C2**	C1C2
	Sa8CC	GGAGGTC	AGAGGTC	1.000	**C1**	**C2**	C1C2
	Sa9CC	GGAGGTC	AGAGGTC	1.000	**C1**	**C2**	C1C2
	Sa10CC	GGAGGTC	AGAGGTC	1.000	**C1**	**C2**	C1C2
	**TOTALS**	**Allele**	**#**	**Freq**	**Geno**	**#**	**Freq**
		**C1**	618	0.77	**C1C1**	244	0.605
		**C2**	47	0.06	**C1C2**	47	0.117
		**C3**	18	0.02	**C1C3**	14	0.035
		**C4**	6	0.01	**C1C4**	2	0.005
		**S1**	117	0.15	**C3C3**	2	0.005
		**ALL**	**806**		**C1S1**	67	0.166
					**C4S1**	4	0.010
		389			**S1S1**	23	0.057
		0.9652605			**ALL**	**403**	
